# Expanded encyclopaedias of DNA elements in the human and mouse genomes

**DOI:** 10.1038/s41586-020-2493-4

**Published:** 2020-07-29

**Authors:** The ENCODE Project Consortium, Federico Abascal, Reyes Acosta, Nicholas J. Addleman, Jessika Adrian, Veena Afzal, Rizi Ai, Bronwen Aken, Jennifer A. Akiyama, Omar Al Jammal, Henry Amrhein, Stacie M. Anderson, Gregory R. Andrews, Igor Antoshechkin, Kristin G. Ardlie, Joel Armstrong, Matthew Astley, Budhaditya Banerjee, Amira A. Barkal, If H. A. Barnes, Iros Barozzi, Daniel Barrell, Gemma Barson, Daniel Bates, Ulugbek K. Baymuradov, Cassandra Bazile, Michael A. Beer, Samantha Beik, M. A. Bender, Ruth Bennett, Louis Philip Benoit Bouvrette, Bradley E. Bernstein, Andrew Berry, Anand Bhaskar, Alexandra Bignell, Steven M. Blue, David M. Bodine, Carles Boix, Nathan Boley, Tyler Borrman, Beatrice Borsari, Alan P. Boyle, Laurel A. Brandsmeier, Alessandra Breschi, Emery H. Bresnick, Jason A. Brooks, Michael Buckley, Christopher B. Burge, Rachel Byron, Eileen Cahill, Lingling Cai, Lulu Cao, Mark Carty, Rosa G. Castanon, Andres Castillo, Hassan Chaib, Esther T. Chan, Daniel R. Chee, Sora Chee, Hao Chen, Huaming Chen, Jia-Yu Chen, Songjie Chen, J. Michael Cherry, Surya B. Chhetri, Jyoti S. Choudhary, Jacqueline Chrast, Dongjun Chung, Declan Clarke, Neal A. L. Cody, Candice J. Coppola, Julie Coursen, Anthony M. D’Ippolito, Stephen Dalton, Cassidy Danyko, Claire Davidson, Jose Davila-Velderrain, Carrie A. Davis, Job Dekker, Alden Deran, Gilberto DeSalvo, Gloria Despacio-Reyes, Colin N. Dewey, Diane E. Dickel, Morgan Diegel, Mark Diekhans, Vishnu Dileep, Bo Ding, Sarah Djebali, Alexander Dobin, Daniel Dominguez, Sarah Donaldson, Jorg Drenkow, Timothy R. Dreszer, Yotam Drier, Michael O. Duff, Douglass Dunn, Catharine Eastman, Joseph R. Ecker, Matthew D. Edwards, Nicole El-Ali, Shaimae I. Elhajjajy, Keri Elkins, Andrew Emili, Charles B. Epstein, Rachel C. Evans, Iakes Ezkurdia, Kaili Fan, Peggy J. Farnham, Nina P. Farrell, Elise A. Feingold, Anne-Maud Ferreira, Katherine Fisher-Aylor, Stephen Fitzgerald, Paul Flicek, Chuan Sheng Foo, Kevin Fortier, Adam Frankish, Peter Freese, Shaliu Fu, Xiang-Dong Fu, Yu Fu, Yoko Fukuda-Yuzawa, Mariateresa Fulciniti, Alister P. W. Funnell, Idan Gabdank, Timur Galeev, Mingshi Gao, Carlos Garcia Giron, Tyler H. Garvin, Chelsea Anne Gelboin-Burkhart, Grigorios Georgolopoulos, Mark B. Gerstein, Belinda M. Giardine, David K. Gifford, David M. Gilbert, Daniel A. Gilchrist, Shawn Gillespie, Thomas R. Gingeras, Peng Gong, Alvaro Gonzalez, Jose M. Gonzalez, Peter Good, Alon Goren, David U. Gorkin, Brenton R. Graveley, Michael Gray, Jack F. Greenblatt, Ed Griffiths, Mark T. Groudine, Fabian Grubert, Mengting Gu, Roderic Guigó, Hongbo Guo, Yu Guo, Yuchun Guo, Gamze Gursoy, Maria Gutierrez-Arcelus, Jessica Halow, Ross C. Hardison, Matthew Hardy, Manoj Hariharan, Arif Harmanci, Anne Harrington, Jennifer L. Harrow, Tatsunori B. Hashimoto, Richard D. Hasz, Meital Hatan, Eric Haugen, James E. Hayes, Peng He, Yupeng He, Nastaran Heidari, David Hendrickson, Elisabeth F. Heuston, Jason A. Hilton, Benjamin C. Hitz, Abigail Hochman, Cory Holgren, Lei Hou, Shuyu Hou, Yun-Hua E. Hsiao, Shanna Hsu, Hui Huang, Tim J. Hubbard, Jack Huey, Timothy R. Hughes, Toby Hunt, Sean Ibarrientos, Robbyn Issner, Mineo Iwata, Osagie Izuogu, Tommi Jaakkola, Nader Jameel, Camden Jansen, Lixia Jiang, Peng Jiang, Audra Johnson, Rory Johnson, Irwin Jungreis, Madhura Kadaba, Maya Kasowski, Mary Kasparian, Momoe Kato, Rajinder Kaul, Trupti Kawli, Michael Kay, Judith C. Keen, Sunduz Keles, Cheryl A. Keller, David Kelley, Manolis Kellis, Pouya Kheradpour, Daniel Sunwook Kim, Anthony Kirilusha, Robert J. Klein, Birgit Knoechel, Samantha Kuan, Michael J. Kulik, Sushant Kumar, Anshul Kundaje, Tanya Kutyavin, Julien Lagarde, Bryan R. Lajoie, Nicole J. Lambert, John Lazar, Ah Young Lee, Donghoon Lee, Elizabeth Lee, Jin Wook Lee, Kristen Lee, Christina S. Leslie, Shawn Levy, Bin Li, Hairi Li, Nan Li, Shantao Li, Xiangrui Li, Yang I. Li, Ying Li, Yining Li, Yue Li, Jin Lian, Maxwell W. Libbrecht, Shin Lin, Yiing Lin, Dianbo Liu, Jason Liu, Peng Liu, Tingting Liu, X. Shirley Liu, Yan Liu, Yaping Liu, Maria Long, Shaoke Lou, Jane Loveland, Aiping Lu, Yuheng Lu, Eric Lécuyer, Lijia Ma, Mark Mackiewicz, Brandon J. Mannion, Michael Mannstadt, Deepa Manthravadi, Georgi K. Marinov, Fergal J. Martin, Eugenio Mattei, Kenneth McCue, Megan McEown, Graham McVicker, Sarah K. Meadows, Alex Meissner, Eric M. Mendenhall, Christopher L. Messer, Wouter Meuleman, Clifford Meyer, Steve Miller, Matthew G. Milton, Tejaswini Mishra, Dianna E. Moore, Helen M. Moore, Jill E. Moore, Samuel H. Moore, Jennifer Moran, Ali Mortazavi, Jonathan M. Mudge, Nikhil Munshi, Rabi Murad, Richard M. Myers, Vivek Nandakumar, Preetha Nandi, Anil M. Narasimha, Aditi K. Narayanan, Hannah Naughton, Fabio C. P. Navarro, Patrick Navas, Jurijs Nazarovs, Jemma Nelson, Shane Neph, Fidencio Jun Neri, Joseph R. Nery, Amy R. Nesmith, J. Scott Newberry, Kimberly M. Newberry, Vu Ngo, Rosy Nguyen, Thai B. Nguyen, Tung Nguyen, Andrew Nishida, William S. Noble, Catherine S. Novak, Eva Maria Novoa, Briana Nuñez, Charles W. O’Donnell, Sara Olson, Kathrina C. Onate, Ericka Otterman, Hakan Ozadam, Michael Pagan, Tsultrim Palden, Xinghua Pan, Yongjin Park, E. Christopher Partridge, Benedict Paten, Florencia Pauli-Behn, Michael J. Pazin, Baikang Pei, Len A. Pennacchio, Alexander R. Perez, Emily H. Perry, Dmitri D. Pervouchine, Nishigandha N. Phalke, Quan Pham, Doug H. Phanstiel, Ingrid Plajzer-Frick, Gabriel A. Pratt, Henry E. Pratt, Sebastian Preissl, Jonathan K. Pritchard, Yuri Pritykin, Michael J. Purcaro, Qian Qin, Giovanni Quinones-Valdez, Ines Rabano, Ernest Radovani, Anil Raj, Nisha Rajagopal, Oren Ram, Lucia Ramirez, Ricardo N. Ramirez, Dylan Rausch, Soumya Raychaudhuri, Joseph Raymond, Rozita Razavi, Timothy E. Reddy, Thomas M. Reimonn, Bing Ren, Alexandre Reymond, Alex Reynolds, Suhn K. Rhie, John Rinn, Miguel Rivera, Juan Carlos Rivera-Mulia, Brian S. Roberts, Jose Manuel Rodriguez, Joel Rozowsky, Russell Ryan, Eric Rynes, Denis N. Salins, Richard Sandstrom, Takayo Sasaki, Shashank Sathe, Daniel Savic, Alexandra Scavelli, Jonathan Scheiman, Christoph Schlaffner, Jeffery A. Schloss, Frank W. Schmitges, Lei Hoon See, Anurag Sethi, Manu Setty, Anthony Shafer, Shuo Shan, Eilon Sharon, Quan Shen, Yin Shen, Richard I. Sherwood, Minyi Shi, Sunyoung Shin, Noam Shoresh, Kyle Siebenthall, Cristina Sisu, Teri Slifer, Cricket A. Sloan, Anna Smith, Valentina Snetkova, Michael P. Snyder, Damek V. Spacek, Sharanya Srinivasan, Rohith Srivas, George Stamatoyannopoulos, John A. Stamatoyannopoulos, Rebecca Stanton, Dave Steffan, Sandra Stehling-Sun, J. Seth Strattan, Amanda Su, Balaji Sundararaman, Marie-Marthe Suner, Tahin Syed, Matt Szynkarek, Forrest Y. Tanaka, Danielle Tenen, Mingxiang Teng, Jeffrey A. Thomas, Dave Toffey, Michael L. Tress, Diane E. Trout, Gosia Trynka, Junko Tsuji, Sean A. Upchurch, Oana Ursu, Barbara Uszczynska-Ratajczak, Mia C. Uziel, Alfonso Valencia, Benjamin Van Biber, Arjan G. van der Velde, Eric L. Van Nostrand, Yekaterina Vaydylevich, Jesus Vazquez, Alec Victorsen, Jost Vielmetter, Jeff Vierstra, Axel Visel, Anna Vlasova, Christopher M. Vockley, Simona Volpi, Shinny Vong, Hao Wang, Mengchi Wang, Qin Wang, Ruth Wang, Tao Wang, Wei Wang, Xiaofeng Wang, Yanli Wang, Nathaniel K. Watson, Xintao Wei, Zhijie Wei, Hendrik Weisser, Sherman M. Weissman, Rene Welch, Robert E. Welikson, Zhiping Weng, Harm-Jan Westra, John W. Whitaker, Collin White, Kevin P. White, Andre Wildberg, Brian A. Williams, David Wine, Heather N. Witt, Barbara Wold, Maxim Wolf, James Wright, Rui Xiao, Xinshu Xiao, Jie Xu, Jinrui Xu, Koon-Kiu Yan, Yongqi Yan, Hongbo Yang, Xinqiong Yang, Yi-Wen Yang, Galip Gürkan Yardımcı, Brian A. Yee, Gene W. Yeo, Taylor Young, Tianxiong Yu, Feng Yue, Chris Zaleski, Chongzhi Zang, Haoyang Zeng, Weihua Zeng, Daniel R. Zerbino, Jie Zhai, Lijun Zhan, Ye Zhan, Bo Zhang, Jialing Zhang, Jing Zhang, Kai Zhang, Lijun Zhang, Peng Zhang, Qi Zhang, Xiao-Ou Zhang, Yanxiao Zhang, Zhizhuo Zhang, Yuan Zhao, Ye Zheng, Guoqing Zhong, Xiao-Qiao Zhou, Yun Zhu, Jared Zimmerman, Jill E. Moore, Michael J. Purcaro, Henry E. Pratt, Charles B. Epstein, Noam Shoresh, Jessika Adrian, Trupti Kawli, Carrie A. Davis, Alexander Dobin, Rajinder Kaul, Jessica Halow, Eric L. Van Nostrand, Peter Freese, David U. Gorkin, Yin Shen, Yupeng He, Mark Mackiewicz, Florencia Pauli-Behn, Brian A. Williams, Ali Mortazavi, Cheryl A. Keller, Xiao-Ou Zhang, Shaimae I. Elhajjajy, Jack Huey, Diane E. Dickel, Valentina Snetkova, Xintao Wei, Xiaofeng Wang, Juan Carlos Rivera-Mulia, Joel Rozowsky, Jing Zhang, Surya B. Chhetri, Jialing Zhang, Alec Victorsen, Kevin P. White, Axel Visel, Gene W. Yeo, Christopher B. Burge, Eric Lécuyer, David M. Gilbert, Job Dekker, John Rinn, Eric M. Mendenhall, Joseph R. Ecker, Manolis Kellis, Robert J. Klein, William S. Noble, Anshul Kundaje, Roderic Guigó, Peggy J. Farnham, J. Michael Cherry, Richard M. Myers, Bing Ren, Brenton R. Graveley, Mark B. Gerstein, Len A. Pennacchio, Michael P. Snyder, Bradley E. Bernstein, Barbara Wold, Ross C. Hardison, Thomas R. Gingeras, John A. Stamatoyannopoulos, Zhiping Weng

**Affiliations:** 1 University of Massachusetts Medical School, Program in Bioinformatics and Integrative Biology, Worcester, MA USA; 2 The Broad Institute of Harvard and MIT, Cambridge, MA USA; 3 Department of Genetics, School of Medicine, Stanford University, Palo Alto, CA USA; 4 Cold Spring Harbor Laboratory, Functional Genomics, Cold Spring Harbor, NY USA; 5 Altius Institute for Biomedical Sciences, Seattle, WA USA; 6 Department of Medicine, University of Washington School of Medicine, Seattle, WA USA; 7 Department of Cellular and Molecular Medicine, Institute for Genomic Medicine, Stem Cell Program, Sanford Consortium for Regenerative Medicine, University of California, San Diego, La Jolla, CA USA; 8 Program in Computational and Systems Biology, Massachusetts Institute of Technology, Cambridge, MA USA; 9 Center for Epigenomics, Department of Cellular and Molecular Medicine, University of California, San Diego, La Jolla, CA USA; 10 Ludwig Institute for Cancer Research, University of California, San Diego, La Jolla, CA USA; 11 Institute for Human Genetics, Department of Neurology, University of California, San Francisco, San Francisco, CA USA; 12 Genomics Analysis Laboratory, The Salk Institute for Biological Studies, La Jolla, CA USA; 13 HudsonAlpha Institute for Biotechnology, Huntsville, AL USA; 14 Division of Biology and Biological Engineering, California Institute of Technology, Pasadena, CA USA; 15 Department of Developmental and Cell Biology, University of California Irvine, Irvine, CA USA; 16 Department of Biochemistry and Molecular Biology, The Pennsylvania State University, University Park, PA USA; 17 Environmental Genomics and Systems Biology Division, Lawrence Berkeley National Laboratory, Berkeley, CA USA; 18 Department of Genetics and Genome Sciences, Institute for Systems Genomics, UConn Health, Farmington, CT USA; 19 Département de Biochimie et Médecine Moléculaire, Université de Montréal, Montréal, Quebec, Canada; 20 Division of Experimental Medicine, McGill University, Montreal, Quebec Canada; 21 Institut de Recherches Cliniques de Montréal (IRCM), Montréal, Quebec Canada; 22 Department of Biological Science, Florida State University, Tallahassee, FL USA; 23 Department of Biochemistry, Molecular Biology and Biophysics, University of Minnesota Medical School, Minneapolis, MN USA; 24 Yale University, New Haven, CT USA; 25 Biological Sciences, University of Alabama in Huntsville, Huntsville, AL USA; 26 Department of Genetics, School of Medicine, Yale University, New Haven, CT USA; 27 Department of Human Genetics, Institute for Genomics and Systems Biology, The University of Chicago, Chicago, IL USA; 28 Tempus Labs, Chicago, IL USA; 29 US Department of Energy Joint Genome Institute, Lawrence Berkeley National Laboratory, Berkeley, CA USA; 30 School of Natural Sciences, University of California, Merced, Merced, CA USA; 31 Department of Biology, Massachusetts Institute of Technology, Cambridge, MA USA; 32 HHMI and Program in Systems Biology, University of Massachusetts Medical School, Worcester, MA USA; 33 University of Colorado Boulder, Boulder, CO USA; 34 Howard Hughes Medical Institute, The Salk Institute for Biological Studies, La Jolla, CA USA; 35 Computer Science and Artificial Intelligence Laboratory, Massachusetts Institute of Technology, Cambridge, MA USA; 36 Department of Genetics and Genomic Sciences, Icahn School of Medicine at Mount Sinai, New York, NY USA; 37 Department of Genome Sciences, University of Washington School of Medicine, Seattle, WA USA; 38 Bioinformatics and Genomics Program, Centre for Genomic Regulation (CRG), The Barcelona Institute of Science and Technology and Universitat Pompeu Fabra, Barcelona, Spain; 39 Department of Biochemistry and Molecular Medicine, Norris Comprehensive Cancer Center, Keck School of Medicine, University of Southern California, Los Angeles, CA USA; 40 Comparative Biochemistry Program, University of California, Berkeley, CA USA; 41 Cardiovascular Institute, Stanford School of Medicine, Stanford, CA USA; 42 Broad Institute and Department of Pathology, Massachusetts General Hospital and Harvard Medical School, Boston, MA USA; 43 Department of Thoracic Surgery, Clinical Translational Research Center, Shanghai Pulmonary Hospital, The School of Life Sciences and Technology, Tongji University, Shanghai, China; 44 Bioinformatics Program, Boston University, Boston, MA USA; 45 MGH, Boston, MA USA; 46 Dana-Farber Cancer Institute, Boston, MA USA; 47 Harvard Medical School, Boston, MA USA; 48 Boston Children’s Hospital, Boston, MA USA; 49 Harvard University, Cambridge, MA USA; 50 Max Planck Institute for Molecular Genetics, Department of Genome Regulation, Berlin, Germany; 51 IRSD, Université de Toulouse, INSERM, INRA, ENVT, UPS, U1220, CHU Purpan, CS60039 Toulouse, France; 52 Skolkovo Institute for Science and Technology, Moscow, Russia; 53 Cold Spring Harbor Laboratory, Woodbury, NY USA; 54 Department of Clinical Research, University of Bern, Bern, Switzerland; 55 International Institute of Molecular and Cell Biology, Warsaw, Poland; 56 Department of Cellular and Molecular Medicine, Institute of Genomic Medicine, University of California at San Diego, San Diego, CA USA; 57 Department of Pharmaceutical Sciences, St. Jude Children’s Research Hospital, Memphis, TN USA; 58 National Human Genome Research Institute, National Institutes of Health, Bethesda, MD USA; 59 Department of Biostatistics and Bioinformatics, Duke University, Durham, NC USA; 60 Center for Genomic and Computational Biology, Duke University, Durham, NC USA; 61 Department of Chemistry and Biochemistry, Department of Cellular and Molecular Medicine, UC San Diego, La Jolla, CA USA; 62 Department of Biochemistry and Molecular Genetics, Northwestern University Feinberg School of Medicine, Chicago, IL USA; 63 Penn State Health Milton S. Hershey Medical Center, Hershey, PA USA; 64 Department of Computational Medicine and Bioinformatics, University of Michigan, Ann Arbor, MI USA; 65 Department of Human Genetics, University of Michigan, Ann Arbor, MI USA; 66 Department of Molecular and Cellular Physiology, School of Medicine, Stanford University, Palo Alto, CA USA; 67 Terrence Donnelly Centre for Cellular and Biomolecular Research, University of Toronto, Toronto, Ontario Canada; 68 Department of Radiation Oncology, School of Medicine, Stanford University, Palo Alto, CA USA; 69 Division of General Surgery, Section of Transplant Surgery, School of Medicine, Washington University, St. Louis, MO USA; 70 Department of Biochemistry and Molecular Biology, School of Basic Medical Sciences, Southern Medical University, Guangzhou, China; 71 Guangdong Provincial Key Laboratory of Single Cell Technology and Application, Guangzhou, China; 72 Department of Cell Biology & Physiology, University of North Carolina at Chapel Hill, Chapel Hill, NC USA; 73 Thurston Arthritis Research Center, University of North Carolina at Chapel Hill, Chapel Hill, NC USA; 74 Department of Molecular Genetics, University of Toronto, Toronto, Ontario, Canada; 75 School of Medicine, Jiangsu University, Zhenjiang, China; 76 Department of Molecular Genetics, Donnelly Centre, University of Toronto, Toronto, Ontario Canada; 77 Division of Medical Genetics, University of Washington School of Medicine, Seattle, WA USA; 78 Fred Hutchinson Cancer Research Center, Seattle, WA USA; 79 University of Massachusetts Amherst, Amherst, MA USA; 80 Institute for Infocomm Research, Singapore, Singapore; 81 Simon Fraser University, Burnaby, British Columbia Canada; 82 Center for Functional Cancer Epigenetics, Dana-Farber Cancer Institute, Boston, MA USA; 83 Department of Data Sciences, Dana-Farber Cancer Institute and Harvard T.H. Chan School of Public Health, Boston, MA USA; 84 Center for Public Health Genomics, University of Virginia, Charlottesville, VA USA; 85 Molecular Pathology Unit & Cancer Center, Massachusetts General Hospital and Harvard Medical School, Boston, MA USA; 86 Department of Biostatistics and Bioinformatics, Moffitt Cancer Center, Tampa, FL USA; 87 Institute for Stem Cell Biology and Regenerative Medicine, Stanford University, Stanford, CA USA; 88 Department of Medicine, Brigham and Women’s Hospital and Harvard Medical School, Boston, MA USA; 89 Department of Statistics, Medical Sciences Center, University of Wisconsin - Madison, Madison, WI USA; 90 Department of Biostatistics and Medical Informatics, University of Wisconsin - Madison, Madison, WI USA; 91 Department of Mathematical Sciences, University of Texas at Dallas, Richardson, TX USA; 92 Department of Statistics, University of Nebraska-Lincoln, Lincoln, NE USA; 93 Department of Biomedical Informatics, The Ohio State University, Columbus, OH USA; 94 Department of Cell and Regenerative Biology, UW-Madison Blood Research Program, Carbone Cancer Center, University of Wisconsin School of Medicine and Public Health, University of Wisconsin, Madison, WI USA; 95 Wellcome Sanger Institute, Cambridge, UK; 96 Program in Computational Biology, Memorial Sloan Kettering Cancer Center, New York, NY USA; 97 Department of Integrative Biology and Physiology, University of California Los Angeles, Los Angeles, CA USA; 98 McKusick-Nathans Institute of Genetic Medicine, Johns Hopkins University, Baltimore, MD USA; 99 Department of Biomedical Engineering, Johns Hopkins University, Baltimore, MD USA; 100 European Molecular Biology Laboratory, European Bioinformatics Institute, Wellcome Genome Campus, Cambridge, United Kingdom; 101 University of California, Santa Cruz, Santa Cruz, CA USA; 102 Center for Integrative Genomics, University of Lausanne, Lausanne, Switzerland; 103 Centro Nacional de Investigaciones Cardiovasculares (CNIC) and CIBER de Enfermedades Cardiovasculares (CIBERCV), Madrid, Spain; 104 Spanish National Cancer Research Centre (CNIO), Madrid, Spain; 105 Brunel University London, London, UK; 106 King’s College London, Guy’s Hospital, London, UK; 107 ELIXIR Hub, Wellcome Genome Campus, Cambridge, UK; 108 Institute of Cancer Research, Chester Betty Labs, London, UK; 109 Center for Vaccines and Immunology, University of Georgia, Athens, GA USA; 110 Center for Molecular Medicine and Department of Biochemistry and Molecular Biology, University of Georgia, Athens, GA USA; 111 Gift of Life Donor Program, Philadelphia, PA USA; 112 American Society for Radiation Oncology, Arlington, VA USA; 113 National Cancer Institute, National Institutes of Health, Bethesda, MD USA; 114 Leidos Biomedical, Inc, Frederick, MD USA; 115 National Disease Research Interchange (NDRI), Philadelphia, PA USA; 116 National Human Genome Research Institute, National Institutes of Health, Bethesda, MD USA; 117 4407 Puller Drive, Kensington, MD USA; 118 Department of Chemistry and Biochemistry, University of California, San Diego, La Jolla, CA USA; 119 Program in Computational Biology and Bioinformatics, Yale University, New Haven, CT USA

**Keywords:** Data integration, Epigenomics, Functional genomics

## Abstract

The human and mouse genomes contain instructions that specify RNAs and proteins and govern the timing, magnitude, and cellular context of their production. To better delineate these elements, phase III of the Encyclopedia of DNA Elements (ENCODE) Project has expanded analysis of the cell and tissue repertoires of RNA transcription, chromatin structure and modification, DNA methylation, chromatin looping, and occupancy by transcription factors and RNA-binding proteins. Here we summarize these efforts, which have produced 5,992 new experimental datasets, including systematic determinations across mouse fetal development. All data are available through the ENCODE data portal (https://www.encodeproject.org), including phase II ENCODE^1^ and Roadmap Epigenomics^2^ data. We have developed a registry of 926,535 human and 339,815 mouse candidate *cis*-regulatory elements, covering 7.9 and 3.4% of their respective genomes, by integrating selected datatypes associated with gene regulation, and constructed a web-based server (SCREEN; http://screen.encodeproject.org) to provide flexible, user-defined access to this resource. Collectively, the ENCODE data and registry provide an expansive resource for the scientific community to build a better understanding of the organization and function of the human and mouse genomes.

## Abstract (web summary)

The authors summarize the data produced by phase III of the Encyclopedia of DNA Elements (ENCODE) project, a resource for better understanding of the human and mouse genomes.

## Main

The human genome comprises a vast repository of DNA-encoded instructions that are read, interpreted, and executed by the cellular protein and RNA machinery to enable the diverse functions of living cells and tissues. The ENCODE Project aims to delineate precisely and comprehensively the segments of the human and mouse genomes that encode functional elements^1,3–6^. Operationally, functional elements are defined as discrete, linearly ordered sequence features that specify molecular products (for example, protein-coding genes or noncoding RNAs) or biochemical activities with mechanistic roles in gene or genome regulation (for example, transcriptional promoters or enhancers)^5^. Commencing with the ENCODE Pilot Project in 2003 (which focused on a defined 1% of the human genome sequence^4^) and scaling to the entire genome in a production phase II that began in 2007^1^, ENCODE has applied a succession of state-of-the-art assays to identify likely functional elements with increasing precision across an expanding range of cellular and biological contexts. To capitalize on the value of the laboratory mouse, *Mus musculus*, for both comparative functional genomic analysis and modelling of human biology, a Mouse ENCODE Project of more limited scope was initiated in 2009^6^. An accompanying Perspective^7^ provides further context for the evolution of the ENCODE Project and describes how ENCODE data are being used to illuminate both basic biological and biomedical questions that intersect genome structure and function.

Beginning in 2012, both the human and mouse ENCODE Projects initiated programs to broaden and deepen their respective efforts to discover and annotate functional elements, and to systematize the production, curation, and dissemination of ENCODE data with the aim of broadly empowering the scientific community. ENCODE data have served as an enabling interface between the human genome sequence and its application to biomedical research because of both the range of biological and biochemical features encompassed by ENCODE assays and the breadth and depth with which these assays have been applied across cell and tissue contexts. ENCODE has now expanded on both of these axes by (i) incorporating new assays such as RNA-binding-protein localization and chromatin looping; (ii) increasing the depths at which current assays such as transcription factor chromatin immunoprecipitation and sequencing (ChIP–seq) interrogate reference cell lines; and (iii) collecting data over a greatly expanded biological range, with an emphasis on primary cells and tissues. In addition, ENCODE has now incorporated and uniformly processed the substantial data from the Roadmap Epigenomics Project^2^ that conform to ENCODE standards (see Methods).

Here, we describe the generation of nearly 6,000 new experiments (4,834 using human tissues or cells and 1,158 using mouse tissues or cells) in phase III that have extended previous phases of ENCODE in order to define and annotate diverse classes of functional elements in the human and mouse genomes (Table 1). Whereas many experiments during earlier phases of ENCODE used model cell lines, a major goal of phase III was to broaden coverage of primary cells and tissues. Together, the ENCODE–Roadmap Encyclopedia now encompasses 503 biological cell or tissue types from more than 1,369 biological sample sources (biosamples) (Extended Data Table 1). As a new feature of ENCODE, we have systematically integrated DNA accessibility and chromatin modification data to create a categorized registry of candidate *cis*-regulatory elements (cCREs) in both the human and mouse genomes. We have also developed a new web-based interface called SCREEN to facilitate access to the human and mouse registries and to facilitate their application to diverse biological problems.

**Table 1:** Summary of ENCODE3 production

Assay	Description and details	No. of experiments	No. of targets	No. of biosamples
**DNA binding and chromatin modification**				
ChIP–seq	Chromatin immunoprecipitation sequencing			
	Chromatin-associated proteins	1,343	653	151
	Histone marks	1,082	13	158
**Transcription**				
RNA-seq	RNA sequencing			
	Total RNA	224	–	209
	polyA RNA	116	–	106
	microRNA	112	–	108
	small RNA	86	–	85
	Knockdown/knockout RNA sequencing			
	CRISPR	50	28	2
	CRISPR interference	77	74	1
	Short hairpin RNA	523	253	2
	Small inhibitory RNA	54	35	3
scRNA-seq	Single-cell RNA sequencing	13	—	12
RAMPAGE	RNA annotation and mapping of promoters for the analysis of gene expression	155	—	154
**Chromatin accessibility**				
DNase-seq	DNase I cleavage site sequencing	246	—	246
	DNase-seq of genetically modified cells	46	28	1
ATAC-seq	Assay for transposase accessible chromatin using sequencing	129	—	129
**DNA methylation**				
WGBS	Whole-genome bisulfite sequencing	132	—	129
DNAme array	DNA methylation profiling by array	154	—	151
**RNA binding**				
eCLIP	Enhanced UV crosslinking and immunoprecipitation of RNA binding proteins (RBPs) followed by sequencing to identify bound RNAs in cells	170	117	3
RNA Bind-n-seq	In vitro method for quantifying RBP–RNA interactions and identifying binding motifs	78	78	—
**3D chromatin structure**				
ChIA-PET	Chromatin interaction analysis by paired-end tag sequencing	49	6	29
Hi-C	Genome-wide chromosome conformation capture (all-versus-all interactions)	33	—	33
**Replication timing**				
Repli-chip	Measures DNA replication timing using microarrays	36	—	30
Repli-seq	Measures DNA replication timing using sequencing	14	—	14

Control experiments were excluded from this table but can be found in Extended Data Table 1. Counts were obtained on 1 December 2019.

Across multiple data types, the increase in the scale of experimental data has provided new insights into genome organization and function, and catalysed new capabilities for deriving biological understandings and principles, as illustrated below and detailed in accompanying papers^7–16^. In summary, we:

- Define core gene sets that correspond to major cell types using extensive new maps of RNA transcripts in a broad range of primary cell types^8^.
- Describe an expansive new genomic compartment of DNA elements that encode recognition sites for RNA-binding proteins, providing new insights into post-transcriptional regulation^9^.
- Deeply map the co-occupancy patterns of human transcription factors in reference cell types and connect these with key biological features of promoters and distal enhancers^10^.
- Greatly increase the cell and tissue range, genomic resolution, and biological annotation of human DNase I-hypersensitive sites^11^ and transcription factor footprints^12^.
- Characterize the landscape of 3D chromatin interactions across 24 different cell types^13^.
- Expand annotation of mouse chromatin modification, DNA accessibility, DNA methylation, and RNA transcription landscapes in early developmental stages not readily accessible in human^14–17^.

To enhance the utility and accessibility of ENCODE data for studies of gene regulation, in this report, we have now:

- Systematically integrated DNA accessibility and chromatin modification data to create a categorized and expandable registry of cCREs in the human and mouse genomes.
- Developed a new web-based interface (SCREEN) to facilitate access to the human and mouse registries and to empower their application to diverse biological problems.

## Expanding human and mouse ENCODE

**Fig. 1:**
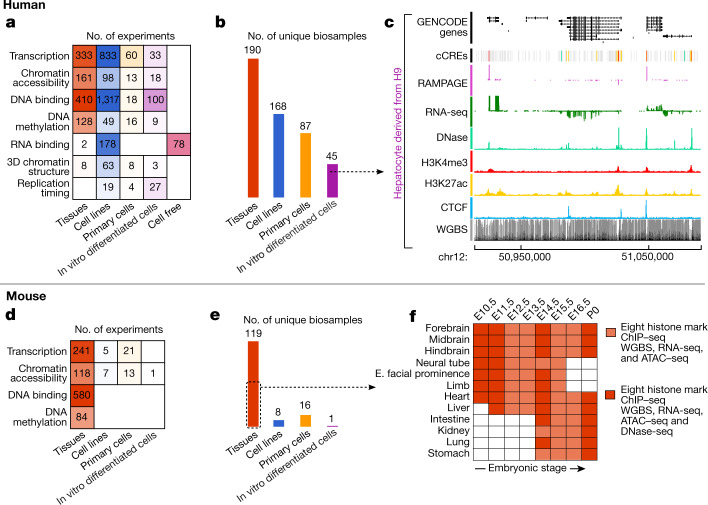
ENCODE phase III data production. Human (**a**–**c**) and mouse (**d**–**f**) experiments performed during ENCODE phase III with data released on the ENCODE Portal, sorted by type of assay (**a**, **d**) or type of biosample (**b**, **e**). **c**, An illustrative human locus shows signals from several data types. **f**, The mouse fetal developmental matrix shows the tissues and stages at which epigenetic features and transcriptomes were assayed.

We sought to develop the human Encyclopedia of DNA Elements along three axes by: (i) expanding established chromatin structure and histone modification assays to new and diverse cellular contexts, chiefly primary cells and tissues; (ii) adopting and scaling up additional biochemical assays to address gaps in the annotation of DNA-encoded elements, particularly transcribed elements; and (iii) increasing the molecular depth of assays for transcription factors (TFs), co-factors, and other chromatin-associated proteins to deeply annotate prioritized reference cell types (Fig. 1a–c, Table 1). In parallel with the human ENCODE effort, we aimed to expand the range and utility of mouse ENCODE by applying a set of assays for RNA transcription, DNA methylation, chromatin modification, and DNA accessibility to embryonic, fetal and neonatal tissues with an emphasis on the brain, and to an expanded range of juvenile and adult tissues (Fig. 1d–f, Table 1).

**Fig. 2:**
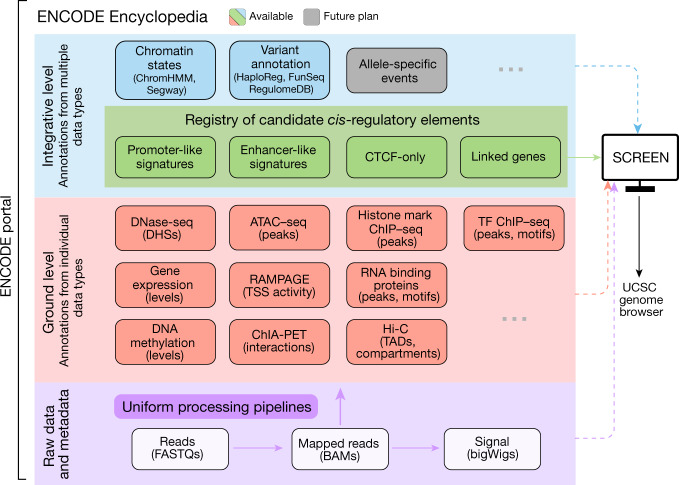
Overview of the ENCODE Encyclopedia with a registry of candidate *cis*-regulatory elements. The ENCODE Encyclopedia consists of ground-level and integrative-level annotations that use data processed by the uniform processing pipelines. SCREEN integrates all levels of annotations and raw data and allows users to visualize them in the UCSC genome browser.

Overall, compared with our previous reports^1,5,6^, the third phase of ENCODE expanded by more than fourfold the number of cell types and tissues assayed and more than twofold the number of experimental datasets produced (Extended Data Table 1). Below we briefly summarize the key ENCODE data types and the collection of these data into a primary ENCODE Encyclopedia (Fig. 2), from which the registry of candidate *cis*-regulatory elements described in the next section is derived. Uniform processing methods and data standards were developed for each data type and applied consistently to all biological samples interrogated by a particular assay to produce both signal data that vary in a continuous fashion along the genome, and discrete elements detected as intervals of significant enrichment in the primary signal. All data and protocols are openly available at the ENCODE Portal (https://www.encodeproject.org/). Furthermore, all ENCODE data are now also available as resident data sets within a major public computing cloud (https://registry.opendata.aws/encode-project/). We have continued to expand the repertoire of tools for data analysis, adopting widely used external tools whenever possible and developing new tools as needed (https://www.encodeproject.org/software/).

### Transcribed elements

The universe of transcribed elements—the transcriptome—has become a common tool for the molecular phenotyping of cells and tissues and serves as a framework for diverse computational analyses of cellular states^18^. The transcriptome is deeply complex, and both new isoforms of known genes and short RNA species such as enhancer RNAs continue to be discovered^18^.

During this phase of ENCODE, we developed an approach called RNA Annotation and Mapping of Promoters for the Analysis of Gene Expression (RAMPAGE)^19^ that can (i) position transcriptional start sites (TSSs) with single-nucleotide resolution; (ii) generate accurate quantitative and reproducible measurements of promoter-specific RNA expression; and (iii) precisely connect 5′-transcription initiation sites with splicing isoforms, thus providing a previously unavailable connection between promoter regulation and spliced products over long genomic intervals. RAMPAGE also enables the annotation of previously intractable classes of RNA transcript that emanate from repetitive elements^20^. We deepened human transcriptome annotations by combining RAMPAGE with short (below 200 nucleotides (nt)) and long (more than 200 nt) RNA sequencing (RNA-seq) performed on approximately 200 biosamples (Supplementary Table 1a). We also systematically expanded the mouse transcriptome by performing bulk RNA-seq and microRNA-seq on 17 developing tissues, some on multiple embryonic days, augmented by single-cell RNA-seq on the developing limb^16,21^ (Supplementary Table 1b, c). These new data enhance and expand our knowledge of transcribed elements, including precise mapping of promoters and splicing isoforms to improve gene and transcript annotation, as well as deepening our knowledge of diverse noncoding transcripts. Furthermore, they reveal sets of genes that define a distinctive molecular phenotype for the major classes of cell types^8^.

### RNA-binding proteins

Genes that encode RNA-binding proteins (RBPs) are one of the largest gene families in the human genome, comprising approximately 10% of all protein-coding genes^22^. The RNA sequences and structures recognized by RBPs are encoded by the underlying genomic sequence, and thus represent a class of functional sequence elements not previously explored by ENCODE (Table 1). Using an enhanced crosslinking and immunoprecipitation assay (eCLIP)^23^, we identified the binding sites for 150 RBPs in two extensively assayed ENCODE cell lines, K562 and HepG2, and further validated the RNA targets recognized by each RBP by knocking down the RBP and performing RNA-seq^9^ (Supplementary Table 2). We also developed an in vitro binding assay and applied it to 78 RBPs, demonstrating that the binding sites of most RBPs in K562 or HepG2 cells are consistent with their in vitro RNA sequence specificity^24^. Subcellular localization patterns of 274 RBPs revealed extensive compartmentalization, indicative of widespread organelle-specific RNA activities (http://rnabiology.ircm.qc.ca/RBPImage/). These data open a window into the post-transcriptional roles and mechanisms of RBPs in determining the levels of specific transcripts.

### Chromatin-associated proteins

Despite intensive efforts, the in vivo occupancy sites for most of the more than 1,600 sequence-specific transcription factors and other chromatin-associated proteins encoded by the human genome remain to be defined. Recognition motifs for a growing assemblage of TFs have been compiled on the basis of ChIP–seq and in vitro assays^25^; however, these collections are far from complete, particularly for factors with extended recognition sequences. Notably, sequence motifs alone do not capture which motif instances are occupied in vivo, nor do they identify indirect localization events wherein one or more TFs are associated with an ‘anchor’ factor that is directly bound to the genome^26^. To enable detailed analysis of both in vivo recognition motifs and combinatorial occupancy patterns for human transcriptional regulators, we applied ChIP–seq to densely map the locations of 662 chromatin-associated proteins, including classical RNA Pol II-associated factors such as TFIID, in reference cell types (Supplementary Table 3). These new data not only expand our knowledge of the binding patterns of TFs, but also reveal patterns of extensive co-occupancy among human TFs. Furthermore, the integration of ENCODE TF binding elements with chromatin and RNA transcription data provides connections with key biological features of promoters and distal enhancers and insights into the organization of chromatin loops and gene domains^10^.

### DNase I hypersensitive sites and footprints

We have expanded the biological range and molecular resolution of ENCODE DNase I hypersensitive sites (DHSs) and DNase I footprint annotations. DHSs are the hallmark of active or poised *cis*-regulatory elements, including enhancers, silencers, insulators, and the core components of composite elements such as locus control regions. Using an improved DNase treatment followed by sequencing (DNase-seq) assay that requires only small numbers of input cells, we expanded ENCODE human DHS maps by more than 200 different cell types and states, chiefly primary cells and tissues^11^ (Supplementary Table 4a). By incorporating both a multi-tissue developmental series and a larger range of adult tissues (Supplementary Table 4b), we also greatly expanded mouse DHS maps^17^. We have consolidated the full range of DNase-seq data from ENCODE and the Roadmap Epigenomics Project across hundreds of biosamples, and thereby catalogued reference indices of about 3.6 million consensus DHSs within the human genome^11^ and about 1.8 million consensus DHSs within the mouse genome. The diversity of cell types and states enabled systematic categorization of coordinated tissue-selective DHS activation patterns, which were then used to annotate DHSs, genes, and genetic variation^11^. The number of human ENCODE biosamples with deep DNase-seq data (more than 200 million uniquely mapped reads) was tripled to more than 300, enabling delineation of 4.4 million consensus human DNase I footprints within DHSs, enhanced annotation of tissue selectivity, and identification of functional variants that directly affect regulatory factor occupancy^11,12^. These extensive indexes of DHSs and footprints, systematically annotated by their tissue-selective patterns of activation, provide unprecedented resources for detailed studies of gene regulation and investigation of genetic variants associated with diseases and complex traits.

### Transposase accessible regions

During the course of the project, a new technique, assay for transposase-accessible chromatin using sequencing (ATAC–seq)^27^, was adopted to profile chromatin accessibility genome-wide in 66 mouse tissues and cell types that spanned 8 developmental stages (Fig. 1f, Supplementary Table 4d). More than 500,000 regions in the mouse genome that were marked as accessible chromatin were temporally mapped across fetal development. Human orthologues of accessible regions in fetal mouse tissues are enriched for human disease-associated variation in a tissue-restricted manner^14^. We also applied ATAC–seq to 15 additional mouse tissues and cell types^28^ and 48 primary tissues from human adults (Supplementary Table 4c, d). Not only do these data expand the range of biosamples for which there are maps of accessible chromatin, but when integrated with histone modifications and other epigenetic signals, they reveal the activation of cCREs across cell types^28^.

### Histone marks and chromatin-modifying proteins

The previous phase of ENCODE focused on the connection of the types and number of histone modifications with identified elements of genome function found in various cell types^29,30^. In this phase, we standardized ChIP–seq assays for 11 histone modifications and 2 common histone variants (Supplementary Table 5a) and profiled these across 79 human cell and tissue types. We also profiled histone modifications across 12 mouse tissues over 8 developmental stages from embryonic day 10.5 until birth^14^ (Supplementary Table 5b). To deepen insights into the genesis of histone modification patterns in human cells, we also profiled a panel of 22 proteins involved in the deposition or recognition of histone modifications (Supplementary Table 5c). These new data not only expand the numbers of cell types and types of histone modifications interrogated, but also provide insights into the actions of so-called chromatin ‘readers’ and ‘writers’, many of which have been implicated in developmental disorders and cancer progression.

### DNA methylation

The annotation of human DNA methylation was deepened by applying whole-genome bisulfite sequencing (WGBS^31^, Table 1) to 48 cell and tissue types, and broadened by profiling approximately 154 additional biological contexts using methylation-aware DNA microarrays (Supplementary Table 6a). To expand mouse DNA methylation annotations, we used WGBS to map methylation patterns in 12 mouse tissues at 9 developmental stages, collecting a total of 84 whole-genome methylation maps^15^ (Fig. 1f, Supplementary Table 6b). The WGBS data provide an unbiased view of DNA methylation patterns and their dynamics across mouse development^15^.

### Chromatin looping

Maps of chromatin interaction frequencies and genome connectivity provide information on physical links among regulatory elements and target genes at different levels of cellular organization. We generated Hi-C chromatin conformation maps for 33 human tissue and cell types^32^, providing insights into the positions of chromosome compartments^33^ and topologically associating domains^34,35^ (Supplementary Table 7a). Furthermore, we investigated in detail the roles of the genome organizing factor CTCF and the cohesin subunit RAD21, which frequently co-localize to influence chromatin interactions. We systematically localized RAD21 in 24 diverse cell lines (Supplementary Table 7b) using chromatin interaction analysis via paired-end tag sequencing (ChIA-PET^36^) (Table 1), which measures the proximity and frequency of contacts between RAD21-bound regions. These data were also integrated with the profiles of acetylated lysine 27 on histone H3 (H3K27ac) as well as RNA-seq data from the same cell types. Analysis of these data revealed that many 3D chromatin interactions vary across cell types and that these ‘variable’ interactions were correlated with gene expression and enriched in variants identified in genome-wide association studies^13^.

### DNA replication timing

DNA replication timing provides insights into both gene regulation and spatiotemporal genome compartmentalization. We measured replication timing during fate commitment of human embryonic stem cells, yielding 50 data sets for 26 cell types representing the embryonic layers endoderm, mesoderm, ectoderm, and neural crest^37^ (Supplementary Table 8). The analysis of these data sets revealed that the developmental lineage of each cell type could be recapitulated on the basis of its replication timing. ENCODE replication timing data have also been used to build background mutation models to study the somatic mutation process^38^ and to construct novel, cell type-specific regulatory networks^39^.

## A registry of DNA elements

The comprehensive discovery and annotation of *cis*-regulatory elements encoded within the human and mouse genomes are major goals of ENCODE^1,4–6^. The cardinal biochemical features of active or poised enhancer, promoter, or insulator elements are focal chromatin biochemical marks and heightened DNA accessibility, which result from the binding of sequence-specific regulatory factors in place of a canonical nucleosome. This increased accessibility can be detected as hypersensitivity to nucleases as mapped by DNase-seq^40^ or susceptibility to transposase insertions as mapped by ATAC–seq^27^. In addition to nuclease hypersensitivity, active or poised enhancers and promoters typically exhibit characteristic histone modification signatures on flanking nucleosomes^4,41^, whereas mammalian insulator elements are occupied by CTCF^42^. Thus, the DNase-seq signal can be integrated with ChIP–seq of trimethylated lysine 4 on histone H3 (H3K4me3)—a core histone modification that is characteristic of transcribing promoters^41^—to annotate active and poised promoters^43^. Similarly, H3K27ac, combined with relative paucity of H3K4me3 surrounding a DHS, has been strongly associated with active enhancer function at the underlying DNA element^44^.

**Fig. 3:**
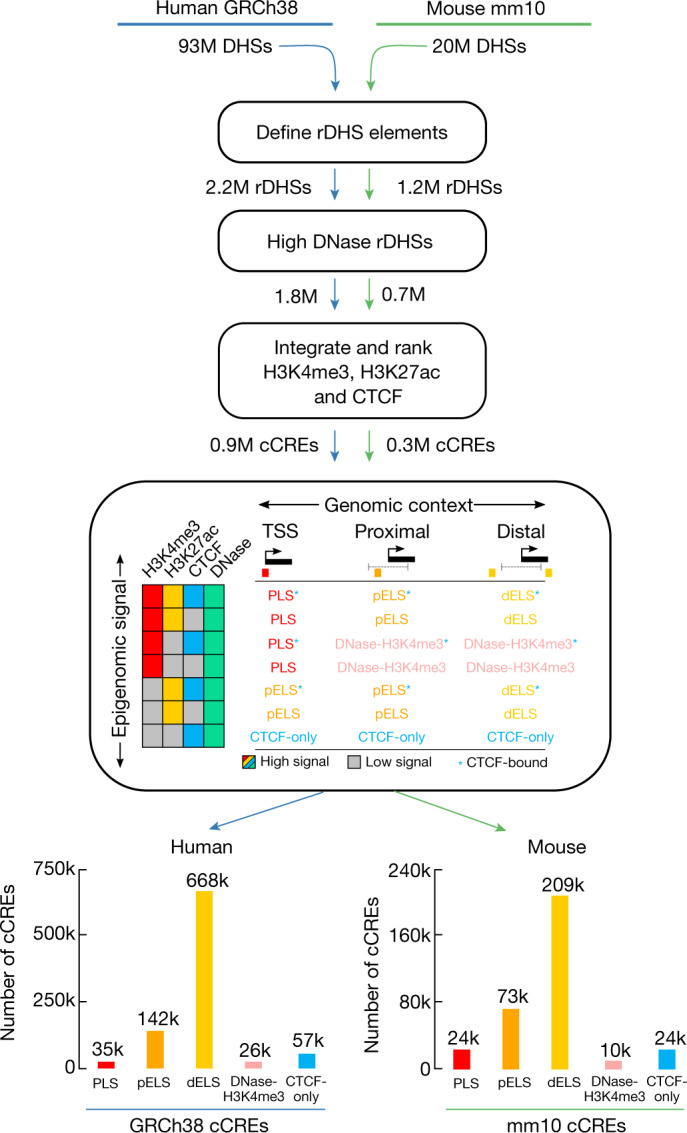
Selection and classification of cCREs to build the registry of candidate *cis*-regulatory elements. We began by filtering and clustering DNase peaks to create representative DHSs (rDHSs). We then selected those rDHSs with high DNase signal (maximal *Z*-score or max-*Z* across all biosamples with data; see Methods) and high signal for at least one other assay (H3K4me3, H3K27ac or CTCF) to be cCREs. In total, we defined 926,535 cCREs in human and 339,815 cCREs in mouse. On the basis of combinations of signal and genomic context, we classified cCREs into one of these groups: PLS, pELS, dELS, DNase–H3K4me3, or CTCF-only, and their counts are indicated (k, thousand; M, million). Human and mouse silhouettes were adapted under Public Domain Mark 1.0 and Public Domain Dedication 1.0 licenses, respectively.

We have applied these simple core biochemical signatures, integrated with the GENCODE annotation of TSSs, to create an initial registry of human and mouse cCREs that show signatures of activity, or of being poised for activity, in one or more ENCODE biosamples. Using the classification system (Fig. 3) detailed in Supplementary Notes 1 and 2 (Supplementary Figs. 1–5, Supplementary Tables 9–15), we annotated a total of 926,535 cCREs in the human genome (Supplementary Table 10) and 339,815 cCREs in the mouse genome (Supplementary Table 11), encoded by 7.9% and 3.4% of these genomes, respectively, with the smaller number of mouse cCREs resulting from the sparser biosample coverage of our mouse data sets. Partly because of a shift in data production in ENCODE phase III to focus on primary cells and tissues, the ENCODE III data increased the number of annotated human cCREs by 22% compared with ENCODE II and Roadmap data combined, with the increase being most evident for TSS-distal cCREs (Supplementary Note 3, Supplementary Fig. 6). The human registry of cCREs covers more than 80% of elements marked by H3K4me3 or H3K27ac or bound by CTCF (false discovery rate (FDR) <0.01) in any biosample and 50–70% of TSSs in the GENCODE and FANTOM collections (Supplementary Note 4, Supplementary Fig. 7). Whereas earlier studies identified putative enhancers on the basis of histone modification signatures, the ENCODE Registry is substantially larger both in the number of elements and in the range of biosamples surveyed (Supplementary Note 5, Supplementary Figs. 8, 9, Supplementary Table 16). Furthermore, the registry goes beyond cataloguing a list of elements by tracing the active or poised signature of each registry element across a large biosample space (Supplementary Note 1, Supplementary Figs. 1–5, Supplementary Tables 9–15). Analogously, whereas knowledge of well-annotated TSSs is sufficient to identify a substantial fraction of protein-coding and noncoding RNA promoter regions, we have enriched this information by annotating biosamples in which these promoters show evidence of activity or of being poised for activity. We note that our categories do not include elements with primary silencing activity, and we do not claim that the current cCRE classification scheme reflects the full biological spectrum of regulatory activities encoded in the genome.

### Classifying cCREs

We first partitioned cCREs into enhancer-like, promoter-like, and CTCF-only categories, noting that CTCF-occupied elements can specify several apparently different activities, including candidate insulators, enhancer blockers, and chromatin loop anchor elements^45,46^. Whereas a majority of enhancer-like elements map to promoter-distal regions (that is, more than a few kilobases from a TSS), many known enhancers lie in close proximity to a TSS^47^. Previously, ENCODE had analysed promoter-containing regions by using a generous fixed-interval definition (for example, ±2.5 kb around the TSS)^1^. That arbitrary cutoff had the effect of commingling the TSS and minimal-promoter function with promoter-proximal enhancer function. To better identify promoter-proximal enhancer-like cCREs and to help to distinguish them from active promoter signatures, we adopted a GENCODE TSS-aware approach that focuses on the dominant histone ChIP–seq signal, with additional parameters imposed around known TSSs (see Methods, Supplementary Note 1, Supplementary Figs. 1–5, Supplementary Tables 9–15). In this manner, we leveraged the high positional specificity of ENCODE DNase-seq data to more effectively use the histone modification patterns that have inherently lower resolution due to regional spreading around a TSS peak. This allowed us to define three major annotation groups: (i) active and poised enhancer-like elements (proximal and distal, 15.3% and 72.1% of human cCREs); (ii) active promoter-like elements (3.7% of human cCREs); and (iii) CTCF-only elements (6.1% of human cCREs), as explained in Box 1 and detailed in Supplementary Note 1. Elements in the three groups are referred to as having enhancer-like signatures (ELS), promoter-like signatures (PLS), or being CTCF-only, respectively. A fourth group contains likely poised elements marked by DNase and H3K4me3 (DNase–H3K4me3; 2.8% of human cCREs).

**Extended Data Fig. 1:**
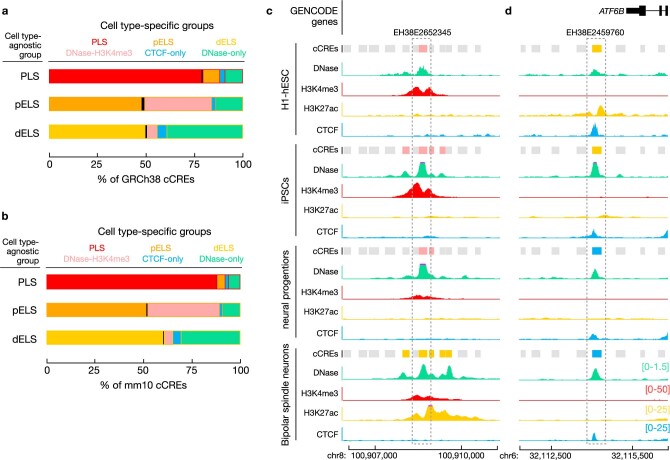
Classification of human cCREs is largely consistent across biosamples. **a**, **b**, For the 25 human (**a**) and 15 mouse (**b**) biosamples that were covered by all four core assays, we analysed how cCRE classification could differ between biosamples. For each cell-type-agnostic group of cCREs, the bars indicate their group classification in specific biosamples, coloured by group as indicated. Black indicates a switch in the grouping, for example, from cell type-agnostic PLS to cell type-specific pELS or CTCF-only. **c**, **d**, Two example switches of cCRE grouping between different biosamples. **c**, EH38E2652345 is a cCRE-dELS that has high DNase, H3K4me3, and H3K27ac signals in bipolar spindle neurons. By contrast, in cell types at earlier stages of neuronal differentiation, such as embryonic stem cells, iPSCs, and neural progenitor cells, this cCRE only has high DNase and H3K4me3 signals, suggesting that in these cell types the cCRE may be a poised enhancer. **d**, EH38E2459760 is a cCRE-dELS that has high DNase, H3K27ac, and CTCF signals in H1-hESCs and iPSCs. However, in further differentiated cell types such as neural progenitors and bipolar spindle neurons, the H3K27ac signal decreases while the CTCF signal remains, and accordingly, EH38E2459760 is classified as a CTCF-only cCRE. In **c** and **d**, cCRE colours correspond to group classification defined in **a** and **b**. Grey cCREs have low DNase signals.

This classification scheme, which we also applied to the mouse portion of the registry (Fig. 3), is intended to provide a useful high-level framework. However, the current cCRE classification scheme does not attempt to explicitly dissect complex multi-element modules. A notable subset (17%) of cCREs display complex or composite behaviours when examined across distinct biosamples, showing, for example, enhancer-like signatures in one cell type and a CTCF-only signature in another (Extended Data Fig. 1). These relationships can be readily extracted from the entire list of cCREs provided in Supplementary Tables 10, 11.

Box 1 Candidate *cis*-regulatory element classifications Groups based on function-associated signatures

A cCRE requires support from two distinct experimental assays: accessible DNA as measured by a high DNase signal and at least one high ChIP–seq signal (H3K4me3, H3K27ac, or CTCF) in the pertinent ChIP–seq dataset. The pertinent ChIP–seq dataset allows the cCREs to be classified into general groups. Specifically, we defined three major annotation groups using the following categorization schema for both human and mouse (Box 1 Fig. 1):

1) Active and poised enhancer-like elements: cCREs annotated with enhancer-like signatures (cCRE-ELS) have high DNase and H3K27ac signals and, if they fall within 2,000 bp of an annotated TSS, they must also have low relative H3K4me3 signal. We further partitioned ELSs into two subclasses on the basis of broader proximity to the TSS:

1a) Proximal enhancer-like elements: cCREs with proximal enhancer-like signatures (pELS) fall within 2 kb of a TSS.

1b) Distal enhancer-like elements: cCREs with distal enhancer-like signatures (dELS) fall more than 2 kb from the nearest TSS.

2) Active and poised promoter-like elements: cCREs annotated with promoter-like signatures (cCRE-PLS) possess high DNase signals and high H3K4me3 signals. They are partitioned into two subclasses on the basis of their proximity to a TSS.

2a) Canonical promoter-like elements (cCRE-PLS): these fall within 200 bp (centre-to-centre) of an annotated GENCODE TSS that has high DNase and H3K4me3 signals.

2b) Other high-H3K4me3 elements: cCREs with this annotation have high DNase with high H3K4me3 but low H3K27ac signals and do not fall within 200 bp of an annotated TSS. These elements may denote either poised canonical promoters, non-canonical promoter-like elements, or elements with other functions that lie within the high-H3K4me3 signal region around a canonical promoter.

3) CTCF-only elements: CTCF-only cCREs have high DNase and CTCF signals but low signals for H3K4me3 and H3K27ac. These isolated CTCF elements are candidates for insulators and looping functions in which CTCF participates. Other regulatory elements (ELS and PLS) can also be bound by CTCF, where this protein may also participate in those roles.

Tiers of data support

Placing cCREs into predicted functional groups on the basis of their epigenetic features ideally would be done with full knowledge of each feature in each biosample. However, as the breadth of biological systems expands, with a concomitant increase in the number of biosamples examined, it becomes very difficult to maintain full ascertainment of all features in all biosamples (Box 1 Fig. 2). The resulting gaps in knowledge complicate our assessment of function-associated signatures. To provide a guide for the completeness of the underlying data, we established the following tiers of cCRE function-related annotations. Specifically, cCREs are divided into tiers 1a, 1b, and 2 on the basis of their data support.

Tier 1a cCREs are fully defined, being supported by high DNase signal plus high H3K4me3, H3K27ac or CTCF signal within the same biosample and with all measurements complete in that biosample. These cCRE annotations are derived from the 25 human (and 15 mouse) biosamples with all four features determined (Box 1 Fig. 2).

Tier 1b cCREs are also supported by high DNase signal plus high H3K4me3, H3K27ac or CTCF, within the same biosample, although unlike tier 1a, they may lack some or all other data in that biosample.

Tier 2 cCREs are provisionally defined cCREs, given that the supporting data are available only in different biosamples. Tier 2 cCREs are supported by high DNase signal in one or more biosamples that lack data for the pertinent H3K4me3, H3K27ac, or CTCF features that were ultimately used to make the cCRE call. They are regarded as provisional because the pertinent histone mark or CTCF data came from a different biosample that lacked DNase data. Tier 2 cCREs can be promoted to tier 1 as additional pertinent data are determined within a single biosample, and this reclassification will be performed for each new build of the registry.

A detailed description of cCRE classifications into groups and tiers is in Supplementary Note 1.

**Figure:**
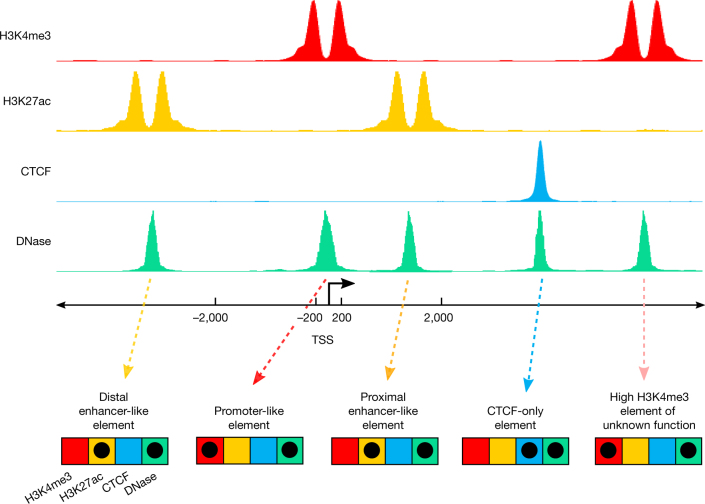
Box 1 Fig. 1 | Classification of cCREs by epigenetic signatures and proximity to TSS. The pertinent ChIP–seq data for each classification assignment is depicted as idealized signal tracks above the genomic-location scale focused on a transcription start site (TSS) of a GENCODE-annotated gene. A diagram depicting feature ascertainment (coloured boxes) and high signals (black dots) is shown below the scale.

**Figure:**
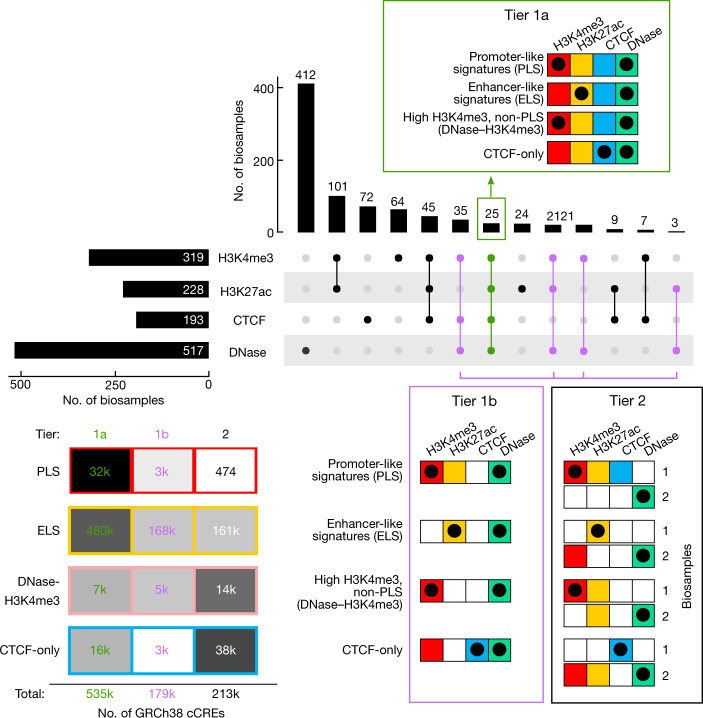
Box 1 Fig. 2 | Profiles of feature ascertainment across biosamples and confidence tiers for cCREs. Top, upset plot showing the numbers of biosamples with the set of feature determinations indicated below the plot. Group and tier assignments are shown by matrices of feature determination and an indication of whether a high signal was observed, using conventions defined in Box 1 Fig. 1. The matrix for tier 1a is within the upset plot, and those for tiers 1b and 2 are below the plot. Assessment of tier 2 requires examination of data for two biosamples, indicated to the right of the matrices. The heatmap in the lower left shows the numbers of cCREs in each group and tier.

### General properties of cCREs

**Extended Data Fig. 2:**
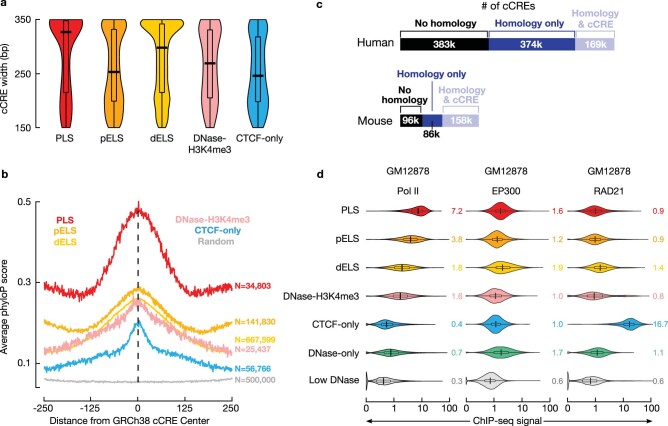
General properties of cCREs. **a**, Distributions of GRCh38 cCRE width in base pairs stratified by group classification. **b**, Average phyloP score in the ± 250 bp from the centre of each cCRE stratified by cell type-agnostic cCRE group: PLS (red), pELS (orange), dELS (yellow), DNase-H3K4me3 (pink), and CTCF-only (blue). In grey are 500,000 300-bp control regions randomly selected from mappable regions of the human genome. **c**, Fractions of human and mouse cCREs with homology in the other species. In black (no homology) are cCREs that do not map to the other genome. In dark blue (homology only) are cCREs that map to the other genome but do not overlap a cCRE in that genome. In light blue (homology & cCRE) are cCREs that map to cCREs in the other genome, which then reciprocally map back to the original genome. **d**, Transcription factor ChIP–seq signals support the group classification of cCREs. Violin plots show the average Pol II, EP300, and RAD21 ChIP–seq signals for cCREs belonging to each cCRE group, along with values indicating median signal levels. All ChIP–seq data and cCREs are in GM12878 cells. Colours of violins indicate cCRE groups (PLS, red, *N* = 17,119; pELS, orange, *N* = 29,435; dELS, yellow, *N* = 28,594; DNase-H3K4me3, pink, *N* = 7,298; CTCF-only, blue, *N* = 11,355; DNase-only, green, *N* = 9,394; low-DNase, grey, *N* = 823,340). Boxplots inside violins display median and first and third quartiles.

**Extended Data Fig. 3:**
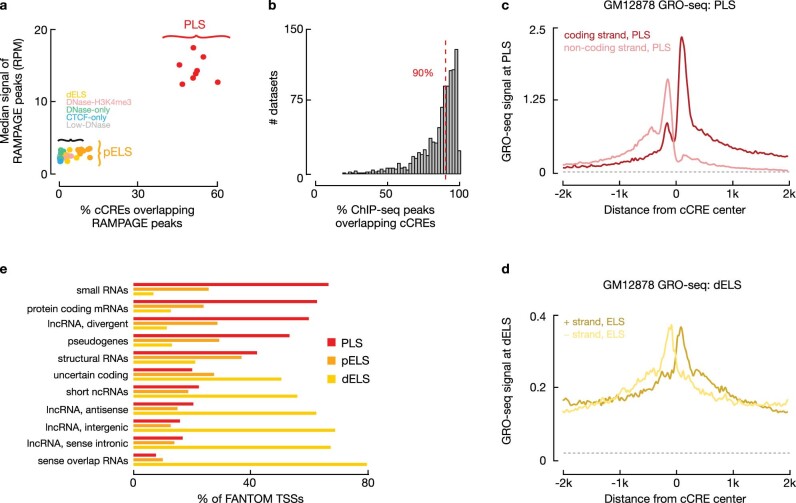
Summary of transcription and transcription factor binding at cCREs. **a**, Scatterplot depicting percent overlap of various groups of cCREs with RAMPAGE peaks in eight biosamples with matching data vs. the median expression level (in RPM) of the overlapping RAMPAGE peaks. **b**, The vast majority of high-quality ChIP–seq peaks of chromatin-associated proteins (mostly transcription factors) overlap cell type-agnostic cCREs. The median overlap is 90% across all ChIP–seq experiments. **c**, **d**, GRO-seq signal in GM12878 averaged over all cCRE-PLSs (**c**, in red) and cCRE-dELSs (**d**, in yellow) in a ± 2 kb window around cCRE centres. The GRO-seq signals around cCRE-PLSs were grouped by the orientation of their associated genes. The GRO-seq signals around cCRE-dELSs were grouped by genomic strands. Genomic background signal, computed as described in Supplementary Methods, is shown by the grey dashed lines and was approximately 0.02 for both strands in GM12878. **e**, Percentages of the transcription start sites of FANTOM CAGE-associated transcripts in the eleven FANTOM-defined categories that overlap cCRE-PLSs (red), cCRE-pELSs (orange), or cCRE-dELSs (yellow). The TSSs of the majority of coding-associated transcripts (protein-coding mRNA and divergent lncRNAs) overlapped a cCRE-PLS, while the TSSs of the majority of eRNA-like noncoding RNAs (short ncRNAs, antisense lncRNAs, intergenic lncRNAs, sense intronic lncRNAs, and sense overlap RNAs) overlapped a cCRE-dELS.

**Extended Data Fig. 4:**
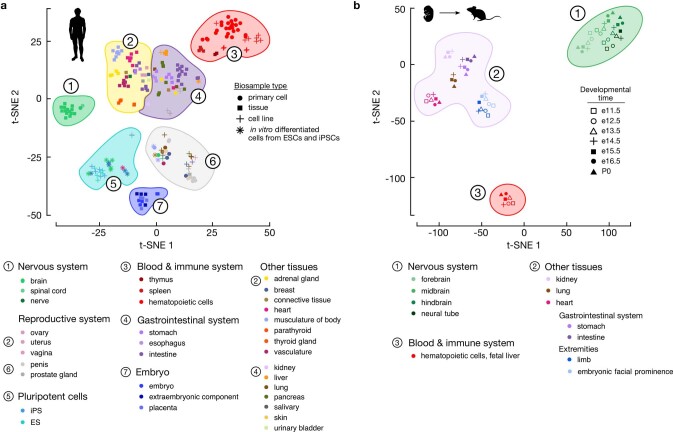
t-SNE analysis of human and mouse biosamples based on the H3K27ac signals at their cCREs. To investigate the relationship among biosamples and their tissues or cell types of origin, we performed t-SNE based on the H3K27ac signal at the cCRE-dELSs (human: 667,599 and mouse: 209,041) across all biosamples (human: 228 and mouse: 66). **a**, Human biosamples formed seven main clusters as determined by K-means clustering. Cluster 1 comprises adult brain tissues and embryonic neurospheres. Cluster 2 comprises tissues from the adrenal gland, heart, leg muscle, and muscular samples of the gastrointestinal (GI) system. Cluster 3 comprises haematopoietic cells and immune tissues including the spleen and thymus. Cluster 4 comprises tissue but those without strong muscle components such as kidney, liver, and mucosa of the gastrointestinal system. Cluster 5 comprises embryonic stem cells, induced pluripotent stem cells and in vitro differentiated cells from these pluripotent cell types. This cluster also includes two outliers, A673 and SK-N-MC cell lines. Cluster 6 comprises a mixture of cell lines and primary cells. Cluster 7 comprises tissues from embryonic structures such as the placenta and chorion. **b**, The mouse developmental tissue samples formed three large clusters: brain, liver (hepatic plus fetal haematopoietic systems), and other tissues, with related tissues cluster together, and several tissues (for example, the four brain regions, face, and limb) display a time-course dependent arrangement of the samples.

The distribution of cCREs along human chromosomes and the evolutionary conservation profiles of cCREs are similar to those of DHSs as a whole^48^ (Supplementary Note 6, Supplementary Fig. 10, Supplementary Table 17). Because cCREs are anchored on DHSs, they have relatively high resolution and range in size from 150 to 350 base pairs (bp; Extended Data Fig. 2a). Estimated levels of conservation were higher in all groups of cCREs than in randomly selected genomic regions, with the level of conservation decreasing from PLS to ELS to CTCF-only elements (Extended Data Fig. 2b; Supplementary Fig. 10a, b). A majority of the human (56%) and mouse (72%) cCREs had orthologous sequences in the other species, which was substantially higher than the background rates of 24% for human and 31% for mouse computed using randomly selected genomic regions with matched sizes. Furthermore, for a majority (65%) of mouse cCREs with human orthologues, the orthologue was also a cCRE (Extended Data Fig. 2c). cCRE categorizations were highly congruent with other ENCODE data types. For example, active cCRE-PLSs showed RNA polymerase II and RAMPAGE signals consistent with transcript initiation (Extended Data Figs. 2d, 3a). The cCRE-ELS elements showed occupancy by enhancer-associated co-activators such as EP300 (Extended Data Fig. 2d), and they overlapped significantly with experimentally determined enhancer elements in both human and mouse (see below). Consistent with an earlier study^48^, cCREs comprehensively overlapped the expanded range of ENCODE transcription factor ChIP–seq data; indeed, the median ENCODE transcription factor ChIP–seq dataset had 90% of peaks overlapping a cCRE (Extended Data Fig. 3b, Supplementary Note 7, Supplementary Fig. 11a–d, Supplementary Table 18). Furthermore, as expected for many active enhancers, most cCRE-ELSs showed nascent bidirectional transcription assayed by global run-on sequencing (GRO-seq) or precision nuclear run-on sequencing (PRO-seq) (Extended Data Fig. 3c, d, Supplementary Note 8, Supplementary Fig. 12), and cCRE-PLSs and cCRE-ELSs had high overlaps with specific classes of FANTOM-annotated TSSs and ChromHMM-annotated chromatin states (Extended Data Fig. 3e, Supplementary Notes 4, 5, Supplementary Figs. 8, 9, Supplementary Table 16). Overall, the activity landscape for cCRE-ELSs reflects tissue type, developmental origin, and developmental stage (Extended Data Fig. 4, Supplementary Table 19), and parallels the global organization of the expressed poly-A RNA transcriptome (Supplementary Note 9, Supplementary Fig. 11e–g, Supplementary Table 20). The mouse developmental series enables integration of differential gene expression with the differential epigenetic signals of nearby cCREs across multiple tissue types and aids the identification of cCREs that regulate gene expression programs (Supplementary Note 10, Supplementary Fig. 13, Supplementary Table 21).

### Experimental testing of cCRE function

To investigate the spatiotemporal activities of cCREs that were predicted to be enhancers in mid-gestation mouse embryos, we tested 151 cCRE-containing genomic segments using transgenic mouse enhancer–reporter assays (Supplementary Note 11, Supplementary Figs. 14, 15a–e, Supplementary Table 22). These segments were selected for testing on the basis of predicted cCRE activity in each of three mouse tissues (midbrain, hindbrain, limb) at a single developmental time point (post-conception embryonic day 11.5; E11.5). In brief, cCRE-containing segments were centred on DHSs present in the respective tissue followed by ranking according to the overlapping DNase and H3K27ac signal strengths in that tissue (see Methods). This resulted in three independently ranked lists of 104, 92, and 119 thousand DHSs with predicted enhancer function in mouse e11.5 midbrain, hindbrain, and limb, respectively. An initial transgenic reporter survey by ENCODE found that active constructs were concentrated in the top quartile of the H3K27ac signal (Supplementary Note 11). To explore this relationship further, we selected from three biochemical rank tiers: rank 1, those with the highest combined DNase and H3K27ac signal (~top 0.1%); rank 2, a group centred around rank 1,500; and rank 3, another group centred around rank 3,000. From each tissue-ranked group, we selected fragments with high signals for testing (51 fragments for midbrain, 50 for hindbrain, 50 for limb) (Supplementary Table 22).

**Fig. 4:**
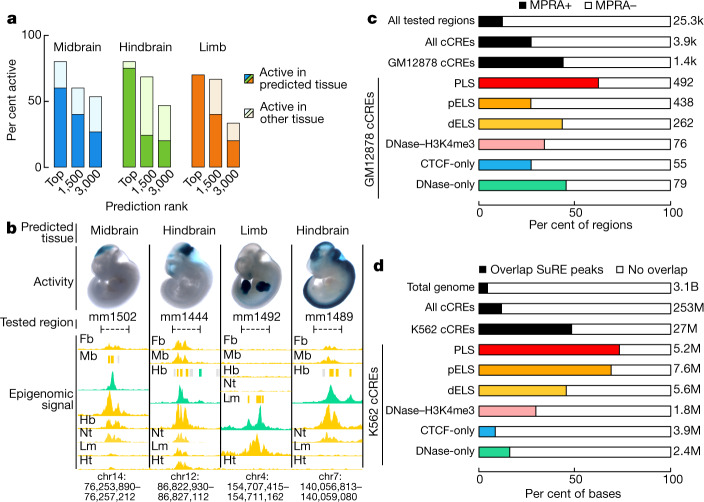
Experimental testing of cCRE activity in transgenic mouse assays and by comparison with public MPRA and SuRE data. **a**, The rates at which the 151 predicted enhancers (each centred on a cCRE-dELS) showed activity in transient transgenic mouse assays, stratified by their prediction ranks in each tissue. The lower, darker bars indicate that activity was detected in the predicted tissue, and the upper, lighter bars indicate that activity was detected in other tissues but not the predicted tissue. **b**, Four predicted enhancers that were shown to be active by transgenic mouse assay. Predicted enhancers (tested regions shown in dashed horizontal lines between vertical lines) and nearby cCREs (yellow, green, and grey boxes indicate cCRE-dELSs, DNase-only cCREs, and low-DNase cCREs, respectively, in the corresponding tissues) are depicted alongside DNase signal (green) and H3K27ac signal (yellow) in forebrain (Fb), midbrain (Mb), hindbrain (Hb), limb (Lm), and heart (Ht). Stained embryo images reveal the tissues in which each predicted enhancer tested as active. The two predicted hindbrain enhancers were active in additional brain regions (mm1444 in hindbrain and midbrain; mm1489 in hindbrain, midbrain, and neural tube). H3K27ac signal profiles across tissues accurately predicted additional observed activity in related tissues. Overall positive testing rates: mm1502, 3/3 embryos; mm1444, 7/9; mm1492, 5/5; mm1489, 5/5. **c**, Percentages of regions that tested positive or negative for enhancer activity by MPRA in lymphoblastoid cell lines (MPRA-positive, filled bars; MPRA-negative, white bars). The bars from top to bottom indicate all tested regions, only those tested regions overlapping cell type-agnostic cCREs, and only those tested regions overlapping cCREs identified in GM12878 cells, partitioned by cCRE group. **d**, Percentages of genomic positions tested by the Survey of Regulatory Elements (SuRE) assay for promoter activity in K562 cells (SuRE-positive, filled bars; SuRE-negative, white bars). The bars from top to bottom indicate all genomic positions (SuRE is a genome-wide assay), positions that overlap cell type-agnostic cCREs, and positions that overlap cCREs identified in K562 cells, partitioned by cCRE group.

Each of the 151 cCRE-containing segments was tested individually via a mouse transgenic enhancer–reporter assay that provided a sensitive spatial readout of reporter gene expression in whole embryos^49^. We performed multiple replicate assays (at least three independent transgenic embryos) for each segment. The cCRE-containing segments were judged to encode regulatory activity if *lacZ* expression was consistently and specifically observed in the target tissue at E11.5 (see Methods). Overall, 67 of the 151 tested cCREs showed detectable in vivo reporter activity that was consistent with its tissue prediction (Fig. 4a, b, Supplementary Note 11, Supplementary Fig. 15a–e). Moreover, the frequency of tissue-predicted in vivo activity in the transgenic assay declined as the composite H3K27ac-DNase score decreased, ranging from 60–75% for the highest-ranked cCRE-ELSs to 20–27% for those in the lowest ranks tested. As our cCRE-ELS lists were not filtered to exclude predicted activities in multiple tissues or to eliminate segments with more than one cCRE, nearly half of the constructs tested were active in other tissues in addition to the tissues used for selection and prioritization (Fig. 4b, Supplementary Figs. 14, 15a–e). In most cases, these cCRE-ELSs with activity across multiple tissues also had high composite H3K27ac–DNase scores in the corresponding active tissues; however, we also observed cCRE-ELSs with high scores across several tissues that tested positive in only a small subset of tissues (Supplementary Note 11). Highly similar overall results were obtained in a second transgenic study performed at E12.5 and reported in an ENCODE companion study^14^ (Supplementary Note 11, Supplementary Table 22).

We next compared cCREs with published results from two massively parallel reporter assays (MPRAs) conducted using the ENCODE reference human cell lines GM12878^50^ and K562^51^ (Supplementary Note 12, Supplementary Fig. 15f–h). Nearly half of ENCODE cCREs showed positive results in independent large-scale assays of enhancer and promoter activities. For cCREs defined in GM12878 that also overlapped with a set of independently selected MPRA elements^50^, 44% were active overall, whereas the background activity rate was 12%. Specifically, the proportions were 28.8%, 39.8% and 58% for proximal ELSs, distal ELSs, and PLSs, respectively (Fig. 4c, Supplementary Note 12, Supplementary Fig. 15f, g). Furthermore, when evaluated at the level of nucleotides, approximately 69%, 46%, and 73% of proximal ELSs, distal ELSs, and PLSs, respectively, defined in K562 showed positive results from the Survey of Regulatory Elements (SuRE) assay^51^ that had been designed to expose latent promoter functionality in the genome (Fig. 4d, Supplementary Note 12, Supplementary Fig. 15h). By contrast, the genome-wide background positive rate was only 4%. Thus, human cCREs were considerably enriched for enhancer-like activity despite the fact that the transient enhancer–reporter assays tested DNA fragments that were shorter than the average cCRE and frequently only partially overlapped the cCRE.

Overall, these initial functional assessments indicate that at least one-third of the cCRE-ELS compartment encodes transcriptional control elements that produce positive results in contemporary cell transfection assays, while a smaller number marked by stronger biochemical signatures are active in the more stringent transgenic mouse embryo system. However, it is important to acknowledge that each assay system has inherent limitations. None of the aforementioned methods interrogates cCREs in their native chromosomal context, nor do they test for combinatoric interactions among cCREs in *cis*. The assays also do not account for poised elements that exhibit DNase I hypersensitivity but are gated functionally by additional *trans*-acting signals or cell contexts. Furthermore, we acknowledge the possibility that not all open chromatin regions marked by high levels of H3K27ac function as enhancers; therefore, these regions will not test positive in the functional characterization experiments conducted here. These caveats are likely to be addressed in part by genome and epigenome editing approaches that enable in situ manipulation of regulatory DNA and associated chromatin.

### Accessing the registry

To facilitate access to the rich resource of DNA elements with likely positive transcriptional regulatory or insulator function encompassed within the Registry of cCREs, we created a web-based tool termed SCREEN (search candidate *cis*-regulatory elements by ENCODE; http://screen.encodeproject.org) (Box 2). SCREEN has three components (‘apps’): (i) a cCRE-focused application that enables the filtering, selection, and visualization of cCREs by biochemical signal or element category, and integration of cCREs with genes and ENCODE annotations such as transcription factor occupancy; (ii) a gene-expression-focused application that facilitates the retrieval of RNA transcription information for any biosamples with corresponding cCREs; and (iii) an application to facilitate the retrieval and integration of cCREs with human genetic variants from genome-wide association studies, as detailed in Supplementary Note 13 (Supplementary Figs. 16–20, Supplementary Table 23).

Box 2 Interactive use of cCREs via SCREEN A particularly powerful approach to using ENCODE data is to leverage the cCREs, gene expression and epigenetic data identified in both human tissues and cell lines and in multiple tissues during mouse fetal development. To facilitate analysis and visualization of cCREs and ENCODE data by the community, we have built a web-based resource called SCREEN (http://screen.encodeproject.org). SCREEN connects every cCRE with all available ENCODE epigenomic and transcriptomic data as well as external data from FANTOM and Cistrome (http://cistrome.org). A series of videos introducing and illustrating many of the capacities of SCREEN is available (links in the Supplementary Information).

SCREEN catalogues the 0.9 million human cCREs and 0.3 million mouse cCREs in the registry. Users can find cCREs of interest by searching for genes, genomic intervals, or GWAS phenotypes (Box 2 Fig. 1). Furthermore, SCREEN integrates cCREs with a wide range of annotations available at the ENCODE Portal, including gene and transcript expression profiles, chromatin accessible regions from DNase-seq, transcription factor and histone modification ChIP–seq peaks, and 3D chromatin interactions. Links and functionality are provided so that users can visualize data in the UCSC Genome Browser (https://genome.ucsc.edu/). Homologous cCREs between human and mouse are linked through SCREEN, facilitating evolutionary comparisons. To facilitate more extensive downstream analysis, all underlying data in SCREEN can be downloaded or accessed programmatically via an associated GraphQL application program interface (API).

SCREEN is organized into three ‘apps’—the cCRE app, the gene expression app, and the GWAS app—that provide different perspectives on the registry (Box 2 Fig. 1). Guided by biological questions, users can use the cCRE app to retrieve subsets of cCREs that meet search criteria and then select specific features or loci to visualize the underlying data. SCREEN’s Signal Profile tool displays DNase or histone modification signals at cCREs as ‘mini-peaks’ across biosamples. The gene app displays the expression levels for a specified gene and its individual transcripts as determined by RNA-seq and RAMPAGE in numerous cell and tissue types. Users can visualize differentially expressed genes alongside associated differential cCRE activity across mouse tissues and developmental time points. The GWAS (genome-wide association study) app annotates single-nucleotide polymorphisms (SNPs) from 3,751 published GWASs with cCREs (Supplementary Table 23), taking into account linkage disequilibrium (LD) between neighbouring genomic loci. Biosamples that are enriched for active cCREs that overlap GWAS SNPs have been identified for GWASs with sufficient SNPs to provide statistical power (that is, 25 or more SNPs), and these biosamples are preloaded into SCREEN. Supplementary Note 13 provides six detailed examples of how to use the registry and SCREEN to explore the annotations associated with GWAS SNPs.

**Figure:**
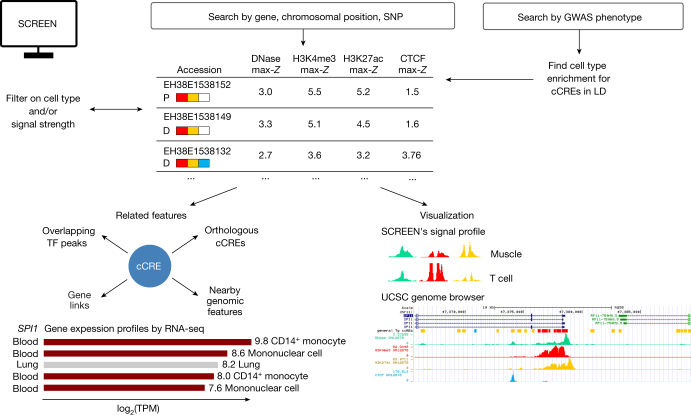
Box 2 Fig. 1 | The SCREEN resource provides multiple applications with which to interrogate cCREs, gene expression patterns, and GWAS variants.

### Other approaches using machine learning

In addition to the Registry of cCREs described in this report, one of the ENCODE companion papers developed a machine learning model that draws on the depth of ENCODE data in selected reference cell types to predict enhancers from self-transcribing active regulatory region sequencing (STARR-seq) data^52^. Another ENCODE companion paper expanded this model to connect cCREs with genes and thereby to construct large-scale regulatory networks that serve as a valuable resource for disease studies^38^. A two-dimensional, epigenetic state segmentation model, IDEAS^53^, served as the basis for regulatory region annotation and target gene assessments in mouse haematopoiesis^28^. In the developing mouse limb, IDEAS elements from bulk epigenomic data were deconvolved into specific cell type assignments by using single-cell RNA-seq^16^.

## Outlook

ENCODE element annotations aim to delineate specific segments of the human and mouse genomes that encode a potential biological function. We aim to predict the activities of ENCODE sequence elements within a given biological context or of the different combinations of elements that become active in different biological contexts. It has become apparent that, by virtually any metric, elements that govern transcription, chromatin organization, splicing, and other key aspects of genome control and function are densely encoded in many parts of the human genome sequence. However, most of these elements are actualized sparingly in a cell type- or state-selective manner, complicating assessment of the completeness of the ENCODE Encyclopedia, or what remains to be discovered. Functional elements that are active only in rare cell types are likely to be underrepresented in the current ENCODE Encyclopedia because many assays used heterogeneous whole tissue samples. Advances in single-cell genomics technologies may help to bridge these gaps by deconvolving in silico the epigenome or transcriptome profiles from a tissue sample into its constituent cell types^54,55^. However, the sensitivity of these approaches for detecting candidate functional elements compared with the assays we describe here performed on deeply sequenced bulk samples has yet to be determined.

Despite the very large number of biochemically defined elements within the ENCODE Encyclopedia, their functional annotation is currently limited to a few broad categories (enhancer, promoter, and insulator). Conventional assays of regulatory function, from transgenic mice to high-throughput reporter systems, have substantial technical and conceptual limitations, including their failure to capture combinatoric interactions of multiple *cis*-acting elements. Furthermore, the target genes for candidate distal enhancers in the registry have yet to be defined, which is currently among the on-going goals of ENCODE. It is anticipated that emerging functional genomic strategies involving genome or epigenome editing will provide considerable insights into the functional roles of biochemically marked elements.

Ultimately, we anticipate that the ENCODE Encyclopedia will help researchers to decode the molecular mechanisms that underpin the genetic bases of human traits and diseases^56^. The value of ENCODE-defined elements for interpreting genome-wide association studies was already apparent in earlier phases of the project and has improved in parallel with the expanding space of biological contexts sampled by ENCODE assays, strengthening the hypothesis that many noncoding risk variants function via transcriptional regulatory mechanisms^1,2^. We expect that a comprehensive catalogue of functional elements, with more precise and accurate functional annotations, such as cell type-specific usage, transcription factor binding, and regulatory target genes, will provide an even more powerful tool for realizing the translational potential of the human genome for the diagnosis and treatment of diverse diseases.

## Methods

### Ethical compliance

We have complied with all relevant ethical regulations regarding animal research and research involving humans. Each individual project that contributed data to ENCODE had their own institutional board that approved the study protocol.

### Biosample collection

All human biosamples were collected with open access consent that met relevant IRB standards. All mouse biosamples were approved by the respective institutional animal care and use committees. Details (for example, cell line sources, growth protocols, tissue harvesting, sex, age and so on) for individual biosamples are publicly available on the ENCODE portal. A representative example can be found here: https://www.encodeproject.org/biosamples/ENCBS689AWK/. Cell lines were not tested for mycoplasma contamination.

### RNA sequencing

#### Overview

In earlier phases of ENCODE, we surveyed transcriptome data mainly for immortalized cell lines using two approaches developed in Consortium laboratories—RNA-seq^57^ and CAGE^58^ (cap analysis of gene expression, providing a foundation for the GENCODE reference annotation of human genes and transcripts^59^). To survey transcriptomes across human and mouse biosamples, we performed a variety of RNA-seq experiments in ENCODE phase III (Table 1), which can be divided into three classes: (i) bulk RNA-seq surveys RNAs greater than 200 nt and comprises total RNA-seq, poly(A)^+^ RNA-seq, poly(A)^−^ RNA-seq, CRISPR RNA-seq, CRISPRi RNA-seq, shRNA-knockdown RNA-seq and siRNA-knockdown RNA-seq; (ii) small RNA-seq surveys RNAs less than 200 nt; and (iii) microRNA-seq surveys microRNA levels by selecting for species less than 30 nt. Additional assay details, along with detailed experimental protocols, are available at the ENCODE Portal^60^ (https://www.encodeproject.org/data-standards/rna-seq/long-rnas/, https://www.encodeproject.org/data-standards/rna-seq/small-rnas/ and https://www.encodeproject.org/microrna/microrna-seq/).

#### Uniform processing pipelines

There are two distinct ENCODE uniform RNA-seq pipelines, one for RNAs longer than 200 nt and the other for RNAs shorter than 200 nt. The long RNA pipeline is appropriate for processing libraries generated from mRNA, rRNA-depleted total RNA, or poly(A)^−^ RNA. The pipeline consumes RNA-seq reads in FASTQ format; alignment is performed with STAR, and gene and transcript quantifications are performed by RSEM against a gene annotation file, which contains by default GENCODE annotations. STAR also outputs normalized RNA-seq signals for both the + and − strands. Further details are available at https://github.com/ENCODE-DCC/long-rna-seq-pipeline.

#### Quality control

For all RNA-seq experiments, data quality is evaluated by calculating the number of aligned reads and replicate concordance.

### RAMPAGE

#### Overview

RAMPAGE captures 5′-complete cDNA to allow the identification and quantification of TSSs and transcript characterization. Production documents were generated for each experiment, and a representative experimental protocol is available at https://www.encodeproject.org/documents/0651efa6-7fd7-4b33-ab11-b05348c9f1c0/@@download/attachment/295491.pdf. Additional assay details are available at https://www.encodeproject.org/data-standards/rampage/.

#### Uniform processing pipeline

The ENCODE RAMPAGE pipeline is appropriate for libraries generated with RNAs longer than 200 nt, and it consumes reads in FASTQ format and produces alignments and normalized signals for both the + and − strands with STAR. Peaks, representing TSSs, are called from the alignments using GRIT, and output in BED, bigBED, and GFF formats. Quality control (QC) is performed for the peaks, and the irreproducible discovery rate (IDR) is used to identify reproducible peaks between replicates.

#### Quality control

Data quality is evaluated by calculating read depth and replicate concordance.

### eCLIP

#### Overview

Enhanced crosslinking and immunoprecipitation (eCLIP) identifies transcriptome wide RBP occupancy sites^23^. By modifying steps in CLIP-seq and iCLIP protocols, eCLIP requires fewer amplification cycles and results in fewer redundant reads. Additionally, with the eCLIP protocol, size-matched inputs are generated to serve as controls for peak calling and other downstream analyses. The experimental protocol is available at https://www.encodeproject.org/documents/842f7424-5396-424a-a1a3-3f18707c3222/@@download/attachment/eCLIP_SOP_v1.P_110915.pdf.

Additional assay details are available at https://www.encodeproject.org/eclip/.

#### Antibody characterization

We require all eCLIP antibodies to undergo primary and secondary characterizations. Detailed RBP antibody standards are available at https://www.encodeproject.org/documents/fb70e2e7-8a2d-425b-b2a0-9c39fa296816/@@download/attachment/ENCODE_Approved_Nov_2016_RBP_Antibody_Characterization_Guidelines.pdf.

#### Processing pipeline

Data were processed by the Yeo laboratory using their eCLIP pipeline. In brief, adaptor trimmed reads were mapped to the human genome using STAR, and redundant reads were removed. Peaks were called using CLIPper. The pipeline is available at https://github.com/gpratt/gatk/releases/tag/2.3.2.

The pipeline description is available at https://www.encodeproject.org/documents/3b1b2762-269a-4978-902e-0e1f91615782/@@download/attachment/eCLIP_analysisSOP_v2.0.pdf.

#### Quality control

Data quality is evaluated by calculating the number of unique fragments, IDR, and the fraction of reads in peaks (FRiP).

### RNA Bind-n-Seq

#### Overview

RNA Bind-n-Seq characterizes RBPs and their motifs in vitro^61^. Recombinant RBPs are purified and incubated with randomized RNAs. The RBPs are then captured, and bound RNAs are sequenced. The experimental protocol is available at https://www.encodeproject.org/documents/aa71cabf-aaee-4358-a834-c6ee002938b8/@@download/attachment/RBNSExperimentalProtocol_Feb2016_96well.pdf.

Additional assay details are available at https://www.encodeproject.org/rbns/.

#### Processing pipeline

Bind-n-Seq data were processed by the Burge laboratory. In brief, reads were separated into ‘input’ and ‘pull-down’ groups. *K*mer enrichment was calculated by comparing the frequency of *k*mers in the pull-down groups to those in the input groups. The estimated binding fraction was calculated using streaming *k*mer analysis. Motif logos were created by aligning enriched *k*mers that met specific threshold criteria. The pipeline is available at https://bitbucket.org/pfreese/rbns_pipeline/src/master/.

The pipeline description is available at https://www.encodeproject.org/documents/c8b3442a-7e63-4847-af11-c72597bf65b3/@@download/attachment/RBNS_Computational_Pipeline_Aug_2016_update_Dec2018.pdf.

#### Quality control

Data quality is evaluated by calculating the number of recovered reads per concentration, *k*mer enrichments, and the Coomassie gel size and purity test of the recombinant protein.

### Histone ChIP–seq

#### Overview

Histone ChIP–seq surveys the interaction between DNA and histone proteins, selecting for a specific protein variant or post-translational modification through immunoprecipitation followed by sequencing. We also profiled a panel of 22 proteins involved in the deposition or recognition of histone modifications^62^, many of which have been implicated in developmental disorders and cancer progression^63^. The experimental protocols are available at https://www.encodeproject.org/documents/be2a0f12-af38-430c-8f2d-57953baab5f5/@@download/attachment/Epigenomics_Alternative_Mag_Bead_ChIP_Protocol_v1.1_exp.pdf (Bernstein laboratory, human) and https://www.encodeproject.org/documents/18580e80-0907-4258-a412-46bcc37bd040/@@download/attachment/Ren%20Lab%20ENCODE%20Chromatin%20Immunoprecipitation%20Protocol%20MicroChIP.pdf (Ren laboratory, mouse). Additional assay details are available at https://www.encodeproject.org/chip-seq/histone/.

#### Antibody characterization

We required all commercial histone antibodies to be validated by at least two independent methods, and antibody lots to be analysed independently. Detailed histone mark antibody standards are available at https://www.encodeproject.org/documents/4bb40778-387a-47c4-ab24-cebe64ead5ae/@@download/attachment/ENCODE_Approved_Oct_2016_Histone_and_Chromatin_associated_Proteins_Antibody_Characterization_Guidelines.pdf.

#### Uniform processing pipeline

The ENCODE consortium histone ChIP–seq data pipeline takes into account the different binding distributions of the respective immunoprecipitation targets across the genome. The ChIP–seq pipelines consume raw reads in FASTQ format; alignment of the reads is performed with BWA to generate alignment BAMs. Signal tracks are produced from the alignments using MACS2; these are output in two separate bigWigs, which represent fold-change over control and signal *P* value. Peaks are also called from the alignments, using MACS2. Additionally, the pipeline calls peaks from the pooled alignments of each experiment’s isogenic replicates. Sets of replicated histone mark peaks are generated by comparing the pooled and individual peaks using overlap_peaks. Further detail and basic workflows are available at https://github.com/ENCODE-DCC/chip-seq-pipeline.

#### Quality control

Data quality is evaluated by calculating read depth, non-redundant fraction (NRF) (that is, the number of distinctly uniquely mapping reads over the total number of reads), and PCR bottlenecking coefficients (PBC1 and PBC2).

### ChIP–seq of chromatin-associated proteins

#### Overview

ChIP–seq surveys the interaction between DNA and DNA regulatory proteins such as transcription factors and chromatin remodellers through immunoprecipitation followed by sequencing. The experimental protocol is available at https://www.encodeproject.org/documents/20ebf60b-4009-4a57-a540-8fd93407eccc/@@download/attachment/Epigenomics_CR_ChIP_Protocol_v1.0.pdf (Bernstein laboratory), https://www.encodeproject.org/documents/6ecd8240-a351-479b-9de6-f09ca3702ac3/@@download/attachment/ChIP-seq_Protocol_v011014.pdf and https://www.encodeproject.org/documents/a59e54bc-ec64-4401-8cf6-b60161e1eae9/@@download/attachment/EN-TEx%20ChIP-seq%20Protocol%20-%20Myers%20Lab.pdf (Myers laboratory), and https://www.encodeproject.org/documents/f2aa60f2-90a6-4e4b-863a-c6831be371a2/@@download/attachment/ChIP-Seq%20Biorupter%20Pico%20TruSeq%20protocol%20for%20Syapse-c5bdc444fe0511e69d6a06346f39f379.pdf (Snyder laboratory). Additional assay details are available at https://www.encodeproject.org/chip-seq/transcription_factor/.

#### Antibody characterization

We required antibodies to undergo primary and secondary characterizations for each lot. For epitope-tagged proteins, we developed a protocol that includes genomic DNA characterization followed by immuno-characterization. Additional details are available at https://www.encodeproject.org/documents/c7cb0632-7e5f-455e-9119-46a54f160711/@@download/attachment/ENCODE_Approved_May_2016_TF_Antibody%20Characterization_Guidelines.pdf (TF antibodies) and https://www.encodeproject.org/documents/35a9f776-dd6a-44e3-8795-50ead83f34f7/@@download/attachment/Guidelines_for_Use_of_Epitope_Tags_in_ChIP-seq_Jan_2017.pdf (epitope-tagged proteins).

#### Uniform processing pipeline

The ENCODE consortium has developed a TF ChIP–seq data pipeline that takes into account the different binding distributions of the respective immunoprecipitation targets across the genome. The ChIP–seq pipelines consume raw reads in FASTQ format; alignment of the reads is performed with BWA to generate alignment BAMs. Signal tracks are produced from the alignments using MACS2; these are output in two separate bigWigs, which represent fold-change over control and signal *P* value. Peaks are also called from the alignments using SPP. Additionally, the pipelines call peaks from the pooled alignments of each experiment’s isogenic replicates. For TF experiments, the pooled peaks are compared with the peaks called for each replicate individually using IDR and thresholded to generate a conservative set of peaks and an optimal set of peaks. Further detail and basic workflows are available at https://github.com/ENCODE-DCC/chip-seq-pipeline.

#### Quality control

Data quality is evaluated by calculating read depth, NRF, PCR bottlenecking coefficients (PBC1 and PBC2), replicate concordance using IDR, and FRiP.

### ATAC–seq

#### Overview

ATAC–seq surveys open chromatin regions through the insertion of primers into the genome via transposase followed by sequencing^27^. Experimental protocols are available at https://www.encodeproject.org/documents/404ab3a6-4766-45ca-af80-878a344f07b6/@@download/attachment/ATAC-Seq%20protocol.pdf (Snyder laboratory, human) and https://www.encodeproject.org/documents/4a2fc974-f021-4f85-ba7a-bd401fe682d1/@@download/attachment/RenLab_ATACseq_protocol_20170130.pdf (Ren laboratory, mouse). Additional details can be found at https://www.encodeproject.org/atac-seq/.

#### Processing pipeline

Experiments were processed using the Kundaje laboratory’s ATAC–seq pipeline (https://github.com/ENCODE-DCC/atac-seq-pipeline). In brief, trimmed reads were aligned to the genome using Bowtie2. Signal files and peak calls were generated using MACS. The pipeline also calls peaks from the pooled alignments of each experiment’s replicates. The pooled peaks were compared with the peaks called for each replicate individually using IDR and thresholded to generate a conservative set of peaks and an optimal set of peaks. In the near future, this pipeline will be incorporated as one of the ENCODE uniform processing pipelines.

#### Quality control

Data quality is evaluated by calculating the number of non-duplicate, non-mitochondrial aligned reads, alignment rate, IDR, NRF, PCR bottlenecking coefficients (PBC1 and PBC2), number of resulting peaks, fragment length distribution, FRiP, and TSS enrichment.

### DNase-seq

#### Overview

DNase-seq surveys open chromatin regions through genomic cleavage by endonuclease DNase I followed by sequencing. For ENCODE phase III, the DNase-seq protocol was updated, allowing for smaller quantities of input material. Experimental protocols are available at https://www.encodeproject.org/documents/926174f5-d14c-4e77-bc52-5517b56daac0/@@download/attachment/Culturedcells_SOP_nuclei_DNase_crosslink_RNA_V1.pdf (cultured cells) and https://www.encodeproject.org/documents/c6ceebb6-9a7a-4277-b7be-4a3c1ce1cfc6/@@download/attachment/08112010_nuclei_isolation_human__tissue_V6_3.pdf (tissues). Additional details are available at https://www.encodeproject.org/data-standards/dnase-seq/.

#### Uniform processing pipeline

The ENCODE DNase-seq processing pipeline consumes raw sequencing reads from technical replicates of experiments in the form of FASTQ files. Indexing and alignment of the FASTQ reads is performed with the Burrows–Wheeler Aligner (BWA^64^), which outputs alignments in BAM format. Alignments from sets of technical replicates are merged and filtered before peak calling with HOTSPOT2, which generates peaks in BED format. Input FASTQs must meet minimum criteria to be processed, and various quality control metrics are also generated at each step. Further detail and basic workflows are available at https://github.com/ENCODE-DCC/dnase_pipeline.

#### Quality control

Data quality is evaluated by calculating the number of uniquely mapping reads, the fraction of mitochondrial reads, and the signal portion of tags (SPOT) score.

### WGBS

#### Overview

To map DNA methylation, WGBS uses bisulfite treatment to convert unmethylated cytosines into uracils, leaving methylated cytosines unchanged. Through sequencing and alignment to a transformed genome, CpG, CHG, and CHH methylation levels can be extracted. The experimental protocol is available at https://www.encodeproject.org/documents/9d9cbba0-5ebe-482b-9fa3-d93a968a7045/@@download/attachment/WGBS_V4_protocol.pdf (human) and https://www.encodeproject.org/documents/8f3cbe33-cf8f-4f26-b76b-d14a3b9721bd/@@download/attachment/Ecker_Methyl_Protocol_022315.pdf (mouse). Additional details are available at https://www.encodeproject.org/data-standards/wgbs/.

#### Uniform processing pipeline

ENCODE WGBS pipelines are available for paired-end and single-end data. In summary, the pipeline maps trimmed reads to a Bismark-transformed genome using Bowtie2. Methylation states at CpGs, CHHs, and CHGs are quantified using Bismark and custom python scripts. Pearson correlation of CpG methylation is then calculated between replicates. Further detail and basic workflows are available at https://github.com/ENCODE-DCC/dna-me-pipeline.

#### Quality control

Data quality is evaluated by genomic coverage, C-to-T conversion rate, and correlation of CpG methylation levels between replicates.

### DNAme array

#### Summary

DNAme arrays measure methylation at CpGs. Like WGBS, DNA is treated with bisulfite to convert unmethylated cytosines to uracils. After amplification, DNA is hybridized to an array (Illumina Infinium Methylation EPIC BeadChip) with probes for both methylated and unmethylated states. Methylation is then quantified by comparing the signal between the two probes. All ENCODE uniform processing pipelines can be found at https://github.com/ENCODE-DCC.

### DNA replication timing

#### Overview

DNA replication timing provides insights into both gene regulation and spatiotemporal genome compartmentalization^65^. Production documents were generated for each experiment, and a representative experimental protocol for Repli-seq is available at: https://www.encodeproject.org/documents/59c9ceae-9f55-41c1-b5ce-78dc7bd59a1e/@@download/attachment/Repliseq_Protocol.pdf.

A representative experimental protocol for Repli-chip is available at: https://www.encodeproject.org/documents/97c4a9b3-8037-4fa4-a348-f396fcc3ecd1/@@download/attachment/wgEncodeFsuRepliChip.release2.html.pdf.

#### Processing pipeline

Repli-chip data were processed using LIMMA^66^. Repli-seq data were mapped to the hg19 genome using Bowtie2^67^. Details are available at: https://www.encodeproject.org/pipelines/ENCPL734EDH/.

### Metadata

The ENCODE Data Coordination Center (DCC), in collaboration with the laboratories performing the assays and the Data Analysis Center (DAC), has defined a set of metadata to describe the experimental conditions that were used to generate the data, processing steps that were performed to analyse and interpret the data, and metrics to evaluate the quality and reproducibility of the data (https://www.encodeproject.org/help/data-organization/). Metadata describe experimental assays, biosamples, antibodies, computational analysis. Metadata are organized as JSON objects and can be queried programmatically using a REST API. In order to ensure metadata accuracy, each schema has a set of dependencies to enforce proper modelling when related metadata are submitted. After submission, a system of audits is used to identify inconsistencies in the data. These audits are also used to communicate details of ENCODE data, such as data quality relative to standards, to the public^68^. Each audit is designated a colour depending on its severity and is displayed on the search page and individual object pages. The metadata contain the protocol, date created, lab, and sequencing platform, which can be used for removing batch effects during integrative analysis.

### The Registry of cCREs in human and mouse

The scripts for generating the Registries of cCREs and subsequent analyses are available in a GitHub repository (https://github.com/weng-lab/ENCODE-cCREs/), with details provided in Supplementary Methods.

### Identifying rDHSs

We used all DNase-seq data sets with SPOT scores of more than 0.3 on the ENCODE portal as of 1 September 2018 (Supplementary Table 9c, h). We called DNase peaks using iterative FDR thresholds to account for different sequencing depths among DNase-seq data sets (see Supplementary Methods). Peaks were then filtered on the basis of signal (over tenth percentile defined using all DNase-seq data sets), width (within 150–350 bp), and FDR (under 1 × 10^−3^). DNase peaks were clustered across all DNase-seq experiments, and we selected the peak with the highest signal (normalized by sequencing depth) in each cluster as the representative DNase hypersensitive sites (rDHS) for the cluster. All the DNase peaks that overlapped this rDHS by at least one base pair were considered represented by the rDHS and removed for subsequent iterations. We updated the clusters, identified the next rDHS with the highest signal, and removed all the DHSs that it represented. This process was repeated until it finally resulted in a list of non-overlapping rDHSs that represented all DNase peaks. To reduce the number of false positives, we discarded the rDHSs that did not overlap a collection of consensus DHSs (cDHSs), independently derived by taking a consensus across the DHSs across multiple samples^11^; this cDHS filtering process eliminated 3% of the rDHSs (see Supplementary Methods).

### Normalizing epigenomic signals at rDHSs

For each rDHS, we computed the *Z*-scores of the log_10_ of DNase, H3K4me3, H3K27ac, and CTCF signals in each biosample with such data. *Z*-score computation is necessary for the signals to be comparable across biosamples because the uniform processing pipelines for DNase-seq and ChIP–seq data produce different types of signals. The DNase-seq signal is in sequencing-depth-normalized read counts, whereas the ChIP–seq signal is the fold change of ChIP over input. Even for the ChIP–seq signal, which is normalized using a control experiment, substantial variation remains in the range of signals among biosamples. To illustrate this phenomenon, we examined the distributions of H3K27ac signals for 100,000 randomly selected rDHSs across five different biosamples—even though these data sets were processed uniformly by the same pipeline, the ranges and distributions of signals differ among the data sets (Supplementary Fig. 21a). The log_10_ of the signal in each biosample roughly follows a normal distribution (Supplementary Fig. 21b). The *Z*-scores of log_10_(signal) have the same distributions across biosamples (Supplementary Fig. 21c).

To implement this *Z*-score normalization, we used the UCSC tool bigWigAverageOverBed to compute the signal for each rDHS for a DNase, H3K4me3, H3K27ac, or CTCF experiment. For DNase and CTCF, the signal was averaged across the genomic positions in the rDHS. The signals of H3K4me3 and H3K27ac were averaged across an extended region—the rDHS plus a 500-bp flanking region on each side—to account for these histone marks at the flanking nucleosomes. Using a custom Python script, we took the log_10_ of these signals and computed a *Z*-score for each rDHS compared with all other rDHSs within a biosample. rDHSs with a raw signal of 0 were assigned a *Z*-score of −10.

### Identifying and classifying cCREs

Using the scheme outlined above, we calculated the *Z*-scores of the log_10_(signal) for the 2.2 million human and 1.2 million mouse rDHSs in each species for each experiment of the four core assays—DNase-seq and H3K4me3, H3K27ac, and CTCF ChIP–seq. For each rDHS, we then determined the maximum *Z*-score (max-*Z*) for each of the four core assays across all biosamples. The rDHSs with a high DNase max-*Z* and another high max-*Z* for at least one of the other three ChIP–seq marks were defined as cCREs. A *Z*-score cutoff of 1.64 corresponds to the 95th percentile for a one-sided Gaussian distribution. A high *Z*-score or a max-*Z* value is defined as >1.64 throughout, and low otherwise.

Considering the max-*Z* values across all biosamples but not the *Z*-scores in a specific biosample, cCREs were classified into seven states and five groups. A state stands for a specific high–low combination of a cCRE’s H3K4me3, H3K27ac, or CTCF max-*Z* values; seven states are possible because at least one mark needs to have a high signal. For the group classification, we further took into account the genomic distance from the centre of the cCRE to the nearest TSS (≤200 bp for TSS-overlapping, 200–2,000 bp for TSS-proximal, and >2,000 bp for TSS-distal). We defined TSSs as the 5′ ends of all basic transcripts annotated by GENCODE (V24 for human and M18 for mouse). A cCRE was assigned to one of five mutually exclusive groups on the basis of its state and TSS proximity (Box 1): TSS-overlapping with promoter-like signatures (PLS), TSS-proximal with enhancer-like signatures (pELS), TSS-distal with enhancer-like signatures (dELS), not TSS-overlapping and with high DNase and H3K4me3 signals only (DNase–H3K4me3), not TSS-overlapping and with high DNase and CTCF signals only (CTCF-only). Note that this set of seven states and five groups is defined across all biosamples, and therefore is cell-type agnostic. We next define cell type-specific state and group classifications.

To classify cCREs in a particular biosample covered by all four core assays, we used DNase, H3K4me3, H3K27ac, or CTCF *Z*-scores in that particular biosample. We had all four types of data for 25 human and 15 mouse biosamples. The cCREs in each of these biosamples were assigned to one of nine states—one low-DNase state regardless of H3K4me3, H3K27ac, and CTCF *Z*-scores, and eight high-DNase states with the high–low combinations of their H3K4me3, H3K27ac, and CTCF *Z*-scores. These eight high-DNase states were again combined with the distance from the nearest TSS to yield six mutually exclusive groups—PLS, pELS, dELS, DNase–H3K4me3, CTCF-only, and DNase-only, according to the classification diagram (Supplementary Fig. 2). The low-DNase state is included as the seventh group. Thus, in a particular biosample fully covered by all four core assays, cCREs were classified into nine states and seven groups.

Biosamples that are not fully covered by all four assays can also be used to define cCREs. To distinguish a low signal for a mark from missing data for that mark (that is, the assay was not performed for that mark in the biosample), we assign a confidence tier to each cCRE based on its supporting data (Box 1). Tier 1 cCREs are supported by a high DNase signal plus minimally one more high-signal mark in the same biosample; that is, these two high signals are concordantly observed in the same sample. Tier 1 cCREs were further separated into sub-tiers 1a and 1b, depending on whether the biosample that had high signals for this cCRE was fully covered by the four core assays (Box 1). Thus, all tier 1a cCREs are from the 25 human and 15 mouse biosamples that are fully covered by the four core assays, whereas tier 1b cCREs are from biosamples not fully covered by the four core assays. Tier 2 cCREs are supported by a high DNase signal in one biosample and a high signal for one more mark in a different biosample, but the concordance test could not be performed for the tier 2 cCREs owing to missing pertinent data for the cell type-agnostic classification of the cCRE. For example, for a tier 2 cCRE with a cell type-agnostic group classification of PLS, none of the biosamples with a high DNase signal at this cCRE had available H3K4me3 ChIP–seq data, and none of the biosamples with a high H3K4me3 signal at this cCRE had available DNase-seq data. There are also tier 3 and tier 4 cCREs, which were excluded from the current versions of the registries (see Supplementary Methods for details).

We also attempted to make group assignments for cCREs in a particular biosample that was not fully covered by the four core assays, making some approximations. The specific schemes are illustrated in Supplementary Fig. 3 and summarized as follows. For samples with DNase data, we classified elements using the available marks. For example, if a sample lacked H3K27ac (Supplementary Fig. 3e) its cCREs was assigned to the PLS and DNase–H3K4me3 groups but not the pELS or dELS groups. For biosamples lacking DNase data, we do not have the resolution to identify specific elements (Supplementary Fig. 3f). Therefore, for these biosamples, we simply labelled the cCRE as having a high or low signal for every available assay. In these biosamples, cCREs with low H3K4me3, H3K27ac, or CTCF signals were labelled ‘unclassified’ because we were unable to classify them as low-DNase without DNase data. In both SCREEN and in downloadable files, biosamples lacking data are clearly labelled as such.

For average conservation score analysis on each set of cCREs (Extended Data Fig. 2b), we calculated the average phyloP^69^ score (calculated from the alignment of 100 vertebrate genomes http://hgdownload.cse.ucsc.edu/goldenpath/hg38/phyloP100way/hg38.phyloP100way.bw) per base, ±250 bp from the centre of each cCRE. Homologous human and mouse cCREs were identified by liftOver^70^ with a minimum match score of 0.5 (Extended Data Fig. 2c).

### Test cCREs with transgenic mouse assays

We selected regions containing cCRE-dELSs in three E11.5 mouse tissues (midbrain, hindbrain, and limb) for testing using E11.5 transgenic mouse assays. We excluded dELS-containing regions that overlapped any previously tested regions that were already in the VISTA database (http://enhancer.lbl.gov/). We ranked dELS-containing regions from the most to the least significant by the average rank of DNase and H3K27ac signals in the corresponding tissue and then selected regions from three segments of each tissue’s ranked list (the top, around 1,500, and around 3,000 by rank). We used H3K27ac peaks (called using the ENCODE uniform processing pipeline) that overlapped the cCRE-dELSs to choose the boundaries of the tested regions. In total, we tested 151 regions across the three tissues (Supplementary Table 22).

Transgenic mouse assays were performed in FVB/NCrl strain *M. musculus* animals (Charles River) as described previously^49^. In brief, predicted enhancers were PCR amplified and cloned into a plasmid upstream of a minimal *Hsp68* promoter and a *lacZ* reporter gene. The plasmids were pronuclear injected into fertilized mouse eggs, and the transgenic embryos were implanted into surrogate mothers, collected at E11.5, and stained for β-galactosidase activity. A predicted element was scored positive as an enhancer if at least three embryos had identical β-galactosidase staining in the same tissue. Conversely, a prediction was deemed inactive if no reproducible staining was observed and at least five embryos harbouring a transgene insertion were obtained.

### Evaluating cCREs using public MPRA data

We downloaded the SNPs tested by MPRA^50^ in human lymphoblastoid cells from Supplementary Table 1 of that study and reconstructed tested regions by generating a ±75-bp window around each SNP. We then intersected cCREs with these regions using bedtools intersect, requiring at least 25% of each cCRE to overlap. Of the cCREs that overlapped a tested region, we calculated the percentage that overlapped an MPRA^+^ region. We analysed all cCREs and GM12878-specific cCREs stratified by the cCRE group.

### Evaluating cCREs with public SuRE data

We downloaded SuRE peaks in human K562 cells from the Supplementary Data Set of an earlier study^51^. Using bedtools intersect, we compared the SuRE peaks with the hg38 cCREs lifted down to the hg19 genome version, counting the number of base pairs overlapping each cCRE or region of interest. We then calculated the total percentage of base pairs for each cCRE group that overlapped a SuRE peak.

### Reporting summary

Further information on research design is available in the Nature Research Reporting Summary linked to this paper.

## Online content

Any methods, additional references, Nature Research reporting summaries, source data, extended data, supplementary information, acknowledgements, peer review information; details of author contributions and competing interests; and statements of data and code availability are available at 10.1038/s41586-020-2493-4.

## Supplementary information

Supplementary Information This file contains Supplementary Notes 1-13, Supplementary Methods, Supplementary References, The ENCODE Project Consortium list, and Supplementary Figures 1-21.

Reporting Summary

Supplementary Table 1 | ENCODE phase III transcriptome data.

Supplementary Table 2 | ENCODE phase III RBP experiments.

Supplementary Table 3 | ENCODE phase III ChIP-seq of DNA associated proteins.

Supplementary Table 4 | ENCODE phase III chromatin accessibility experiments.

Supplementary Table 5 | ENCODE phase III histone ChIP-seq experiments.

Supplementary Table 6 | ENCODE phase III DNA methylation experiments.

Supplementary Table 7 | ENCODE phase III 3D chromatin experiments.

Supplementary Table 8 | ENCODE phase III DNA replication experiments.

Supplementary Table 9 | Input datasets for building the Registry of cCREs.

Supplementary Table 10 | Human GRCh38 cCREs.

Supplementary Table 11 | Mouse mm10 cCREs.

Supplementary Table 12 | VISTA regions used for evaluating epigenetic signals.

Supplementary Table 13 | The performance (AUPR) of using epigenetic signals to predict VISTA enhancers.

Supplementary Table 14 | Using epigenetic signals to predict transcript expression.

Supplementary Table 15 | Relative abundance of cCRE-PLSs vs. cCRE-ELSs in 25 human biosamples with full assay coverage.

Supplementary Table 16 | Regions and genomic positions of ChromHMM promoter and enhancer states that overlap cCREs 5.

Supplementary Table 17 | Transposon and repeat content of cCREs.

Supplementary Table 18 | Overlap of TF ChIP-seq peaks with cCREs.

Supplementary Table 19 | t-SNE clusters from Extended Data Fig. 4.

Supplementary Table 20 | cCREs near tissue-specific genes.

Supplementary Table 21 | RNA-seq experiments included in SCREEN's differential expression tool.

Supplementary Table 22 | Testing candidate enhancers with mouse transgenic assays.

Supplementary Table 23 | Genome-wide association studies.

## Figures and Tables

**Extended Data Table 1:**
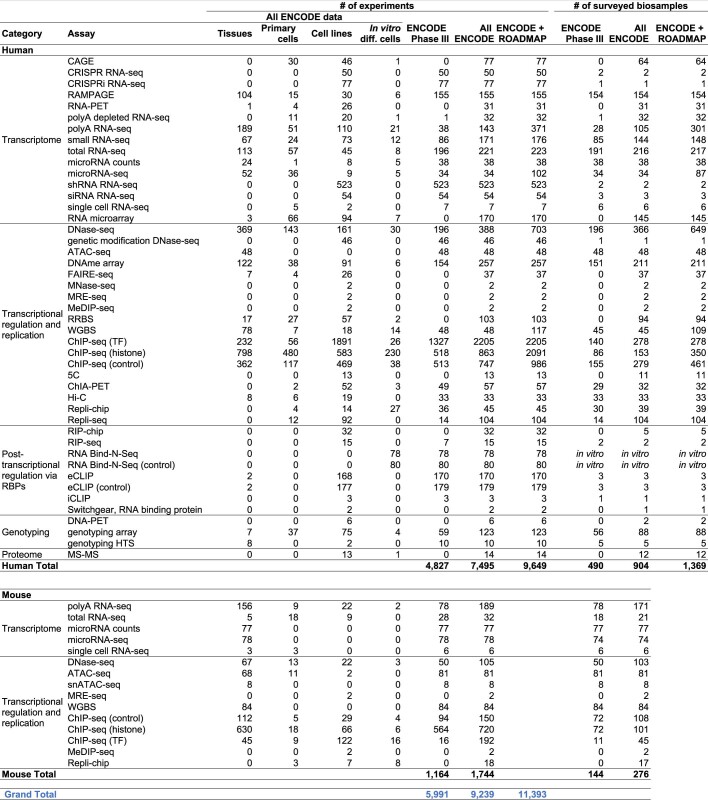
Summary of data produced during ENCODE phase III (as of 1 December 2019)

## Data Availability

All data are available on the ENCODE data portal: www.encodeproject.org.

## Code availability

All code is available on GitHub from the links provided in the methods section. Code related to the Registry of cCREs can be found at https://github.com/weng-lab/ENCODE-cCREs. Code related to SCREEN can be found at https://github.com/weng-lab/SCREEN.
