# Supplementary material for: Expanded encyclopaedias of DNA elements in the human and mouse genomes
Source: Nature. 2020 Jul 29;583(7818):699–710. doi: 10.1038/s41586-020-2493-4 (PMC7410828; doi:10.1038/s41586-020-2493-4)
Supplement: Supplementary file 1 — This file contains Supplementary Notes 1-13, Supplementary Methods, Supplementary References, The ENCODE Project Consortium list, and Supplementary Figures 1-21. [file 41586_2020_2493_MOESM1_ESM.pdf]

nature

https://doi.org/10.1038/s41586-020-2493-4

Supplementary information

Expanded encyclopaedias of DNA elements in the human and mouse genomes

In the format provided by the authors and unedited

Expanded Encyclopedias of DNA Elements in the Human and Mouse Genomes

Supplementary Information Guide

Supplementary Notes 6

Supplementary Note 1. Defining and classifying candidate cis-regulatory elements (cCREs) 6

Supplementary Note 2. Testing various epigenetic signals for predicting enhancers and promoters. 11

Supplementary Note 3. Contribution of ENCODE Phase III data to the Registry of cCREs. 12

Supplementary Note 4. Coverage of the Registry. 13

Supplementary Note 5. Comparison of the Registry of cCREs with other approaches of defining CREs. 14

Supplementary Note 6. Evolutionary conservation of cCREs. 16

Supplementary Note 7. cCREs encompass the binding sites of most transcription factors. 17

Supplementary Note 8. The local transcription landscape at cCREs. 18

Supplementary Note 9. The expression patterns of genes around cCREs. 18

Supplementary Note 10. Differential gene expression and cCRE activity during mouse fetal development. 19

Supplementary Note 11. Testing cCREs with transgenic mouse assays. 21

Supplementary Note 12. Comparing cCREs with active regions identified by high-throughput reporter assays 23

Supplementary Note 13. Using the Registry of cCREs and SCREEN for interpreting GWAS variants. 25

Supplementary Methods 30

Analysis to support the choice of DNase, H3K4me3, and H3K27ac signals for defining cCREs 30

Testing single features for predicting VISTA enhancers 30

Prediction of expression levels using TSS-proximal DNase and histone mark signals 31

Identification and classification of cCREs 32
DNase-seq data curation 32
Calling DNase peaks 32
Filtering DNase peaks 32
Merging DNase peaks to define rDHSs 33
Further filtering of rDHSs by comparing with cDHSs 33
Assigning cCREs to Tiers 33
Total genomic coverage of cCREs 35
Contributions from ENCODE Phases II and III and Roadmap to rDHSs and cCREs 35
cCRE coverage of H3K4me3, H3K27ac, and CTCF ChIP-seq peaks in biosamples without DNase-seq data 36
Overlap of cCREs with ChromHMM states 36
Comparison of cCREs with FANTOM enhancers 37
Evolutionary conservation of cCREs 38
Homologous human and mouse cCREs 38
Repeat and transposon contents of cCREs 39
Transcription factor support for the group classification of cCREs 39
Overlapping of cCREs with transcription factor ChIP-seq peaks 39
Bidirectional transcription at cCREs 40
Gene expression 40
Enrichment of TSS-distal cCREs-ELS near tissue-specific genes 41
Clustering of biosamples by cCREs-dELS 41
Differential gene expression analysis 42
Enrichment of GWAS variants in cCREs 42
Testing cCREs-dELS using transgenic mouse assays 43
Codebase at Github 43
SCREEN 43

Visualizing the ENCODE Encyclopedia via the UCSC genome browser 44

Companion website 45

Supplementary References 46

The ENCODE Project Consortium 50

Supplementary Figures 63

Supplementary Fig. 1. Testing methods for predicting VISTA enhancers and gene expression. 63

Supplementary Fig. 2. Details of building the Registry of cCREs. 64

Supplementary Fig. 3. Classification of cCREs in a particular biosample. 65

Supplementary Fig. 4. UCSC Genome Browser views of cCREs and the underlying DNase and ChIP data. 66

Supplementary Fig. 5. Classification of cCREs into Tiers based on biosample support. 67

Supplementary Fig. 6. Impact of ENCODE Phase III data on the Registry. 68

Supplementary Fig. 7. Coverage of the current Registry of cCREs. 69

Supplementary Fig. 8. Overlap of cCREs with ChromHMM states. 70

Supplementary Fig. 9. Overlap of cCREs with FANTOM Enhancers and the transcription start sites of FANTOM CAGE-associated transcripts. 71

Supplementary Fig. 10. Conservation of human cCREs. 72

Supplementary Fig. 11. Comparison of cCREs with the ChIP-peaks of chromatin-associated proteins and RNA-seq data. 73

Supplementary Fig. 12. Transcription patterns at cCREs. 74

Supplementary Fig. 13. Analyzing differential gene expression and differential cCRE activity across mouse developmental timepoints. 75

Supplementary Fig. 14. Results of testing cCREs-ELS using the *in vivo* transgenic mouse assays. 76

Supplementary Fig. 15. Additional analysis of cCREs tested by transgenic mouse assays and cCREs overlapping regions tested by functional assays MPRA and SuRE. 77

Supplementary Fig. 16. Annotating GWAS variants using cCREs. 78

Supplementary Fig. 17. Using cCREs to annotate functional SNPs related to red blood cell traits. 79

Supplementary Fig. 18. Using cCREs to annotate functional SNPs related to prostate cancer. 80

Supplementary Fig. 19. Using cCREs to annotate functional SNPs related to liver traits. 81

Supplementary Fig. 20. Interpreting GWAS variants associated with neuropsychiatric disorders using cCREs. 82

Supplementary Fig. 21. Method for normalizing epigenomics signals. 83

Supplementary Tables ***see individual Excel files***

Supplementary Table 1. ENCODE phase III transcriptome data

Supplementary Table 2. ENCODE phase III RBP experiments

Supplementary Table 3. ENCODE phase III ChIP-seq of DNA associated proteins

Supplementary Table 4. ENCODE phase III chromatin accessibility experiments

Supplementary Table 5. ENCODE phase III histone ChIP-seq experiments

Supplementary Table 6. ENCODE phase III DNA methylation experiments

Supplementary Table 7. ENCODE phase III 3D chromatin experiments

Supplementary Table 8. ENCODE phase III DNA replication experiments

Supplementary Table 9. Input datasets for building the Registry of cCREs

Supplementary Table 10. Human GRCh38 cCREs

Supplementary Table 11. Mouse mm10 cCREs

Supplementary Table 12. VISTA regions used for evaluating epigenetic signals

Supplementary Table 13. The performance (AUPR) of using epigenetic signals to predict VISTA enhancers

Supplementary Table 14. Using epigenetic signals to predict transcript expression

Supplementary Table 15. Relative abundance of cCREs-PLS vs. cCREs-ELS in 25 human biosamples with full assay coverage

Supplementary Table 16. Regions and genomic positions of ChromHMM promoter and enhancer states that overlap cCREs

Supplementary Table 17. Transposon and repeat content of cCREs

Supplementary Table 18. Overlap of TF ChIP-seq peaks with cCREs

Supplementary Table 19. t-SNE clusters from Extended Data Fig. 4

Supplementary Table 20. cCREs near tissue-specific genes

Supplementary Table 21. RNA-seq experiments included in SCREEN's differential expression tool

Supplementary Table 22. Testing candidate enhancers with mouse transgenic assays

Supplementary Table 23. Genome-wide association studies

Supplementary Notes

Supplementary Note 1. Defining and classifying candidate cis-regulatory elements (cCREs)

Our approach to defining candidate cis-regulatory elements (cCREs) is guided by the understanding that robust biochemical signatures, including chromatin accessibility, particular histone modifications, and the binding of certain transcription factors, are preferentially associated with cis-regulatory elements. Chromatin regions highly accessible to the DNase I endonuclease—DNase hypersensitive sites, or DHSs—are associated with all major classes of cis-regulatory elements[48,71]; therefore, we define cCREs as a subset of representative DHSs (rDHSs), a non-redundant set of DHSs identified in the DNase-seq datasets of 706 human and 173 mouse biosamples surveying diverse cell and tissue types (**Supplementary Table 9**; Supplementary Methods). We further require all cCREs to be flanked by nucleosomes with at least one of two histone marks—histone H3 lysine 4 trimethylation (H3K4me3) or histone H3 lysine 27 acetylation (H3K27ac)—or bound by the transcription factor CTCF (**Fig. 3**); these three types of signals define a cCRE's candidate function. We use H3K4me3 to annotate candidate promoters, as this histone mark is highly enriched around active transcription start sites (TSSs)[41,72,73]. H3K27ac was used to annotate candidate enhancers, as it is known to mark active enhancers as well as active promoters[44,74,75]. CTCF binding sites form their own class; they can serve as insulators that interrupt promoter-enhancer interactions[76,77], or function as an architectural protein facilitating physical interactions between distant chromatin loci[46,78]. Using this classification system (**Fig. 3**), described in detail in the rest of Supplementary Note 1, we identified a total of 0.9 million cCREs in the human genome (**Supplementary Table 10**) and 0.3 million cCREs in the mouse genome (**Supplementary Table 11**), occupying 7.9% and 3.4% of these genomes, respectively, with the smaller number of mouse cCREs owing to a sparser biosample coverage of our mouse epigenetic datasets.

To further test the accuracy of combining DNase, H3K27ac, and H3K4me3 signals for annotating enhancers and promoters, we compared these three features with six other histone marks (H3K4me1, H3K4me2, H3K9ac, H3K36me3, H3K9me3, and H3K27me3) and DNA methylation for predicting enhancer activity and gene expression (Supplementary Methods and detailed in Supplementary Note 2). We assayed all 10 of these epigenetic signals and gene expression in mouse embryonic day 11.5 (e11.5) tissues during this phase of ENCODE; also available were hundreds of enhancers active in each of four mouse e11.5 tissues—midbrain, hindbrain, neural

tube, or limb—identified by transgenic mouse assays from the VISTA database (https://enhancer.lbl.gov/)[79]. The *in vivo* transgenic mouse assays are widely used for evaluating enhancer function, and the assays are high throughput in the tissue axis because they report the enhancer activity for each region in all mouse tissues. At the time of our evaluation (2015), around 2,000 TSS-distal regions in the human and mouse genomes had been tested by e11.5 transgenic mouse assays. Evaluated against these VISTA enhancers (**Supplementary Table 12**), DNase (evaluated on DHS) and H3K27ac signals (averaged over the ± 1 kb window centered on a DHS mid-point) were the best single features for predicting tissue-specific enhancers (**Supplementary Fig. 1a, b** for four features and **Supplementary Table 13** for all 10 features). We then used RNA-seq to evaluate the performance of these 10 epigenetic signals in predicting gene expression levels, and found H3K4me3 (averaged over the ± 1 kb window centered on a DHS mid-point) to be the best single feature (**Supplementary Fig. 1c** for DNase and H3K4me3; **Supplementary Table 14** for all ten features).

Many uses of cCREs are based on the regulatory role associated with their biochemical signatures. Thus, we putatively defined cCREs in one of the following annotation groups based on each element's dominant biochemical signals across all available biosamples (subsequently filtered by per-biosample analysis to yield the final set of cCREs, described below) as well as its proximity to the nearest annotated TSS (**Supplementary Fig. 2a, 2b** for human and **2f, 2g** for mouse):

1) putative *cCREs with promoter-like signatures* (*cCRE-PLS,* GRCh38: 34,803, mm10: 23,762) fall within 200 bp (center to center) of an annotated GENCODE TSS and have high DNase and H3K4me3 signals (evaluated as DNase and H3K4me3 max-Z scores, defined as the maximal DNase or H3K4me3 Z scores across all biosamples with data; see Methods).

2) putative *cCREs with enhancer-like signatures* (*cCRE-ELS*) have high DNase and H3K27ac max-Z scores and must additionally have a low H3K4me3 max-Z score if they are within 200 bp of an annotated TSS. The subset of cCREs-ELS within 2 kb of a TSS is denoted proximal (*cCRE-pELS,* GRCh38: 141,830, mm10: 72,794), while the remaining subset is denoted distal (*cCRE-dELS,* GRCh38: 667,599, mm10: 209,040).

3) putative *DNase-H3K4me3 cCREs* have high H3K4me3 max-Z scores but low H3K27ac max-Z scores and do not fall within 200 bp of a TSS. (GRCh38: 25,537, mm10: 10,383)

4) Finally, putative *CTCF-only cCREs* have high DNase and CTCF max-Z scores and low H3K4me3, H3K27ac, and CTCF max-Z scores. (GRCh38: 56,766, mm10: 23,836)

The four core marks annotating cCREs have different spatial resolutions, which dictate a different approach for computing the signal level for each mark. DHSs are relatively sharp (150-350 bp wide; rDHSs are a subset of DHSs, hence, have the same resolution), corresponding to the core TF binding regions of a regulatory element. CTCF also has a high spatial resolution, and CTCF ChIP-seq peaks often coincide with DHSs. Therefore, we computed DNase and CTCF signals by directly averaging over the rDHS region. H3K4me3 and H3K27ac signals, in contrast, are more diffuse, low at the center of a cCRE, which has open chromatin, and elevated in flanking nucleosomal regions several hundred bps upstream and downstream of the core cCRE. To account for the diffuse signals of H3K4me3 and H3K27ac, we appended 500 bp (corresponding to roughly two nucleosomes) to both sides of each rDHS and computed the levels of H3K4me3 and H3K27ac signals by averaging over the padded region, which were then used to compute their Z-scores in a specific biosample and max-Z scores across all biosamples (Supplementary Methods). In essence, rDHSs specify the locations of cCREs, while H3K4me3, H3K27ac, and CTCF signals suggest the activity types of cCREs.

Analogous to annotating a protein-coding gene irrespective of whether its mRNA is expressed broadly or specifically, this cCRE classification using max-Z scores across all biosamples is agnostic about the identity and number of cell types that provided the chromatin evidence. Building upon this cell type-agnostic cCRE classification, we then classified cCREs on a per-biosample basis using their DNase, H3K4me3, H3K27ac, and CTCF signals in the 25 human and 15 mouse biosamples where all four types of assays were performed. **Supplementary Fig. 3a** and **3b** illustrate the biosample-specific classification in GM12878 cells and the count of each group of cCRE in this biosample. **Supplementary Fig. 3c** delineates the classification for these 25 human and 15 mouse biosamples, and **Supplementary Table 15** lists the counts of each group of cCREs for the 25 human biosamples. Two additional groups are defined for the per-biosample classification: a *low-DNase* group, containing all cCREs with low DNase signals (Z-score < 1.64) in the biosample (regardless the signals of the other three marks), and a *DNase-only* group, containing cCREs with high DNase Z-scores (Z-score ≥ 1.64) but low H3K4me3, H3K27ac, and CTCF Z-scores within that biosample. Therefore, there are seven possible groups (PLS, pELS, dELS, CTCF-only, DNase-H3K4me3, DNase-only, and low-DNase) in a particular biosample, as shown for human cCREs in GM12878 cells (**Supplementary Fig. 3a**). To facilitate subsequent discussions, we collectively call the first six groups of cCREs as high-DNase cCREs in a biosample, sometimes also as cCREs defined in a sample.

Assessed using the 25 fully assayed human biosamples, 13 ± 2% cCREs are in one of the six high-DNase groups and the enhancer groups of cCREs (pELS and dELS together) outnumber the promoter group (PLS) by three-fold, and a higher fraction of PLS cCREs are shared among biosamples than ELS cCREs (**Supplementary Table 15**). We further compared the cell type specificity among the six groups of high-DNase cCREs across the 25 biosamples, and as expected, cCREs-PLS are the least cell type-specific and cCREs-dELS and DNase-H3K4me3 cCREs are the most cell type-specific (**Supplementary Fig. 3d**). These group classifications are supported by the binding of many chromatin-associated proteins, especially RNA polymerase (Pol) II, EP300 (a histone acetyltransferase that prefers to bind enhancers), and RAD21 (another chromatin architecture protein like CTCF). cCREs-PLS are most enriched in Pol II binding, while cCREs-ELS are most enriched in EP300 binding but have a weak Pol II signal, and CTCF-only cCREs are most enriched in the RAD21 signal (**Extended Data Fig. 2d**).

The per-biosample classification is illustrated for three cell type-specific loci in humans: *SPI1* in B cells, *NPAS4* in bipolar neurons, and *HNF4* in hepatocytes (**Supplementary Fig. 4**). *SPI1* encodes the transcription factor PU.1, which activates genes during B cell development. The *SPI1* locus shows three cCREs, one PLS at its promoter, one upstream dELS, and one downstream CTCF-only. The PLS and dELS show high histone mark signals only in B cells and are classified as low-DNase in bipolar neurons and hepatocytes (**Supplementary Fig. 4a**), consistent with the function of *SPI1* in these cell types*.* NPAS (Neuronal PAS Domain Protein 4) is a transcription factor that functions in both excitatory and inhibitory brain neurons. Accordingly, in bipolar neurons, the *NPAS* locus has one PLS and three DNase-H3K4me3 cCREs at the promoter and two CTCF-only cCREs up and down-stream. The PLS and DNase-H3K4me3 cCREs show no signal in B cells and hepatocytes. However, there is a DNase-only cCRE in the first intron of *NPAS* in B cells and hepatocytes and another DNase-only cCRE upstream in hepatocytes. We asked what transcription factors might bind to these DNase-only cCREs and found that they corresponded to two high-signal ChIP-seq peaks of NRSF (Neural-Restrictive Silencer Factor, also named RE1 Silencing Transcription Factor or REST) in GM12878 and HepG2, two cell lines in the same lineages as B cells and hepatocytes, respectively (**Supplementary Fig. 4b**). Thus, we hypothesize that the *NPAS* locus is repressed by NRSF in non-neuronal cell types. HNF4A (Hepatocyte Nuclear Factor 4 Alpha) is a transcription factor that regulates hepatic genes. Its promoter is surrounded by one PLS and three ELS in hepatocytes, and all of these cCREs have low signals in B cells and bipolar neurons. In summary, the per-biosample classification of cCREs

distills the information in the four core assays to suggest the regulatory functions of key genomic elements.

In addition to the 25 human biosamples and 15 mouse biosamples that are fully covered by the four assays (which we call Type A samples), hundreds of additional samples had partial coverage by ENCODE data. We assigned these samples as Type B (with DNase and one or two other marks), Type C (no DNase but one to three other marks) and Type D (DNase but no other marks), and devised a tier system (**Box 1** and **Supplementary Fig. 5**) for the final selection of cCREs from putative cCREs, with the latter based on maximal signal across all biosamples as defined above. The cCREs defined in fully covered (Type A) biosamples are designated as Tier 1a (N = 534,913 in human and 244,595 in mouse), reflecting our highest confidence in them. Type B biosamples can also define cCREs that have high signals of DNase and one other mark in the *same* biosample, but these cCREs are assigned Tier 1b (N = 179,048 in human and 22,245 in mouse), reflecting the incomplete assay coverage of the biosamples from which they came. Finally, we pair up a Type C biosample (using its histone mark or CTCF data) with a Type B or D biosample (using its DNase data) to define Tier 2 cCREs (N = 212,574 in human and 50,975 in mouse) that do *not* have high signals of DNase and one other mark in the *same* biosample because of missing data. All combinations of sample coverage for Tier 2 cCREs are enumerated in **Supplementary Fig. 5**. With Tiers 1a, 1b, and 2 combined, there are 926,535 human and 339,815 mouse cCREs, occupying 7.9% and 3.4% of the human and mouse genomes, respectively (**Supplementary Fig. 2c, d** for human and **2h, i** for mouse).

For Type B and D biosamples, which had DNase-seq data but were missing one or more ChIP-seq data types, we provide partial assignment of cCREs (**Supplementary Fig. 3e**). Because our cCRE classification requires DNase signal, we do not provide per-biosample cCRE classification for Type C biosamples and just designate the presence of high or low signals (**Supplementary Fig. 3f**).

The tier assignments largely reflect the assay availability but not biological differences between the two organisms (**Supplementary Fig. 2e, j**). Users of the Registry of cCREs can use the tier system to filter cCRE sets according to the completeness of the supporting data. As more DNase-seq and ChIP-seq data become available, we will update the tier assignments of all cCREs; meanwhile, we distinguish low signal from missing data while annotating cCREs in individual biosamples and in the analyses throughout this paper, and in the SCREEN web resource.

Supplementary Note 2. Testing various epigenetic signals for predicting enhancers and promoters.

We asked which of the ten epigenetic signals were predictive of VISTA enhancers in each of four tissues: midbrain, hindbrain, neural tube, and limb. For each of these four tissues, our positive test set comprised all VISTA regions that tested positive in that tissue, while our negative set contained the remaining VISTA regions (most of the negative regions showed no activity in any tissue, while a small number of negative regions showed activity in another tissue). The VISTA regions used in our analysis are listed in **Supplementary Table 12**, and the method of comparison is detailed in Supplementary Methods. We first tested two ways of anchoring the window for computing the average signal of DNase or the histone marks: DHSs or the ChIP-seq peak summit of five histone marks known to have relatively punctate peaks (H3K27ac, H3K9ac, H3K4me3, H3K4me2, and H3K4me1). We found that, on average, anchoring on DHSs led to slightly but consistently better performance (evaluated as the area under the precision-recall curve or AUPR; **Supplementary Table 13a** and **Supplementary Fig. 1a, b**). These results verify our rationale that DHSs have higher resolution than histone marks. Next, anchoring the window on DHSs, we evaluated all ten epigenetic signals for predicting VISTA enhancers (**Supplementary Table 13b**). DNase signal was the most predictive feature for enhancer activity in three of four tissues (hindbrain, neural tube, and limb), with the AUPR = 0.38, 0.31, and 0.45, respectively, while for midbrain enhancers, H3K27ac (AUPR = 0.42) was the most predictive feature followed by DNase (AUPR = 0.39; **Supplementary Table 13b** and **Supplementary Fig. 1b**). Signals of other histone marks and DNA methylation achieved average AUPR ranging from 0.26 by H3K4me1 to 0.15 by DNA methylation (**Supplementary Table 13b**).

We further tested averaging the rank by DNase and the rank by each of the other nine signals, still anchoring enhancer predictions on DHSs (**Supplementary Table 13b**). The average rank of the DNase and H3K27ac signals resulted in the same AUPR as ranking by DNase signal alone (average AUPR = 0.38). The average rank of DNase and another signal improved AUPR over the rank of the other signal alone, e.g., the average AUPR of the average rank of DNase and H3K4me1 was 0.34, compared with 0.26 for ranking by H3K4me1 alone. Incorporating additional histone marks or DNA methylation using a linear model did not further improve performance (data not shown). We did not test more complex models because of the small number of VISTA enhancers—only 200-300 genomic regions tested positive in each tissue.

We further evaluated whether an adaptation of the above-described enhancer prediction model could be used to map cell type-specific promoter regions, judged by the correlation between the level of each epigenetic signal and the nearest transcript's expression level measured by RNA-seq in the e11.5 midbrain, hindbrain, neural tube and limb. Again, we tested two ways of anchoring the epigenetic signals, on DHSs or H3K4me3 peaks, and evaluated the aforementioned ten features—DNase, eight histone marks, and DNA methylation (Supplementary Methods). When anchored on TSS-proximal DHSs, H3K4me3 correlates the strongest with expression level (Spearman's correlation coefficient ρ = 0.75 averaged over the four tissues), slightly better than H3K9ac (ρ = 0.74), but substantially better than the remaining marks (**Supplementary Table 14a**; **Supplementary Fig. 1c** for the midbrain). This correlation is substantially higher than that of the H3K4me3 signal centered on H3K4me3 peaks (ρ = 0.60) or the DNase signal centered on TSS-proximal DHSs (ρ = 0.45). Repeating this analysis with human RNA-seq data in GM12878, K562, and HepG2 cells yielded consistent results (ρ = 0.72, 0.73, and 0.71, respectively; **Supplementary Table 14b**). In conclusion, the high spatial precision offered by DHSs improves the accuracy of H3K4me3 for predicting gene expression levels.

Supplementary Note 3. Contribution of ENCODE Phase III data to the Registry of cCREs.

The additional data from ENCODE Phase III greatly increased the comprehensiveness of the Registry of human cCREs. The numbers of high-signal rDHSs that would have resulted from using only ENCODE Phase II data (1.3 M), only Roadmap Epigenomics data (1.3 M), or a combination of these two sets of data (1.7 M) are substantially lower than the number (2.1 M) when ENCODE Phase III data are included (**Supplementary Fig. 6a**). Saturation analysis of cCREs stratified by class suggests that with the current collection of datasets we are nearly saturated for cCREs-PLS, DNase-H3K4me3, and CTCF-only but not for cCRE-ELS, particularly cCREs-dELS (**Supplementary Fig. 6b**). Accordingly, ENCODE III data increased the number of human cCREs by 22% compared with ENCODE II and Roadmap combined, with the increase most evident for dELS, DNase-H3K4me3, and CTCF-only cCREs (**Supplementary Fig. 6c**). The shift of data production in ENCODE Phase III to focus on primary cells and tissues was one reason behind the increased coverage of cCREs. We defined human cCREs using just cell line data, just primary cell data, or just tissue data. Data from tissues substantially augmented the counts of all categories of cCREs, and primary cell data made further contributions to the cCRE count (**Supplementary Fig. 6d**). In addition to the quantitative increase in cCREs, the tissue and

primary cell data greatly expanded the diversity of biosamples covered by ENCODE cCREs, which should aid investigations of transcriptional regulation in more cell and tissue types.

Supplementary Note 4. Coverage of the Registry.

Our working hypothesis in defining the Registry of cCREs based on rDHSs is that a collection of rDHSs derived from hundreds of DNase-seq experiments would represent a large fraction of all cis-regulatory elements in the genome and that a new biosample is likely to use as its regulatory repertoire a subset of the cCREs already in the Registry. To test this hypothesis, we examined the chromatin and CTCF ChIP-seq data from human biosamples that lacked DNase-seq data and found that, on average, 93% of H3K4me3 peaks, 83% of H3K27ac peaks, and 97% of CTCF peaks called in these cell types (false discovery rate or FDR < 0.01) corresponded to a cCRE, i.e., they were detected in another cell type with DNase-seq data (**Supplementary Fig. 7a-c**). Similarly, among the mouse biosamples that had chromatin and CTCF data but lacked DNase data, 90% of H3K4me3 peaks, 70% of H3K27ac peaks, and 83% of CTCF peaks overlapped cCREs (**Supplementary Fig. 7g-i**). The slightly lower percentages in mouse than in human were due to the fewer number of DNase-seq datasets from mouse and correspondingly the lower coverage of the mouse Registry. To investigate why some ChIP-seq datasets had poor coverage by the Registry, we plotted the percent overlap of cCRE-overlapping ChIP-seq peaks in a dataset against the average -log(FDR) of all peaks in the dataset, which is an indicator of the average quality of ChIP-seq peaks identified by the peak calling algorithm. The biosamples with poor Registry coverage tended to have low average -log(FDR) in both human and mouse (**Supplementary Fig. 7d-f, 7j-l**). We visually inspected two mouse H3K4me3 datasets with the lowest coverage (CD-1 megakaryocyte and GR1-ER4 in mouse, **Supplementary Fig. 7j**) and confirmed that the peaks that were not covered by cCREs had low signals and were likely false positives by the peak calling algorithm. The low-coverage CTCF dataset in human subcutaneous adipose tissue (**Supplementary Fig. 7f**) also had low quality, with only 736 peaks.

To further evaluate the human Registry, we compared it with the ~101,000 TSSs annotated by GENCODE[59] and the ~84,000 regulatory elements identified by the FANTOM Consortium[80] using the Cap Analysis of Gene Expression (CAGE) assay[58]. We found that 50% of human GENCODE TSSs and 70% of stringent FANTOM CAGE clusters were contained within a human cCRE. The FANTOM TSSs that did not overlap a cCRE were usually detected in a cell or tissue type that was not represented or fully covered by four assays in the ENCODE dataset, e.g., subtypes of neuronal cells, reproductive tissues, and pituitary gland. These data support the

comprehensiveness of the ~900,000 annotated cCREs in our human Registry, but also suggest that additional cCREs will be found in cell types yet to be assayed by ENCODE. In particular, the current human Registry is likely to underrepresent cCREs specific to embryonic tissues, stem and progenitor cells, specialized brain cells, dynamic responses, and disease states. Closing this gap will require isolation and characterization of such cell states, a significant undertaking that should be aided by new biological understandings of the nature and complexity of human tissues, new experimental models that include the ability to differentiate pluripotent cells into many other cell types, and new technologies for profiling low-cell-count samples and single cells.

In conclusion, the human Registry of cCREs appears to be comprehensive: It covers more than 80% of elements marked by H3K4me3 or H3K27ac or bound by CTCF (FDR < 0.01) in any biosample and 50-70% TSSs in GENCODE and FANTOM collections. A cautionary note is that we do not yet know the extent of our coverage of highly cell-type-specific cCREs that are active in rare cell types (numerically minor in their tissues of origin) that have not yet been sensitively assayed. The mouse Registry is less comprehensive than the human Registry, but we expect that it will continue to grow with experiments performed on additional biosamples.

Supplementary Note 5. Comparison of the Registry of cCREs with other approaches of defining CREs.

There are many approaches that can be used to identify regulatory elements by using epigenetic signals. ENCODE Phase II developed machine-learning techniques such as ChromHMM[81] and Segway[82], while the FANTOM Consortium leveraged the distinct transcriptional signatures at regulatory elements[83]. Our approach for building the cCRE Registry is simpler but allows broader coverage of cell types when assay coverage is sparse. Overall, our cCRE predictions compare favorably with the ChromHMM-based and FANTOM-produced collections.

We first compared cCRE calls with ChromHMM states in the same cell or tissue type. For human cCREs, we performed the comparison in GM12878, which was an ENCODE II tier I cell type[1] and continued to be used for many assays in ENCODE III. Most cCREs-PLS (86%) defined in GM12878 overlapped a ChromHMM promoter also defined in GM12878, and most cCREs-dELS (83%) overlapped a ChromHMM enhancer (**Supplementary Fig. 8a**). By comparison, most cCREs-pELS were classified as promoters by ChromHMM (80% pELS overlapped ChromHMM promoters, and 19% pELS overlapped ChromHMM enhancers), because the low spatial resolution of ChromHMM states caused their promoters to “spill over” to the neighboring cCREs-

pELS (**Supplementary Fig. 8b**). Furthermore, DNase-H3K4me3 cCREs mostly overlap ChromHMM TSS, CTCF-only cCREs mostly overlap ChromHMM insulators, and DNase-only cCREs mostly overlap low-signal ChromHMM enhancers, providing additional information on these groups of cCREs. Reciprocally, ChromHMM states were enriched in their corresponding cCRE types, although they had larger overall genome footprints than did cCREs (**Supplementary Table 16a, c**).

We observed similarly strong agreement between the cCREs defined in five e11.5 and six e14.5 mouse tissues with their respective ChromHMM states called using eight histone marks (H3K4me1, H3K4me2, H3K4me3, H3K9ac, H3K27ac, H3K36me3, H3K9me3, and H3K27me3) in the corresponding tissues and time points [84]. On average, 98% of cCREs-PLS overlapped ChromHMM promoters, and 67% of cCREs-ELS overlapped ChromHMM enhancers (**Supplementary Fig. 8c**). DNase-H3K4me3 cCREs mostly overlap the ChromHMM TSS and bivalent-TSS states (61% and 34%, respectively). Reciprocally, the ChromHMM TSS and bivalent-TSS states overlapped most extensively with PLS as opposed to other groups of cCREs, while the high-signal enhancer ChromHMM state overlapped most extensively with ELS (**Supplementary Table 16b, d**). The positions in TSS, enhancer, and insulator ChromHMM states that overlapped cCREs had significantly higher evolutionary conservation than the positions in the corresponding ChromHMM states that did not overlap cCREs, suggesting the higher resolution of cCRE pinpointed the most relevant functional sequences (**Supplementary Table 16e**).

We also compared cCREs-ELS with the 65,423 candidate human enhancers defined by the FANTOM Consortium. Their predictions are quite different in origin, being based entirely on CAGE data and having been drawn from hundreds of cell and tissue types[80,83], with which ENCODE has only partial overlap. Nevertheless, 66% of the FANTOM enhancer set overlapped with a cCRE. The intersect set of cCREs with FANTOM enhancers (~5% of cCREs) also display significantly higher chromatin and Pol II signals (**Supplementary Fig. 9a-e**) and higher evolutionary conservation (**Supplementary Fig. 10c**) than the remaining 95% of cCREs. Thus, the FANTOM set seems to correspond in several aspects to our top cCREs.

Given the importance of transcription at enhancer elements, we further sought to quantify transcribed cCREs-ELS and to elucidate the differences between the transcriptional patterns at cCREs-ELS and cCREs-PLS, using the 59,011 coding and non-coding RNAs in the FANTOM

CAGE Associated Transcriptome (CAT) collection[80]. These FANTOM CAT transcripts fall into eleven categories, and we observed large differences between the categories in terms of the percentage of CAT transcripts in the category with their 5´-ends within 2 kb of a cCRE. The CAT transcripts in the protein-coding and small-RNA categories had their 5´-ends predominantly near cCREs-PLS, while the CAT transcripts in the eRNA-like categories[80] had their 5´-ends predominantly near cCREs-dELS (**Extended Data Fig. 3e**, redrawn in **Supplementary Fig. 9f**). Overall, a majority of FANTOM CAT transcripts were annotated by cCREs. Because cCREs-ELS vastly outnumber FANTOM CAT RNAs, ~19% of cCREs-ELS fall within 2 kb of the TSS of a FANTOM CAT lncRNA.

In summary, our Registry of cCREs overlaps significantly with existing collections of regulatory elements, yet has its distinct advantages—it has higher resolution than ChromHMM states and is more comprehensive than FANTOM enhancers and CAT RNAs.

Supplementary Note 6. Evolutionary conservation of cCREs.

Our prediction of cCREs from biochemical features, not from interspecies comparisons, allowed an unbiased examination of their evolutionary conservation. Focusing on the 8% of the human genome that is evolutionarily highly constrained (GERP++ sites[85]), the percentage of constrained nucleotides in each group of cCREs correlated positively with the major biochemical signal (DNase-seq; **Supplementary Fig. 10a**). Judged by average phyloP score[69], a quantitative measure of evolutionary conservation, all groups of cCREs are more conserved than randomly chosen genomic regions (3-8 times by group; **Extended Data Fig. 2b**). We also mapped out the correspondence between human and mouse cCREs using LiftOver (**Extended Data Fig. 2c**), and the cCREs that have homologs in the other species have higher phyloP scores than the cCREs that do not have homologs in the other species (**Supplementary Fig. 10b**). The entire sets of human and mouse cCREs were depleted in repetitive elements (**Supplementary Table 17**), but, consistent with previous reports[86,87], the human CTCF-only cCREs were enriched in long terminal repeats (1.6 fold; Chi-square *p*-value = 4.0E-77) and the mouse CTCF-only cCREs in SINE elements (1.7 fold; *p*-value = 4.5E-40). Thus, cCREs show a strong trend of evolutionary conservation that is associated with the strength of the predictive signal, but many individual cCREs are species specific.

Supplementary Note 7. cCREs encompass the binding sites of most transcription factors.

Our definition of cCREs does not incorporate transcription factor binding information other than that of CTCF, allowing for unbiased integrative analyses of cCREs with the wealth of ENCODE ChIP-seq data on chromatin-associated proteins, most of which are transcription factors (TFs). ENCODE aims to generate ChIP-seq data for as many TFs as possible; these data have been processed by an ENCODE uniform pipeline to define ChIP-seq peaks, genomic regions significantly bound by a TF. Overall, there is excellent overlap between TF ChIP-seq peaks and cCREs—a median ENCODE TF ChIP-seq dataset has 90% of its peaks overlap a cell type-agnostic cCRE (**Extended Data Fig. 3b**, redrawn in **Supplementary Fig. 11a** for reference). There are only 12% of ChIP-seq experiments for which fewer than 70% of peaks overlapped cCREs, and many of these experiments belonged to TFs with known repressive activities, e.g., MAFF, MAFK, ZNF274, and ATF7 (**Supplementary Table 18**). Three cell lines, K562, HepG2, and GM12878, have been extensively assayed by ENCODE for TF ChIP-seq, with hundreds of factors profiled in each. We observed high overlap between TF peaks with cell type-specific cCREs predicted to be active in these cell types, with median TF ChIP-seq datasets in GM12878, HepG2, and K562 having 78%, 84%, 74% of their peaks overlapping cCREs in the respective cell types (**Supplementary Fig. 11b**). Given that, on average, only 13 ± 2% of all cCREs have high-DNase signals in a particular biosample (**Supplementary Table 15**), the high percentages of TF peaks overlapping cCREs in the corresponding cell type support the high accuracy of our approach to classifying cell type-specific cCREs. Many of the experiments with low overlaps with cell type-specific cCREs also belonged to TFs with repressive activities.

We further examined the overlap between cCREs and genomic regions bound by multiple TFs according to the TF ChIP-seq data (**Supplementary Fig. 11c**). In each of the three aforementioned cell types, we observed that genomic regions bound by more TFs were more likely to overlap cCREs (both cell type-agnostic and cell type-specific cCREs). We also analyzed each group of cCREs (**Supplementary Fig. 11d**, in HepG2 cells) and found that cCREs-PLS were bound by the most TFs (median = 39 TFs in HepG2 cells) followed by cCREs-pELS and cCREs-dELS (median = 22 and 29 TFs respectively). All six groups of cCREs overlapped more TF peaks than Low-DNase cCREs (p < 1E-230 in HepG2 cells, Wilcoxon rank-sum tests). As expected, the CTCF-only cCREs were specifically enriched in the binding of CTCF and other cohesin components (Rad21, SMC3). These analyses indicate that the Registry of cCREs

captures the majority of the TF cis-regulatory landscape and also reveals the need for including data from more repressive factors in improving the Registry of elements.

Supplementary Note 8. The local transcription landscape at cCREs.

We explored the transcriptional landscape of nascent RNAs surrounding cCREs using public GRO-seq data in GM12878 and PRO-seq data in primary CD4+ T-cells[88,89]. Both cCREs-PLS and cCREs-dELS exhibit evidence of bidirectional transcription, albeit with distinct patterns and levels (**Extended Data Fig. 3c-d**, and **Supplementary Fig. 12a-d**, with a and c being redrawn for reference). cCREs-PLS show a strong burst of asymmetrical transcription, with the sense-strand signal peaking higher than the antisense-strand signal. The transcription level around cCREs-dELS, on average, is maximal at 10% of the highest level around cCREs-PLS.

We further observed that the bidirectional transcription patterns were consistent with our definition of cCREs in a cell type-specific manner. The cCREs defined in both CD4+ T cells and GM12878 cells tended to show bidirectional transcription in both cell types (**Supplementary Fig. 12e-f**, two left panels), while the cCREs defined in only one of the two cell types exhibited bidirectional transcription more strongly in their defining cell type than in the other cell type (**Supplementary Fig. 12e-f**, comparing the solid bars, the two middle panels have higher percentages in CD4+ T cells than in GM12878 cells, whereas the two right panels have higher percentages in GM12878 cells than in CD4+ T cells). Overall, a large majority of cCREs-ELS show bidirectional transcription (92.2% in GM12878 and 76.6% in CD4+ T cells; **Supplementary Fig. 12**). Our results suggest that nascent transcriptional patterns at cCREs are correlated with their epigenetic profiles in a cell type-specific manner. The incorporation of such information offers a fruitful avenue for future research in defining and classifying candidate regulatory elements, and we are exploring such approaches during ENCODE IV.

Supplementary Note 9. The expression patterns of genes around cCREs.

Genes near cCREs defined in a biosample tend to be expressed in that biosample. To focus our analysis, we examined three human cell types from distinct lineages—cardiac muscle cells, hepatocytes, and neural progenitors—and compared the cCREs-PLS predicted to be active in each cell type (by DNase and H3K4me3 signals) with genes expressed in the corresponding cell type (measured by RNA-seq). We found that genes with TSSs (GENCODE V24 basic TSSs) overlapping cCREs-PLS had the highest expression, followed by genes with TSSs overlapping other high-DNase cCREs, then genes with TSSs overlapping low-DNase cCREs

(**Supplementary Fig. 11e**). Genes without a cCRE at their TSSs had the lowest overall expression in all three cell types. Reciprocally, most genes (~85%) expressed in each cell type (> 1 tag per million; TPM) had a cell type-specific cCRE-PLS at their TSS (**Supplementary Fig. 11f**).

The high resolution of our cCREs allowed us to investigate their nearby transcriptional activities at the TSS level. RAMPAGE is a 5´-complete cDNA sequencing assay developed during ENCODE III that can capture the transcript levels of individual TSSs[18], and eight of the 25 human biosamples covered by DNase, H3K4me3, H3K27ac, and CTCF assays also have RAMPAGE data. We found that cCREs-PLS were enriched for the strongest RAMPAGE peaks (median among the eight biosamples: 51% cCREs-PLS overlapped RAMPAGE peaks and TSS expression at these RAMPAGE peaks = 14.2 TPM), followed by cCREs-pELS (8.6% cCREs-pELS overlapped RAMPAGE peaks; TSS expression = 3.3 TPM), while other groups of cCREs showed even lower overlap with RAMPAGE peaks than cCREs-pELS although at similar median expression levels (~3 TPM; **Extended Data Fig. 3a**). These results indicate that our cCREs-PLS correspond to active TSSs and our classification of cCREs-pELS as a subtype of ELS and not a subtype of PLS is consistent with transcription.

We asked which group of cCREs preferred to be located near tissue-specific genes as opposed to housekeeping genes, defined using the RNA-seq data across tissue-timepoints in the mouse developmental series (1,000 genes with the highest and lowest tissue specificity, respectively; see Methods). We found that a significantly higher percentage of housekeeping genes had a nearby cCRE-pELS than did tissue-specific genes (median = 60% vs. 31%; p = 7.5E-9) and the reverse was true for cCRE-dELS (5.9% vs. 13.4%; p = 1.7E-7; **Supplementary Fig. 11g** and **Supplementary Table 20**). For the remaining groups of cCREs, PLS follows the same trend as pELS, while DNase-H3K4me3 and DNase-only follow the same trend as dELS (**Supplementary Table 20**). These results suggest that tissue-specific genes and housekeeping genes may be regulated by different types of cCREs-ELS, with housekeeping genes predominantly by cCREs-pELS, while tissue-specific genes by both cCREs-pELS and cCREs-dELS.

Supplementary Note 10. Differential gene expression and cCRE activity during mouse fetal development.

To study the epigenetic landscape of mammalian development, we performed ChIP-seq of eight histone marks and RNA-seq at daily intervals between embryonic day 10.5 (e10.5) and postnatal

day 0 (P0), with 6-12 tissues sampled per day, totaling 66 samples. This wealth of data offers a highly detailed picture of the impact of regulatory element activity on gene expression during development. We performed differential gene expression analysis between all available pairs of tissues and embryonic timepoints (Supplementary Methods; **Supplementary Table 21** lists the RNA-seq datasets used) and investigated the differential epigenetic signal profiles of cCREs surrounding the differentially expressed genes. The results of this systematic analysis can be accessed via a web-based resource called SCREEN (Search Candidate cis-Regulatory Elements by ENCODE; http://screen.encodeproject.org, **Box 2**), and the locus centered on each differentially expressed gene can be visualized along with the differential H3K27ac signals of cCREs at the locus, providing a resource for the exploration of gene-cCRE relationships.

We illustrate the utility of this differential gene-cCRE resource with mouse cCRE EM10E0842983, which may function as an enhancer regulating *Apoe*, a protein expressed in the liver and involved in cholesterol metabolism[90]. The *Apoe* locus depicted by SCREEN reveals that the expression of *Apoe* (in $\log_2$TPM) is 5.1-fold higher at P0 than at e11.5 (**Supplementary Fig. 13a**), supporting previous findings of increased *Apoe* expression at birth in rats[91]. Nearby apolipoprotein C genes *Apoc1*, *Apoc2*, and *Apoc4* are likewise overexpressed at P0 (green boxes in **Supplementary Fig. 13a**). Accordingly, cCREs-PLS and cCREs-ELS also show higher H3K4me3 and H3K27ac signals at P0 than at e11.5 (red and yellow dots in **Supplementary Fig. 13a**). Among these cCREs, an ELS, EM10E0842983, overlaps a mouse hepatic control region (HCR). The H3K27ac signal at EM10E0842983 is highly correlated with *Apoe* expression across the seven developmental time points (Pearson's correlation $r = 0.94$; p-value = 1.3E-3; **Supplementary Fig. 13b, c**). EM10E0289438 has high H3K27ac signal primarily in the liver (**Supplementary Fig. 13d** shows the mouse biosamples with the highest H3K27ac signals).

The mouse HCR that contains EM10E0842983 is homologous to two human HCRs (HCR.1 and HCR.2), which are 21 kb downstream of *APOE*'s TSS[92]. HCR.1 AND HCR.2 have high sequence similarity (85%) as a result of a 10 kb duplication of the region around *APOC1*. EM10E0842983 has three homologous human cCREs-ELS: EH38E1957033 in HCR.1 and EH38E1957037 and EH38E1957038 in HCR.2 (**Supplementary Fig. 13e, f**). Previous studies demonstrated that human HCR.1 and HCR.2 differ in tissue specificity. In transgenic mice, HCR.1 led to a broad *Apoe* expression, including hepatic and non-hepatic tissues, while HCR.2 resulted in a liver-specific *Apoe* expression[92]. Consistent with these previous results, SCREEN illustrates that the human cCRE that overlap HCR.1 (EH38E1957033) has high H3K27ac signals in liver and many

other tissue types, such as the gastrointestinal system and brain, whereas the two cCREs in HCR.2 (EH38E1957037 and EH38E1957038) have liver-specific H3K27ac signals (**Supplementary Fig. 13e,f**).

As illustrated in this example, integration of differential gene expression with the differential epigenetic signals of nearby cCREs across a panel of cell and tissue types, in particular in the context of human-mouse comparison, can aid the identification of cCREs that regulate gene expression programs. The mouse developmental series data provide time courses for gene expression and cCRE epigenetic signals, and a high correlation between the two types of time courses provides support for their regulatory relationship. The difference in tissue specificity between the mouse cCRE and the three homologous human cCREs in this example also provides a glimpse of evolutionary innovation—one of the human cCREs (EH38E1957033) is no longer liver-specific, and it may have taken on a different function from the other cCREs.

Supplementary Note 11. Testing cCREs with transgenic mouse assays.

To assess the accuracy of cCRE with enhancer-like signatures (ELS), we experimentally tested 151 genomic regions, each centered on a cCREs-ELS, using transgenic mouse assays[49]. We used the average rank of the DNase and H3K27ac signals to identify previously untested, TSS-distal (> 2 kb from the nearest GENCODE-annotated transcription start site or TSS) cCREs-ELS in the mouse e11.5 midbrain, hindbrain, and limb, and the boundaries of the tested regions were defined using the overlapping H3K27ac ChIP-seq peaks (Supplementary Methods). An initial transgenic reporter survey by ENCODE found that active constructs are concentrated in the top quartile of H3K27ac signal[52]. To explore this relationship further, for each tissue, we tested 20, 15 and 15 regions around the ranks of 1-20, 1,500-1,520, and 3,000-3,020, respectively. The results are in **Supplementary Table 22a-c**, and representative e11.5 transgenic mouse embryos for the enhancers that validated in the expected tissues are shown in **Supplementary Fig. 14**. The rank based on DNase and H3K27ac could predict activity with reasonable precision, as indicated by the area under the precision-recall curve of 0.67, 0.56, and 0.59 for midbrain, hindbrain, and limb, respectively, substantially higher than the values at ~0.3 for random predictions (**Supplementary Fig. 15a**). Consistently, higher ranking regions were more likely than lower ranking regions to show enhancer activity in their predicted tissue (**Fig. 4a**; e.g., 60% test positive for ranks 1-20, 40% for ranks 1,500-1,520, and 27% for ranks 3,000-3,020 in midbrain). This testing was performed when our Registry of cCREs was first established, and we subsequently improved our method for building the Registry (moving to the newer human genome build GRCh38, improving

our method for defining rDHSs, and adding the TSS-proximal ELS or pELS and DNase-H3K4me3 groups of cCREs), and a reanalysis with the current version of cCREs confirmed this correlation between epigenetic signal and validation rate (**Supplementary Fig. 15b**). For completeness, the original ranks and new ranks are provided in **Supplementary Table 22d-f**.

In a parallel study described in a companion paper[14], regions based on H3K27ac peaks in e12.5 forebrain (N = 50), heart (N = 45) and limb (N = 45) tissues were selected for testing using e12.5 transgenic mouse assays (these regions are listed in **Supplementary Table 22g**). We analyzed these data prospectively and observed similar validation rates for cCREs defined in the corresponding tissues (**Supplementary Fig. 15c**) as for e11.5 cCREs described above. The e12.5 cCREs in the corresponding tissues that map to the tested regions are listed in **Supplementary Table 22h-j**. A study described in another companion paper[52] tested a set of human regions and some mouse regions. We also analyzed the mouse regions prospectively (**Supplementary Fig. 15d**) and list all of their tested regions in **Supplementary Table 22k-l** along with the cCREs that overlap them. In total, **Supplementary Table 22** summarizes all transgenic mouse experiments performed during ENCODE Phase III.

When a predicted enhancer region tested as active in multiple tissues, these tissues typically had high H3K27ac signals across the region (**Fig. 4b**). For example, a predicted enhancer in the hindbrain (**Fig. 4b**, mm1489) was also active in the midbrain and neural tube, and accordingly, high H3K27ac signals were observed in all three neuronal tissues (H3K27ac data for midbrain and hindbrain are shown in **Fig. 4b** but not shown for neural tube). More often, even though high H3K27ac signals were observed in multiple tissues, reporter activity was detected in only one or a subset of these tissues. For example, mm1502 was active only in midbrain, but high H3K27ac signals were observed in other brain tissues as well, and mm1444 was active in hindbrain and midbrain, but high H3K27ac signals were observed even in non-brain tissues as well. In contrast, an enhancer that was exclusively active in the limb (mm1492 in **Fig. 4b**) showed high H3K27ac signals only in the limb. These results suggest that cCREs-ELS defined using DNase and H3K27ac signals correspond to active enhancers whose tissue selectivity patterns often match or reflect a tissue-subset of their H3K27ac signal patterns.

The overall validation rates for these predictions (43-46% across the three tissues) are lower than those of an earlier study[93] (78-82% in forebrain, limb, and midbrain) but higher than those of two other earlier studies—32% in forebrain[94] and 38% in heart[95] using transgenic mouse assays. The

higher validation rate by Visel et al. may be partly due to the requirement of evolutionary conservation in picking the regions for testing[93], which was not imposed on our enhancer predictions. Our results support this hypothesis. If we stratify our tested regions by conservation (**Supplementary Fig. 15e**), we observe that all highly conserved elements (average phyloP score across the element ≥ 1) tested positive (*p* = 0.03, Fisher's Exact Test). The enhancer predictions that yield negative results in transgenic mouse assays could be due to a number of reasons: false-positive predictions, enhancers that are longer than the fragments that were able to be tested, enhancers active at other time points, enhancers that are active only in combination with other enhancers, or low-activity enhancers below the detection limit of the assay. During ENCODE Phase IV, we continue to evaluate the accuracy of cCREs using the data generated by ENCODE Functional Characterization Centers with massively parallel reporter assays, STARR-seq, various flavors of CRISPR assays, and transgenic mouse assays.

Supplementary Note 12. Comparing cCREs with active regions identified by high-throughput reporter assays

To further evaluate evidence for activity of cCREs, we compared the cCREs defined in two cell lines (GM12878 and K562) with public data on regions tested using two different types of high-throughput reporter assays in the respective cell types[50,51]. In both comparisons, regions that overlapped cCREs annotated in the corresponding cell type validated at a higher rate than the regions that did not overlap cCREs.

Tewhey et al.[50] tested 25,295 regions that contained variants from cis-expression quantitative trait loci (eQTL) identified in human lymphoblastoid cell lines (LCLs). They performed massively parallel reporter assays (MPRA) on these regions containing either allele of the variants, and 3,103 of the regions were deemed active for one or both alleles in GM12878 cells (also known as NA12878 in the 1000 Genomes Project). Active regions (MPRA+) were significantly more likely to overlap GM12878 cCREs than inactive regions (22.0% vs. 3.9%; at least 25% bps of the cCRE needs to overlap, Fisher's exact *p*-value = 2.5E-235). DNase signals of the tested cCREs in GM12878 were predictive of their MPRA activities: 12 of the top 1,000 ranked cCREs in GM12878 overlapped an MPRA-tested region, and 11 of these were MPRA+ (MPRA test positivity = 91.7%). The test positivity was 62.5% and 53.2% for the top 3,000 or 5,000 cCREs, for which 48 and 79 cCREs overlapped tested regions respectively. The overall test positivity for all cCREs annotated in GM12878 (N = 103,021, from 103,195 GRCh38 lifted down to hg19 to compare with the MPRA data in hg19) was 44.0% (1,372 GM12878 cCREs overlapped a tested region), more than thrice

higher than the overall validation rate of all tested regions (12.3%). The overall test positivity for cCREs-PLS was 62.6% and cCREs-ELS was 33.6% (39.8% for cCREs-dELS and 28.8% for cCREs-pELS; **Fig. 4c**).

Notably, cCREs-dELS (TSS-distal cCREs with enhancer-like signatures) with high epigenetic signals in LCLs were more predictive of MPRA activity than cCREs-dELS in other lymphoid biosamples (Wilcoxon rank-sum test p = 1.0E-3), which were in turn more predictive than cCREs-dELS in non-lymphoid cell types (p = 1.1E-13; the three rightmost bars in **Supplementary Fig. 15f**). However, the opposite trend was observed for PLS (promoter-like signature), pELS (TSS-proximal with enhancer-like signatures), and for all high-DNase cCREs in general, i.e., these three groups of cCREs in lymphoid biosamples were less predictive of MPRA activity than were the corresponding groups of cCREs in other biosamples (**Supplementary Fig. 15f**, the 9 bars from left, in three sets), which seems counterintuitive.

Further analysis revealed that the counterintuitive trends observed for cCREs-PLS, cCREs-pELS, and high-DNase cCREs were due to the ascertainment bias of the 25,295 tested regions—they were chosen from eQTLs in LCLs[50]. cCREs that are specific for non-lymphoid biosamples are unlikely to overlap these tested regions, while those cCREs in non-lymphoid biosamples that do overlap these regions are likely active across many biosamples, and such ubiquitous cCREs are expected to validate at a higher rate than cell type-specific cCREs. Indeed, substantially fewer cCREs-PLS in non-lymphoid biosamples than in lymphoid biosamples overlapped the tested regions, and the number of overlapping cCREs-PLS were nearly perfectly anti-correlated with the percentage of these regions that tested positive (Pearson's correlation r = −0.91, p = 1.4E−203; **Supplementary Fig. 15g**). A similarly strong anticorrelation was observed for cCREs-pELS and high-DNase cCREs (r = −0.80 and −0.85, p = 2.8E−117 and 8.9E−143). In sharp contrast, such a correlation was absent for cCREs-dELS (r = 0.04, p=0.3), although ~54% more cCREs-dELS in lymphoid biosamples than in non-lymphoid biosamples overlapped the tested regions. The lack of a correlation for cCREs-dELS is because few dELS are ubiquitously active (**Supplementary Fig. 3d**). The above detailed analysis reveals that one must be cautious when performing cross-cell type comparisons of a genome-wide set of annotations (0.9 M cCREs in this case) with another set that is chosen in a specific cell type (the 25,295 regions picked from LCL eQTLs).

The other high-throughput reporter assay that we compared human cCREs with was the SuRE assay[51], which tested 100 million 0.2–2 kb DNA fragments across the entire human genome for

their autonomous promoter activities in K562 cells. cCREs-PLS defined in K562 were validated at 73% by base, higher than pELS (69%) and dELS (46%) defined in K562, and all these rates are much higher than the background rate of 4% (**Fig. 4d**). Moreover, for all these groups of cCREs, those defined in K562 were validated at a significantly higher rate than cCREs defined in other myeloid biosamples, which were, in turn, validated at higher rates than cCREs defined in other biosamples (**Supplementary Fig. 15h**). Thus, the cCREs defined by epigenetic signals in specific cell types are consistent with their activities in the corresponding cell types.

Considering both the transgenic mouse assay results in the previous section and the reporter assay overlaps in this section, we estimate the test positivity to be ~65% for top 1000 cCREs in each biosample. The test positivity is 20-40% for all cCREs, which is still well above background. The failure of some cCREs to test positively likely reflects both the inherent limitations of our biochemical signal-based approach and also limitations of transgenic mouse assays and reporter assays. Overall, the assays do not test cCREs in their native chromosomal context: (i) the reporter assays did not test DNA segments with their native target promoters, and promoter choice is known to affect the sensitivity and cell type specificity; (ii) the reporter assays do not test combinatorics, and some cCREs may be active only when combined with others, even much further away; (iii) other aspects of native epigenomic and topological context are not recapitulated in reporter constructs; and (iv) the short MPRA-tested regions, in particular, may not contain the full sequence required for enhancer activity, while the longer transgenic segments might even include unintended repressing elements. On the other hand, we also acknowledge the possibility that not all open chromatin regions marked by high levels of H3K27ac function as enhancers; therefore these regions will not test positive in functional characterization experiments. Ongoing work in Phase IV of ENCODE by our Functional Characterization Centers, as well as work in the wider community, are using CRISPR-based assays that can maintain native chromosomal context[96] and comparing them with other assays to better illuminate the full range of regulatory activity in cCREs.

Supplementary Note 13. Using the Registry of cCREs and SCREEN for interpreting GWAS variants.

Most variants discovered in genome-wide association studies (GWAS) lie outside of coding regions and are enriched in known or suspected regulatory regions[1,29,30,95,97,98]. With the broad coverage of biosamples and rich epigenetic and transcription factor binding data associated with the cCREs, all made available via a web-based resource called SCREEN (Search Candidate cis-

Regulatory Elements by ENCODE; http://screen.encodeproject.org, **Box 2**), the Registry of cCREs can be particularly useful for annotating GWAS variants. We have analyzed 3,751 GWAS from the NHGRI-EBI catalog[99,100], surveying 2,321 phenotypes (**Supplementary Table 23**). We first determined which tag single-nucleotide polymorphisms (SNPs) from these studies and their neighboring SNPs in linkage disequilibrium (LD) were located in the cCREs predicted to be active in each biosample by epigenetic signals, and then used this information to suggest candidate regulatory functions for the SNPs. For the GWAS with 25 or more SNPs in a human population with LD data, we further identified the biosamples with significant enrichment of cCREs overlapping these SNPs (**Supplementary Fig. 16a-c**). Here, we delve into several GWAS to test how cCREs and SCREEN can aid exploratory analyses of annotating GWAS SNPs.

The first SNP we investigated, rs1250568, is in LD with three tag SNPs—rs1250542[101], rs1250540[102], and rs1782645[103]—of several GWAS on multiple sclerosis. SNPs reported in these GWAS are enriched for cCREs with high epigenetic signals in T cells and B cells as well as in the lymphoblastoid cell line GM12878, which has substantial ENCODE data (**Supplementary Fig. 16c**, left panel). rs1250568 lies in cCRE-PLS EH38E1482633 (**Supplementary Fig. 16d**), and data in GM12878 reveal that the SNP resides in a ChIP-seq peak of the transcription factor ELF1, likely disrupting an ELF1 motif site (**Supplementary Fig. 16e**). ELF1 is primarily expressed in lymphoid cells and is involved in the IL-2 and IL-23 immune response pathways; both pathways are implicated in multiple sclerosis[104,105]. RNA Pol II ChIA-PET data in GM12878 link EH38E1482633 with *ZMIZ1*, the gene containing rs1250568 within an intron, and *PPIF*, a downstream gene (**Supplementary Fig. 16f**). *ZMIZ1* was proposed to be a causal gene in the GWAS[101,102]—it is in the androgen receptor signaling pathway and is expressed at lower levels in multiple sclerosis patients than in controls[106]. While *PPIF* has not been implicated in multiple sclerosis, our exploratory analysis suggests that it might be. It encodes a peptidyl-prolyl cis-trans isomerase, which is a component of the mitochondrial permeability transition pore. Knockdown or knockout of *Ppif* led to neuroprotective effects in mouse disease models of multiple sclerosis[107,108]. We propose that *PPIF* may function in lymphocytes to promote demyelination of neighboring neurons.

The second GWAS example contains 75 SNPs significantly associated with red blood cell phenotypes[109]. These SNPs fall into 45 LD blocks ($r^2 \geq 0.7$) with 91% of the blocks containing at least one SNP that overlaps a cCRE, and these cCREs have high H3K27ac signals in blood cells, particularly in K562, an erythroleukemia cell line, reproducing previous reports on the K562

enrichment[2,110]. Ulirsch *et al*. performed MPRA on K562 cells to functionally characterize 2,756 SNPs in LD with these 75 SNPs and validated 32 of them (referred to as MPRA functional variants, MFVs). They noted that 28% of these 32 MFV+ SNPs overlap with K562 DHSs[110]. We observed that 47% of the MFV+ SNPs overlap cCREs, while only 15% of the MFV− SNPs did (3.1-fold enrichment; p = 2.4E-5), and the enrichment was even higher for cCREs predicted to be active in K562—22% vs. 4% (5.0-fold enrichment; p = 4.3E-4; **Supplementary Fig. 17a**). Ulirsch et al. further validated three of the 32 MFV+ SNPs using CRISPR/Cas9 to create isogenic clonal deletions across each MFV (median size was 13 nucleotides) in K562 cells[110]. Two of these SNPs, rs1175550 and rs1546723, overlap cCREs-PLS in blood cell types, while the other SNP, rs737092, overlaps a cCRE-ELS (EH38E2124446; **Supplementary Fig. 17b**). Ulirsch et al. found that rs737092 affects the expression of a gene *RBM38* coding an RNA-binding protein, whose TSS is 22.7 kb away. Consistent with this result, EH38E2124446 shows high H3K27ac and DNase signals in K562 and other blood cell types (**Supplementary Fig. 17c**). Its homologous mouse cCRE, EM10E0721638, also has high DNase and H3K27ac signals in blood cell types and fetal liver (**Supplementary Fig. 17d**)—liver is a major contributor of hematopoiesis during development[111]. Promoter-capture Hi-C (CHi-C) data linked rs737092 with *RBM38* in CD34+ hematopoietic progenitor cells[112], as noted by Ulirsch et al. We capture this cCRE-gene link for EH38E2124446 in SCREEN.

The third example involves an SNP (rs2742624) that was identified by integrative analysis of genomic data (DNase-seq, ChIP-seq of H3K27ac and transcription factors, and expression quantitative trait loci or eQTL) on the prostate cancer cell line LNCaP and subsequently validated by transient transfection and CRISPR/Cas9 deletion of a 168-bp region containing the SNP in the same cell line[113]. The CRISPR/Cas9 data revealed that rs2742624 modulated the expression of *UPK3A*, a gene 3,984 bp away[113]. SCREEN shows that rs2742624 overlaps EH38E2169396, a cCRE-ELS with the highest DNase Z-score in LNCaP cells (**Supplementary Fig. 18a-c**). There are no ENCODE H3K27ac ChIP-seq data on LNCaP, but this cCRE-ELS has a high H3K27ac signal in PC-3 and vCaP, two other prostate cancer cell lines. SCREEN further shows eQTL data linking EH38E2169396 to *UPK3A* in multiple tissues, including prostate (**Supplementary Fig. 18d**), consistent with the CRISPR/Cas9 results of Jin et al. *UPK3A* encodes uroplakin 3A, a group of transmembrane proteins that form a highly specialized biomembrane in terminally differentiated urothelial cells. Additional eQTL and Hi-C data in SCREEN link EH38E2169396 to two other genes *FAM118A* and *FBLN1*, providing ideas for additional experiments. Thus, SCREEN can

expedite the integrative analyses that prioritize SNPs for further experimental testing without requiring the user to write any computer programs.

The fourth example examines a SNP (rs12740374) associated with 18 phenotypes (as a lead SNP for three phenotypes and in LD with a lead SNP for 15 phenotypes), including cholesterol levels, coronary diseases, and metabolite levels (**Supplementary Table 23b**). The SNP overlaps EH38E1374646, a cCRE-ELS with high epigenetic signals across many biosamples, including hepatocytes, brain tissue, intestinal tissue, and stem cells (63/136 H3K27ac and 209/462 DNase experiments, **Supplementary Fig. 19a-c**). Warren et al. demonstrated through iPSC differentiation and CRISPR/Cas9 assays that this region controls different genes depending on the tissue context[114]. SCREEN shows ChIA-PET interactions and eQTLs between this cCRE-ELS and multiple genes, including *CELSR2*, *PSRC1*, and *SORT1*, corroborating previous results of functional testing (**Supplementary Fig. 19d**).

In our final example, SNP rs13025591 has been reported by two studies to be associated with schizophrenia (p = 8E-8 and 6E-6)[115,116]. rs13025591 does not lie within a cCRE; however, it is located in an LD block that contains three other SNPs and six cCREs (**Supplementary Fig. 20a**) in three introns of the *AGAP1* gene, which is highly expressed in neurospheres, spinal cord, and mouse fetal brain regions (**Supplementary Fig. 20b**). The SNPs in this LD block are associated with cognitive and neural phenotypes, including educational attainment, cognitive performance, anxiety disorder, and schizophrenia[99,100]. An adjacent cCRE (EH38E2086160) has high H3K27ac signals in neuronal cells and high DNase signals in fetal brain tissues and eye, but less so in other tissues, and they displayed signatures of candidate enhancers in neural cells (**Supplementary Fig. 20a**, with mini-peaks displayed by the Signal Profile tool of SCREEN). The signal strength of the DHSs in the brain appears to decline with fetal age. We then turned to the orthologous *Agap1* locus in mouse, which has three cCREs defined in the brain (**Supplementary Fig. 20c**), for a systematic study of the variation of signal strength with fetal age. Across 12 tissues at eight timepoints of fetal development, one of the mouse cCREs-dELS, EM10E0042440, has the highest H3K27ac signals in brain regions (**Supplementary Fig. 20d**), and the mouse *Agap1* gene is predominantly expressed in fetal brain tissues (**Supplementary Fig. 20e**). EM10E0042440's H3K27ac signals in the forebrain, midbrain, and hindbrain increased over time, reaching a maximum on e13.5. Then, similar to the homologous human cCRE, H3K27ac signals at the mouse cCRE decreased after e13.5 through birth (**Supplementary Fig. 20f**). These results suggest that this cCRE is active during a narrow window of brain development. We tested this

cCRE-dELS using e12.5 transgenic mouse assays and found that it showed enhancer activity exclusively in the central nervous system (**Supplementary Fig. 20g**).

These five examples illustrate that the Registry of cCREs can aid the exploration of diverse biochemical data in hundreds of human and mouse biosamples and facilitate the generation of scientific hypotheses to guide further experimentation. The last example highlights the particular strength of the Registry in its inclusion of both human and mouse cCREs and the definition of homologous cCREs between these two species. ENCODE has extensive data on mouse tissues during fetal development; this is particularly valuable because human developmental tissues are impractical to obtain. Thus the homologous mouse cCREs can complement the human cCREs in applications, such as interpreting GWAS variants associated with developmental diseases, especially those that affect the brain. SCREEN enables users to identify the biosamples that are likely implicated in diseases, explore possible mechanisms by which cCREs and SNPs may cause the disease, and prioritize new SNPs and identify novel disease-linked regions for further testing. These examples place the Registry and SCREEN in the greater context of ENCODE data and illustrate the utility of the ENCODE resource for studying mammalian biology.

Supplementary Methods

Analysis to support the choice of DNase, H3K4me3, and H3K27ac signals for defining cCREs

Testing single features for predicting VISTA enhancers

We downloaded all regions from the VISTA Enhancer database in November 2015. Merging overlapping regions yielded 1,994 unique regions. Because we had histone mark ChIP-seq, DNase-seq, and RNA-seq data for the hindbrain, limb, midbrain, and neural tube at embryonic day 11.5, we used the VISTA regions active in these four tissues at e11.5 for testing the epigenetic signals. There were 301, 271, 193, and 228 active regions in the midbrain, hindbrain, neural tube, and limb, respectively (**Supplementary Table 12**). To aid the clarity of our description, we use VISTA regions to include both positive and negative regions and VISTA enhancers to mean only those positive regions.

We first determined the best method for anchoring enhancer predictions, i.e., whether to center the genomic regions on DNase hypersensitive sites (DHSs), H3K27ac peaks, H3K9ac peaks, H3K4me3 peaks, H3K4me2 peaks, or H3K4me1 peaks as predicted enhancers, ranked by their corresponding signals. We did not test H3K36me3, H3K27me3, H3K9me3, or DNA methylation for anchoring enhancer predictions because these signals are too diffuse. Because DHSs and histone mark peaks have different widths, we standardized comparisons by resizing all peaks to the same width of 300 base pairs, centered on the midpoint of DHSs or the summit of histone peaks (the position in the peak with the highest ChIP signal), and used these 300-bp regions as enhancer predictions.

We intersected predicted enhancers anchored on DHSs or histone mark peaks with all VISTA regions. If a VISTA region overlapped a predicted enhancer by at least 1 bp, we assigned the region the score of the DHS or ChIP-seq peak, i.e., its average signal across the DHS or peak. If a VISTA region overlapped multiple DHSs or peaks, we assigned it the maximal score of the overlapping DHSs or peaks. If a VISTA region did not overlap any DHSs or peaks, we assigned it a minimal score of 0. To evaluate the performance of each method, we calculated the area under the Precision-Recall curve (AUPR). Precision is defined as the percentage of predictions that are true positives (i.e., active VISTA regions in a tissue). Recall, which is the same as sensitivity, is the percentage of true positives that are predicted as positives. To plot a PR curve, we first ranked all VISTA regions (1,994 regions in total) from the highest score to the lowest

score. We then stepped down this ranked list and computed the precision and recall for the VISTA regions above each rank and drew this pair of precision-recall values on the graph. The beginning of a PR curve can be spiky because only the top few predictions are included in the precision and recall calculations. Because not all VISTA enhancers overlap a DHS or a histone mark peak (nor are they expected to overlap based on the diverse ways that VISTA elements have been selected over time), the PR curves stop at various positions before they reach the 100% recall. The area under a PR curve is computed until its stopping point.

Averaged over the four tissues, DHSs performed the best as the anchor for enhancer predictions, followed by H3K27ac peaks as the second-best choice as the anchor (**Supplementary Fig. 1a, Supplementary Table 13a**). Another reason that DHSs are a better choice than H3K27ac for anchoring enhancer predictions is that there are more DHSs than H3K27ac peaks called for each tissue.

We then tested different metrics for ranking all 300-bp regions anchored on DHSs: DNase signal (averaged over the ± 250 bp window centered on the DHS mid-point), each of the aforementioned eight types of histone marks (averaged over the ± 1 kb window centered on a DHS mid-point or centered on the summit of a ChIP-seq summit), and DNA methylation (averaged over the ± 1 kb window centered on a DHS mid-point or centered on the summit of a ChIP-seq summit). Again, the DNase signal was better than the H3K27ac signal (average AUPR = 0.38 and 0.34, respectively), and they were far better than the other epigenetic signals (**Supplementary Table 13b**). We further tested the average rank of DNase and each of the other nine epigenetic signals, i.e., we obtained the rank of DNase signal and the rank of another epigenetic signal for each 300-bp window anchored on a DHS and then averaged the two ranks and used the average rank as the metric. Averaged over the four tissues, the best performing metric was the average rank of H3K27ac and DNase signals (**Supplementary Fig. 1b**, **Supplementary Table 13b**).

Prediction of expression levels using TSS-proximal DNase and histone mark signals

To test methods of promoter prediction, we used transcript expression values from the ENCODE RNA-seq uniform processing pipeline. We computed Pearson correlations between the ranks of TSS-proximal (± 2 kb) DHSs or H3K4me3 peaks (by DNase signal or H3K4me3 signal) and the ranks of the expression levels of nearby transcripts (**Supplementary Fig. 1c**, **Supplementary Table 14**). We tested all four combinations of ranking schemes (DHSs ranked by DNase signal, H3K4me3 peaks ranked by DNase signal, DHSs ranked by H3K4me3 signal, and H3K4me3

peaks ranked by H3K4me3 signal) for mouse tissues (e11.5) and human cell lines GM127878, K562, and HepG2. The method with the highest correlation was anchoring predictions on DHSs and ranking by the H3K4me3 signal.

Identification and classification of cCREs

DNase-seq data curation

We used all DNase-seq datasets (defined as individual replicates of DNase-seq experiments) available on the ENCODE portal as of September 1, 2018, with SPOT scores > 0.3 (**Supplementary Table 9c, h**). For each dataset, we specifically downloaded from the ENCODE Portal "enrichment" files, which were bed files containing HOTSPOT false discovery rate (FDR) scores across all positions in the entire human or mouse genome.

Calling DNase peaks

For each DNase-seq enrichment file, we called peaks using an iterative thresholding method. We used this iterative peak calling method because deeply sequenced datasets globally have more significant FDRs and, as a result, calling peaks at one FDR threshold would lead to wider peaks in more deeply sequenced datasets. Starting with an FDR of 1E-2, we filtered out positions in the enrichment files that were above this significance cutoff. Using bedtools merge[117], we then merged adjacent regions to create DNase-peaks. We discarded all regions shorter than 50 bp. We repeated the process 4940 times, each time making the FDR more stringent by a factor of 10 (i.e., 1E-3, 1E-4, 1E-5, etc.) until we reached 1E-4942, the computational limit for long doubles. Once we called peaks at these 4940 thresholds, we combined peaks as follows. Starting at 1E-2, we retained all peaks between 50 bp and 350 bp. Then at the next threshold (1E-3), we retained all peaks between 50 and 350 bp that did not overlap any of the previously retained peaks. We repeated this process until the 1E-4942 threshold, resulting in a list of unique DNase peaks between 50 bp and 350 bp in width.

Filtering DNase peaks

For each DNase-seq dataset, we calculated the average DNase signal (sequence-depth normalized as the number of reads per one million total reads, or RPM) across the positions in each peak. We then retained all DNase peaks that were: (i) between 150 and 350 bp in width, (ii) had an FDR below 1E-3, (iii) had a signal higher than the 10th percentile among the peaks in all DNase-seq datasets in each species. This 10th percentile (0.1508 RPM for GRCh38 and 0.1454

RPM for mm10) was calculated from 100,000 randomly selected DNase peaks. After this filtering, we retained 65 million (70.5%) GRCh38 and 13 million (66.5%) mm10 peaks.

Merging DNase peaks to define rDHSs

Using bedtools merge[117], we clustered DNase peaks across all DNase-seq experiments. For each cluster of DNase peaks, we selected the peak with the highest signal (normalized by sequencing depth of the respective sample) as the representative DNase hypersensitive site (rDHS) for the cluster. All DNase peaks that overlapped with this rDHS by at least one bp were considered as being already represented by the rDHS and removed from future rounds of clustering. We then repeated the clustering using the remaining DNase peaks, once again identifying the peak with the highest signal as the rDHS to represent each cluster and removing all the overlapping DNase peaks. We repeated this process until it finally resulted in a list of non-overlapping rDHSs representing all DNase peaks in all samples, totaling 2.27 and 1.23 million rDHSs in human (GRCh38) and mouse (mm10), respectively.

Further filtering of rDHSs by comparing with cDHSs

To reduce the number of false positives, we performed one last filtering step using a list of independently derived consensus DHSs (cDHSs) generated by the Stamatoyannopoulos laboratory. Using bedtools intersect[117], we intersect the list of rDHSs and the list of cDHSs. We retained rDHSs if they met the following criteria: (i) contained an entire cDHS, (ii) overlapped a cDHS by at least 135 bp, or (iii) overlapped a cDHS by at least 1 bp and was at the 90th percentile for DNase signal. Both the 135 bp cutoff and the 90th percentile signal cutoff were determined by identifying the inflection points on overlap curves. This filtering results in 2.2 million (97%) GRCh38 and 1.2 million (97%) mm10 rDHSs.

Assigning cCREs to Tiers

Once we completed the cell type-agnostic state and group classifications of cCREs (**Methods**, **Box 1, Supplementary Fig. 2**), we had the pertinent mark for each cCRE in each biosample (i.e., H4K3me3 for PLS and DNase-H3K4me3, H3K27ac for ELS, and CTCF for CTCF-only cCREs), for concordancy testing and Tier classification. To perform the concordancy test, we examined whether the high DNase signal and the high signal of the pertinent mark of each cCRE were concordantly from the same biosample, taking into account missing data in those biosamples not completely covered by the four core assays.

We use a cCRE with a cell type-agnostic group classification of PLS to illustrate the steps of the contingency test—the pertinent signal for this cCRE would be H3K4me3. If this cCRE has a high DNase signal and a high H3K4me3 signal from the same biosample, then it passes the concordancy test and is deemed a Tier 1 cCRE. There may be multiple biosamples in which this cCRE has high signals for both DNase and H3K4me3. If at least one of the biosamples is covered by all four core assays, then this cCRE is in Tier 1a (**Supplementary Fig. 3a-c**); otherwise, it is in Tier 1b. Alternatively, if the high DNase signal and a high H3K4me3 signal for this cCRE are never in the same biosample, there may be multiple reasons. The concordancy test cannot be performed on the cCRE if all the biosamples in which it has a high DNase signal do not have H3K4me3 ChIP-seq data and all the biosamples in which it has a high H4K3me3 signal do not have DNase-seq data; the reason is missing data, and the cCRE is classified as Tier 2. Another scenario is that the concordancy test could be performed on the cCRE, and it failed for some biosamples but could not be performed in other biosamples due to missing data. For example, if the cCRE has a high DNase signal and a low H3K4me3 signal in all of the biosamples with both DNase-seq and H3K4me3 ChIP-seq data, and furthermore, the cCRE has a high H3K4me3 signal in other biosamples with H3K4me3 ChIP-seq data but no DNase-seq data, then the cCRE failed the concordancy test for the former set of biosamples but could not be tested for the latter set of samples. Such cCREs are classified as Tier 3. If the missing DNase-seq assay can be performed on the latter set of samples in the future and the cCRE gets a high DNase score, then the cCRE would be reclassified as a Tier 1 cCRE.

The above cCRE-PLS example can be extended to other cCRE groups. To facilitate the enumeration of all possible scenarios of missing data, we grouped biosamples into four types (**Supplementary Fig. 5**): Type A samples were covered by all four assays, Type B samples had data from DNase-seq and at least one of the ChIP-seq assays, Type C samples only had ChIP-seq data, and Type D samples only had DNase-seq data. Tier 1a and 1b cCREs can only be from Type A or Type B samples, respectively (**Supplementary Fig. 5**). Tier 2 cCREs come from a combination of a Type C biosample with a Type D or B biosample, and all possible combinations of biosample types are illustrated in **Supplementary Fig. 5**.

To compare group classification between different biosamples, we extracted cell type-specific cCREs for biosamples with all four core assays (25 for human and 15 for mouse). For the three cell type-agnostic groups PLS, pELS, and dELS, we calculated the percentage of cCREs that were classified in a different group in at least one biosample. For example, we selected all cell

type-agnostic cCREs-PLS, then looked at their classifications across the 25 human biosamples. If they were only ever classified as PLS, DNase-only, or low-DNase, they were assigned to the red majority group. If they were ever classified as pELS, even in just one of the 25 biosamples, they were assigned to the orange pELS group. cCREs that switch to multiple groups in different biosamples were denoted in black, e.g., a cell type-agnostic PLS that became pELS in one biosample and CTCF-only in another biosample. To visualize this group switching, we selected two cCREs-ELS that switch between DNase-H3K4me3 and ELS (**Extended Data Fig. 1c**) and between ELS and CTCF-only (**Extended Data Fig. 1d**).

Total genomic coverage of cCREs

To determine the genomic coverage of GRCh38 and mm10 cCREs (**Supplementary Fig. 2c, h**), we first calculated the total number of basepairs occupied by each group of cCREs, then divided it by the respective mappable genome. We defined the GRCh38 and mm10 mappable genomes as follows. For GRCh38, we calculated the total length of the genome using chromosomes from http://hgdownload.cse.ucsc.edu/goldenPath/hg38/bigZips/hg38.chrom.sizes (3,209,458,105 bp) and then subtracted out "blacklisted regions" as defined in "wgEncodeDacMapabilityConsensusExcludable.bed" (in ENCFF163ZHO), resulting in a mappable genome size of 3,209,441,065 bp. For mm10, we calculated the total length of the genome using chromosomes from https://hgdownload-test.gi.ucsc.edu/goldenPath/mm10/bigZips/mm10.chrom.sizes (2,730,871,774 bp) and then subtracted out blacklisted regions in "mm10.blacklist.bed" (in ENCSR681OIW), resulting in a mappable genome size of 2,730,790,314 bp.

Contributions from ENCODE Phases II and III and Roadmap to rDHSs and cCREs

To estimate the contributions of ENCODE and Roadmap data for making rDHSs, we randomly sub-sampled DNase datasets and counted the number of resulting rDHSs (**Supplementary Fig. 6a**). Specifically, we randomly selected n biosamples, where n is between 25–525 in bins of 25. We then selected all corresponding DHSs for these biosamples (including their biological replicates) and calculated the number of resulting rDHSs using the rDHS selection pipeline described above. We performed the selection for 100 times for each bin. We also calculated the number of resulting rDHSs using ENCODE Phase II DNase-seq data only, Roadmap DNase-seq data only, and ENCODE Phase II plus Roadmap DNase-seq data (dots in **Supplementary Fig. 6a**). Furthermore, we partitioned the cCREs that resulted from each consortium (**Supplementary Fig. 6b**) by biosample type (**Supplementary Fig. 6c**).

cCRE coverage of H3K4me3, H3K27ac, and CTCF ChIP-seq peaks in biosamples without DNase-seq data

To determine the comprehensiveness of the Registry, we overlapped cCREs with ChIP-seq peaks (H3K4me3, H3K27ac, and CTCF) from biosamples lacking DNase data (**Supplementary Fig. 7**). Using bedtools merge[117], we merged all ChIP-seq peaks within 200 bp of one another and assigned each merged peak the maximal -log(FDR) score of the contributing peaks. We then filtered out all peaks with $-\log_{10}$(FDR) < 2. Using bedtools intersect[117] with the "-u" flag, we intersected the merged peaks with cCREs and counted the number of unique peaks that overlapped at least one cCRE. To test if the low overlap for a ChIP-seq dataset was linked with lower quality peaks, we plotted the percent overlap with cCREs vs. the average peak $-\log_{10}$(FDR) for each ChIP-seq experiment.

Overlap of cCREs with ChromHMM states

We compared cCREs to the chromatin states called by ChromHMM in both human[29] and mouse[84]. For human, we analyzed the ChromHMM regions for GM12878 cells (ENCFF001TDH) and lifted the GRCh38 cCREs down to the hg19 genome. For mouse, we analyzed 15 combinations of tissue and developmental time points (e11.5 and e14.5) for which we had DNase, H3K4me3, and H3K27ac data. We overlapped PLS, pELS, dELS, and DNase-H3K4me3 cCREs with ChromHMM states derived from eight histone marks in the same tissue at the same time point.

We combined similar ChromHMM states in human to generate seven broad states: active promoter (state 1) and weak promoter (state 2) are combined into *TSS*; poised promoter (state 3) corresponds to *TSS bivalent*; strong enhancer (states 4 and 5) are combined into *high-signal enhancer*; weak enhancer (states 6 and 7) are combined into *low-signal enhancer*; insulator (state 8) corresponds to *insulator*; transcription transition (state 9), transcription elongation (state 10), and weak transcription (state 11) are combined into *transcription*; repressed (state 12), heterochromatin low (state 13), repetitive/CNV (state 14; CNV: copy number variation), and repetitive/CNV (state 15) are combined into *repressed*. For the mouse, we combined similar ChromHMM states in human to generate six broad states: active TSS (TssA) and flanking TSS states (TssAFlnk1 and TssAFlnk2) are combined into *TSS*; TSS bivalent (TSSBiv) corresponds to *TSS bivalent*; enhancer (Enh) and weak enhancers (EnhWk1 and EnhWk2) are combined into *high-signal enhancer*; poised enhancers (EnhPois1 and EnhPois2) are combined into *low-signal*

enhancer; transcription and weak transcription (Tx1, Tx2, and TxWk) are combined into *transcription*; heterochromatin and quiescent states (HetFac, HetCons, Quies) are combined into *repressed*.

To determine the ChromHMM states of cCREs, we intersected each cCRE with all ChromHMM states using bedtools[117] and selected the state that overlapped the largest number of base pairs; i.e., each cCRE was assigned to its majority ChromHMM state. We overlapped the GM12878 cCREs with the ChromHMM states calculated the majority state for each cCRE (**Supplementary Fig. 8a**). For cCRE-pELS, we observed an enrichment in the ChromHMM TSS state. To test whether this enrichment was caused by the low spatial resolution of the ChromHMM TSS state, we plotted the percentage of pELS that fell into each ChromHMM state as a function of distance from the nearest transcription start sites (TSSs) annotated by GENCODE (**Supplementary Fig. 8b**). We also calculated the percentage of each mouse cCRE with the ChromHMM states annotated in the same tissue and time point (**Supplementary Fig. 8c**).

For both human and mouse, we also performed the complementary analysis where we analyzed what percentage of each ChromHMM state overlapped a cCRE. We analyzed the overlap in two ways. First, we calculated the percentage of individual chromHMM states that overlapped a cCRE. Second, we looked at the total percentage of base pairs in each state that overlapped cCREs active in the biosample. The results are tabulated separately (**Supplementary Table 16**).

Comparison of cCREs with FANTOM enhancers

To compare cCREs-ELS with enhancer annotations of the FANTOM consortium, we downloaded the set of 65,423 permissive human FANTOM5 enhancers (http://fantom.gsc.riken.jp/5/datafiles/latest/extra/Enhancers/human_permissive_enhancers_phase_1_and_2.bed.gz), which we lifted up to the GRCh38 genome using UCSC liftOver (N=65,407). We then intersected the FANTOM enhancers with cCREs-dELS using bedtools intersect[117] with default parameters. We plotted histograms of DNase, H3K4me3, H3K27ac, H3K4me1, and Pol II max-Z signal between cCREs-ELS that overlap FANTOM enhancers, and those that did not (**Supplementary Fig. 9a-e**).

To compare against a wider set of FANTOM annotations, we downloaded a set of 473,134 TSS annotations from the FANTOM CAGE associated transcriptome (CAT) project

(http://fantom.gsc.riken.jp/5/suppl/Hon_et_al_2016/data/assembly/lv3_robust/FANTOM_CAT.lv3_robust.info_table.ID_mapping.tsv.gz). Because of the single-nucleotide resolution of these FANTOM TSSs, we lifted the GRCh38 cCREs down to the hg19 genome before applying bedtools intersect[117] (default parameters). Of the FANTOM TSSs that overlapped cCREs, we calculated the percentage that overlapped PLS, pELS, or dELS stratified by the RNA annotation (11 types) provided by FANTOM CAT project (**Supplementary Fig. 9f**).

Evolutionary conservation of cCREs

For average conservation score analysis on each set of cCREs (**Extended Data Fig. 2b**, **Supplementary Fig. 10b, c**), we calculated the average evolutionary conservation (calculated from the phyloP[69] score per genomic position from the alignment of 100 vertebrate genomes http://hgdownload.cse.ucsc.edu/goldenpath/hg38/phyloP100way/hg38.phyloP100way.bw), for cCREs by the group in the ± 250 bp window from the center of each cCRE. We analyzed conservation stratifying by cCRE group, homology with the mouse genome, and overlap with CAGE peaks (**Supplementary Fig. 10c**). For the analysis of ranked overlap with GERP++ regions (**Supplementary Fig. 10a**), we binned PLS, pELS, dELS, and CTCF-only cCREs by their DNase max-Z values (at a 0.1 interval). We then calculated the total number of bases in each bin of cCREs that overlapped a GERP++ region[85] as defined in http://mendel.stanford.edu/SidowLab/downloads/gerp/hg19.GERP_elements.tar.gz lifted up to the GRCh38 genome using UCSC liftOver[70].

Homologous human and mouse cCREs

Using UCSC's liftOver tool with a minimum match score of 0.5, we lifted GRCh38 cCREs to the mm10 genome (GRCh38-mm10 cCREs) and mm10 cCREs to the GRCh38 genome (mm10-GRCh38 cCREs) (**Extended Data Fig. 2c**). We labeled cCREs that did not map to the other genome as "No homology". For the cCREs that did map, we intersected them with cCREs in the other species (i.e., GRCh38-mm10 cCREs with mm10 cCREs and mm10-GRCh38 cCREs with GRCh38 cCREs) using bedtools intersect[117]. Those cCRE pairs that mapped to the other genome and intersected reciprocally (in both from human to mouse and from mouse to human directions) were labeled "Homology and cCRE". cCREs that lifted over but did not map to cCREs in the other species reciprocally were labeled "Homology only".

Repeat and transposon contents of cCREs

Annotations of repetitive elements were downloaded from UCSC Genome Browser (human: hg38 rmsk.txt; mouse: mm10 rmsk.txt). We considered all retrotransposons, transposons with long-terminal repeats (LTRs), short interspersed elements (SINEs), long interspersed elements (LINEs), and non-transposon repeats. We overlapped these transposons and repeats that were not within the blacklist regions with all cCREs by the group (PLS, pELS, dELS, DNase-H3K4me3, and CTCF-only, respectively), and tallied the total overlapping base pairs (**Supplementary Table 17**). *P*-values were estimated using Chi-squared tests with 1,000 base pairs counted as one observation.

Transcription factor support for the group classification of cCREs

For cCREs defined in GM12878, we calculated their average RNA Polymerase II (ENCFF600UCV), EP300 (ENCFF977NLF), and RAD21 (ENCFF916YVI) ChIP-seq signals using UCSC's bigWigAverageOverBed. We plotted the average ChIP-seq signal levels at cCREs stratified by their group classifications (**Extended Data Fig. 2d**).

Overlapping of cCREs with transcription factor ChIP-seq peaks

We downloaded *IDR conservative* peaks from the ENCODE portal for ChIP-seq experiments of chromatin-associated proteins (most of them were transcription factors)**.** For each experiment, we intersected the ChIP-seq peaks with cell type-agnostic cCREs using the bedtools intersect function[117] and calculated the percentage of ChIP-seq peaks that overlapped at least one cCRE (**Extended Data Fig. 3b, Supplementary Table 18**). For ChIP-seq experiments in GM12878, HepG2, and K562, we also calculated the overlap of ChIP-seq peaks with all high-DNase groups of cCREs defined in the corresponding cell type (**Supplementary Fig. 11a-d**).

We analyzed the extent that cCREs overlapped the ChIP-seq peaks of chromatin-associated proteins stratified by the number of bound proteins using ChIP-seq data from GM12878, K562, and HepG2 (**Supplementary Fig. 11c**). We performed the analysis in each of the cell types separately and excluded RNA Pol II and elongation factors from the analysis. We first merged the narrow peaks of all chromatin-associated proteins that passed the irreproducibility discovery rate (IDR) test using the “merge” function from bedtools[117] with a minimum of 1 bp overlap. We filtered out the resulting merged peaks that were greater than 2 kb from the downstream analysis. For the remaining merged peaks, we counted the number of proteins bound per peak and determined

the percentage of peaks that overlapped with cell type-agnostic cCREs as well as with the cCREs defined in the corresponding cell type, using bedtools intersect[117].

For the cCRE-centric analysis (**Supplementary Fig. 11d**), we intersected HepG2 cCREs with HepG2 ChIP-seq peaks. To ensure a 1:1 correspondence between ChIP-seq peak and cCREs, we performed the intersection using the summit position in each ChIP-seq peak (the position with the highest ChIP signal). For each cCRE group, we calculated the number of overlapping ChIP-seq summits.

Bidirectional transcription at cCREs

We downloaded BigWig signal files from GEO for GRO-seq in GM12878 (GEO accession GSM1480326) and PRO-seq in CD4+ T cells (GEO accession GSM1613181) for the plus and minus strands. For each cell type, we selected active cCREs-PLS (n=20,113 for GM12878 and n=17,119 for CD4+ T-cell) and cCREs-dELS (DNase Z-score >1.64, n=34,041 for GM12878 and n=30,023 for CD4+ T-cell). We grouped cCREs-PLS according to the genomic strand on which the nearest TSS resides. For each cCRE, we computed GRO-seq and PRO-seq signal density 2kb upstream to 2kb downstream of the cCRE center in non-overlapping 25-bp bins on each strand. The signal values for each bin were then averaged across all cCREs-PLS and cCREs-dELS and plotted using spline interpolation (**Supplementary Fig. 12a-d**).

To compare cell type-specific transcription profiles, we further divided the above cCREs-PLS and cCREs-dELS into three groups: those defined in GM12878 but not CD4+ T-cells (PLS n = 991; dELS n= 19,230), those defined in CD4+ T-cell but not GM12878 (PLS n=3,974, dELS n=16,867), and those defined in both GM12878 and CD4+ T-cell (PLS n=16,118, ELS n=9,319). We plotted the number of cCREs in each group that had >0 signal on both, only plus, only minus, or neither strand (**Supplementary Fig. 12e-f**).

Gene expression

For the cCRE-centric analysis (**Supplementary Fig. 11e**) we downloaded gene expression quantifications derived from RNA-seq data for cardiac muscle cells (ENCFF873UHA, ENCFF309DAN), hepatocytes (ENCFF072XSA, ENCFF491FPY), and neural progenitor cells (ENCFF663ARH, ENCFF672VVX). For each gene, we determined if at least one of its transcripts (GENCODE V24 annotations) overlapped a cell type-specific cCRE and, if so, which group. We then stratified gene expression (averaged between the two replicates, in transcripts per million,

or TPM) by cCRE group: PLS, other high-DNase groups (pELS, dELS, CTCF-only, and DNase-only), low-DNase cCRE, or no cCRE. We performed a Wilcoxon rank-sum test to determine statistical significance.

For the gene-centric analysis (**Supplementary Fig. 11f**), we selected all genes with an average expression of TPM > 1 between the two replicates for the same cell types mentioned above. Of these genes, we calculated the percent of genes with at least one TSS that overlaps a cell type-specific cCRE stratified by group classification.

For the RAMPAGE analysis (**Extended Data Fig. 3a**), we calculated the percentage of cell type-specific cCREs overlapping RAMPAGE peaks from the same cell type with >1 reads per million sequencing depth (RPM). We then calculated the median signal for the overlapping peaks and plotted it against the percent of cCREs overlapping.

Enrichment of TSS-distal cCREs-ELS near tissue-specific genes

Tissue specificity score[118] of each gene was calculated across 66 mouse datasets, each corresponding to a particular tissue at a developmental time point. In each sample, tissue-specific and housekeeping genes were defined as the expressed genes (TPM ≥ 1) with top 1,000 and bottom 1,000 tissue specificity scores, respectively. For tissues with cCREs defined using DNase, H3K4me3, and H3K27ac data, we computed the percentage of tissue-specific genes or housekeeping genes that had active cCREs-ELS within 10 kb of their TSSs, with active cCREs-pELS and cCREs-dELS defined for the corresponding sample (**Supplementary Table 20**). The *p*-values of enrichment or depletion were estimated by randomly selecting 1,000 expressed genes (TPM ≥ 1) in the corresponding sample 10,000 times. We plotted the percent of genes for each of the 23 biosamples with nearby cCREs-pELS and cCREs-dELS and performed a Wilcoxon signed-rank test to test for significance (**Supplementary Fig. 11g**).

Clustering of biosamples by cCREs-dELS

To compare the distal enhancer landscape between biosamples, we performed t-SNE clustering of human and mouse biosamples (**Extended Data Fig. 4**). We calculated the H3K27ac signal across all cCREs-dELS for human and mouse, respectively, using bigWigAverageOverBed. We then normalized these matrices by taking the log10 and using the standard scaling scikit learn package. We performed clustering analysis using the scikit learn t-SNE package with a perplexity

of 10 for both human and mouse. We determined clusters using k-means clustering, selecting the optimal number of clusters through the elbow method (**Supplementary Table 19**).

Differential gene expression analysis

We downloaded gene expression quantification results from the ENCODE Portal (**Supplementary Table 21**). To compute differentially expressed genes between all possible pairs of tissues and time points (2,145 pairs in total for 66 RNA-seq samples), we ran DESeq2[119] (version 1.14.1 with an FDR cutoff < 0.01).

Enrichment of GWAS variants in cCREs

We curated GWAS from the NHGRI-EBI Catalogue as of January 1, 2019 (**Supplementary Table 23**). We excluded studies performed on mixed populations and populations without LD information (N=29). Using the linkage disequilibrium (LD) values from HaploReg[120], which were computed using data from the 1000 Genomes Project for the corresponding super population (African, Ad Mixed American, Asian, and European), we generated LD blocks containing all SNPs with $r^2 > 0.7$ and uploaded these SNPs to SCREEN for cCRE intersection.

For studies with more than 25 lead SNPs, we performed biosample enrichment analysis. For each study, we generated a matching set of control SNPs as follows: for each SNP in the study (*p*-value < 1.0E-6) we selected a SNP on Illumina and Affymetrix SNP chips that fell within the same population-specific minor allele frequency (MAF) quartile and the same distance to TSS quartile (9,553, 39,530, and 154,279 bp demarcate the first, second, and third quartile, respectively). We repeated this process 500 times, generating 500 random control SNPs for each GWAS SNP. Then, for both GWAS and control SNPs, we retrieved all SNPs in high linkage disequilibrium (LD $r^2 > 0.7$), creating LD groups.

To assess whether the cCREs in a biosample were enriched in the GWAS SNPs, we intersected GWAS and control LD groups with cCREs with an H3K27ac Z-score > 1.64 in the biosample. To avoid overcounting, we pruned the overlaps, counting each LD group once per biosample. We modified the Uncovering Enrichment through Simulation (UES) method[121] by calculating *p*-values from Z-scores for performing statistical testing. We calculated enrichment for overlapping cCREs by comparing the GWAS LD groups with the 500 matched controls. Finally, we applied a FDR threshold of 5% to each study.

Testing cCREs-dELS using transgenic mouse assays

We used transgenic mouse assays to test a human genomic region that contains the cCRE EH38E2086160 that is in linkage disequilibrium with several SNPs associated with schizophrenia and other neural phenotypes (**Supplementary Fig. 20a**). The GRCh38 coordinates of the tested region are chr2:235,916,560-23,5918,287. The mouse transgenic assays are described in **Methods**. We obtained a positive result in brain tissues in ten out of ten e12.5 mouse embryos, three of which are shown in **Supplementary Fig. 20g**.

Codebase at Github

The scripts used to generate the Registry of cCREs and analyzing the cCREs are publicly available in the ENCODE Encyclopedia v4 GitHub repository at https://github.com/weng-lab/ENCODE-cCREs/). The scripts for generating the cCREs themselves are found in the *cCRE-Pipeline* directory; the *cCRE-Analysis* directory contains scripts for other analyses, e.g., peak intersection, ChromHMM overlap, cell type clustering, and saturation analysis.

SCREEN

In order to facilitate access to the Registry of cCREs, we have developed a web-based tool called SCREEN (Search Candidate Regulatory Elements by ENCODE), which allows users to search the ground level of the ENCODE Encyclopedia, the Registry of cCREs, and associated analyses such as GWAS enrichment. SCREEN also offers downloads of the complete human and mouse Registries of cCREs as well as downloads of subsets of cCREs active in cell types of interest. SCREEN is publicly available at http://screen.encodeproject.org and is compatible with all modern browsers and operating systems on both computers and mobile devices.

SCREEN's backend is implemented using a Postgres database, which allows for real-time searching of the hundreds of millions of annotations in the Encyclopedia by various criteria, including genomic coordinates and the IDs of associated annotations such as genes and SNPs. Users desiring programmatic access to the data may use SCREEN's API, which uses the GraphQL framework; this allows advanced users to incorporate complex filtering logic into their queries. SCREEN's user interface is implemented in JavaScript using the ReactJS framework, which has allowed us to implement interactive visualizations of a variety of data types contained within SCREEN, including snapshots of the signal profiles of regulatory elements, differential gene expression profiles across mouse developmental time points, and the genomic context of regulatory elements using a lightweight embedded genome browser. All these visualizations are

scalable vector graphics (SVG), which the user may download to share or use as figures. The downloaded plots may be imported into modern graphics programs, including Adobe Illustrator and Inkscape, for editing.

The current version of cCREs for the human GRCh38 and mouse mm10 genome assemblies is v2.0, while v1.0 is also available for the hg19 assembly. As the cCRE Registry gets updated, all cCREs will link to previous versions of the Registry within SCREEN and at the ENCODE Portal. SCREEN also includes an automatically scheduled versioning script, which runs once per quarter and snapshots the data included in SCREEN's ground level functionality at that moment. SCREEN's main page offers a browseable view of past ground level versions, so a user publishing on ground level data may cite a permanent record of the input data used for analysis.

Visualizing the ENCODE Encyclopedia via the UCSC genome browser

We provide two ways for users to visualize the data contained in the ENCODE Encyclopedia using the UCSC Genome Browser. First, for viewing data related to a collection of cell types of interest, SCREEN provides UCSC buttons, which lead to a user-configured genome browser view. The user may select a subset of the cell types with at least one of the four core epigenomic data types, rearrange the cell type order as desired using the drag-drop feature, and then visualize the corresponding data in the selected order in the UCSC genome browser. Tracks selected this way contain signals of DNase-seq, H3K4me3 ChIP-seq, H3K27ac ChIP-seq, CTCF ChIP-seq signals, and RNA-seq signal, as well as cell type-specific classifications of cCREs.

The cell-type-agnostic human and mouse cCREs are available as two default tracks at the UCSC genome browser.
Human cCREs: http://genome.ucsc.edu/cgi-bin/hgTrackUi?db=hg38&g=encodeCcreCombined
Mouse cCREs: http://genome.ucsc.edu/cgi-bin/hgTrackUi?db=mm10&g=encodeCcreCombined

For users wanting to visualize a larger set of the Encyclopedia's data, we offer three UCSC trackhubs, available at https://screen.encodeproject.org/hubs/dna/hub.txt, https://screen.encodeproject.org/hubs/rna/hub.txt, and https://screen.encodeproject.org/hubs/integrative/hub.txt.

The three hubs provide access to data from the Encyclopedia from DNA-based assays, RNA-based assays, and data used to create and analyze the Registry of cCREs, respectively. Data

types available through the trackhubs include peaks and signals for ChIP-seq, DNase-seq, ATAC-seq, and eCLIP; stranded signal, and transcription start site calls from RAMPAGE and CAGE; and stranded signal for RNA-seq. The cCRE hub provides cell type-agnostic and cell type-specific cCREs along with signal tracks for the core four marks, and RNA-seq and RAMPAGE where available. Both group and state classifications for cCREs are available.

With several thousand experiments available to browse, we have configured several different groupings of experiment signal and peak files to ease users' search for data of interest. Our primary grouping is by assay category (chromatin accessibility, histone modification, TF, etc.); these folders are then split by biosample types (tissue, cell lines, etc.) and tissue ontology to reduce the number of experiments on display. Each of these biosample type folders is then displayed in a 2D matrix, split on actual biosample. In addition, we also have files organized by biosample type first, then by actual biosample, and finally by assay. For assays such as ChIP-seq and eCLIP where a particular protein is targeted by the assay, we also offer a scheme which groups experiments first by target, then by biosample.

Companion website

All figures, extended data figures, tables, extended data tables, and supplementary tables presented in this manuscript are available at the manuscript's companion site, http://encyclopedia.encodeproject.org. Some figures and extended data figures are interactive on the companion site, providing links to examples of their reproduction on SCREEN and the UCSC Genome Browser. All tables on the companion site are sortable and searchable and may be downloaded in TSV format. The companion site also provides links to various resources related to SCREEN and the ENCODE portal, as well as other resources described in the paper. Please see the instructions on the companion site home page for more information.

Supplementary References

71. Boyle, A. P. *et al.* High-resolution mapping and characterization of open chromatin across the genome. *Cell* **132**, 311–322 (2008).
72. Barski, A. *et al.* High-resolution profiling of histone methylations in the human genome. *Cell* **129**, 823–837 (2007).
73. Mikkelsen, T. S. *et al.* Genome-wide maps of chromatin state in pluripotent and lineage-committed cells. *Nature* **448**, 553–560 (2007).
74. Heintzman, N. D. *et al.* Histone modifications at human enhancers reflect global cell-type-specific gene expression. *Nature* **459**, 108–112 (2009).
75. Rada-Iglesias, A. *et al.* A unique chromatin signature uncovers early developmental enhancers in humans. *Nature* **470**, 279–283 (2010).
76. Kim, T. H. *et al.* Analysis of the vertebrate insulator protein CTCF-binding sites in the human genome. *Cell* **128**, 1231–1245 (2007).
77. Xi, H. *et al.* Identification and characterization of cell type-specific and ubiquitous chromatin regulatory structures in the human genome. *PLoS Genet.* **3**, e136 (2007).
78. Ong, C.-T. & Corces, V. G. CTCF: an architectural protein bridging genome topology and function. *Nature Reviews Genetics* **15**, 234–246 (2014).
79. Visel, A., Minovitsky, S., Dubchak, I. & Pennacchio, L. A. VISTA Enhancer Browser–a database of tissue-specific human enhancers. *Nucleic Acids Res.* **35**, D88–92 (2007).
80. Hon, C.-C. *et al.* An atlas of human long non-coding RNAs with accurate 5′ ends. *Nature* **543**, 199–204 (2017).
81. Ernst, J. & Kellis, M. Discovery and characterization of chromatin states for systematic annotation of the human genome. *Nat. Biotechnol.* **28**, 817–825 (2010).
82. Hoffman, M. M. *et al.* Unsupervised pattern discovery in human chromatin structure through genomic segmentation. *Nat. Methods* **9**, 473–476 (2012).
83. Arner, E. *et al.* Transcribed enhancers lead waves of coordinated transcription in transitioning mammalian cells. *Science* **347**, 1010–1014 (2015).
84. van der Velde A Tsuji J Moore JE Purcaro M Pratt H Fan K Weng, Z. Chromatin States of Mouse Epigenomes During Fetal Development. *Communications Biology (in revision)*.
85. Davydov, E. V. *et al.* Identifying a High Fraction of the Human Genome to be under Selective Constraint Using GERP++. *PLoS Comput. Biol.* **6**, e1001025 (2010).
86. Schmidt, D. *et al.* Waves of Retrotransposon Expansion Remodel Genome Organization and CTCF Binding in Multiple Mammalian Lineages. *Cell* **148**, 335–348 (2012).

87. Sundaram, V. *et al.* Widespread contribution of transposable elements to the innovation of gene regulatory networks. *Genome Res.* (2014) doi:10.1101/gr.168872.113.
88. Core, L. J. *et al.* Analysis of nascent RNA identifies a unified architecture of initiation regions at mammalian promoters and enhancers. *Nat. Genet.* **46**, 1311–1320 (2014).
89. Danko, C. G. *et al.* Identification of active transcriptional regulatory elements from GRO-seq data. *Nat. Methods* **12**, 433–438 (2015).
90. Mahley, R. W. Apolipoprotein E: cholesterol transport protein with expanding role in cell biology. *Science* **240**, 622–630 (1988).
91. Mangeney, M. *et al.* Apolipoprotein-E-gene expression in rat liver during development in relation to insulin and glucagon. *Eur. J. Biochem.* **181**, 225–230 (1989).
92. Allan, C. M., Taylor, S. & Taylor, J. M. Two Hepatic Enhancers, HCR.1 and HCR.2, Coordinate the Liver Expression of the Entire Human Apolipoprotein E/C-I/C-IV/C-II Gene Cluster. *J. Biol. Chem.* **272**, 29113–29119 (1997).
93. Visel, A. *et al.* ChIP-seq accurately predicts tissue-specific activity of enhancers. *Nature* **457**, 854–858 (2009).
94. Visel, A. *et al.* A high-resolution enhancer atlas of the developing telencephalon. *Cell* **152**, 895–908 (2013).
95. Dickel, D. E. *et al.* Genome-wide compendium and functional assessment of in vivo heart enhancers. *Nat. Commun.* **7**, 12923 (2016).
96. Arnold, C. D. *et al.* Genome-wide quantitative enhancer activity maps identified by STARR-seq. *Science* **339**, 1074–1077 (2013).
97. Maurano, M. T. *et al.* Systematic localization of common disease-associated variation in regulatory DNA. *Science* **337**, 1190–1195 (2012).
98. Andersson, R. *et al.* An atlas of active enhancers across human cell types and tissues. *Nature* **507**, 455–461 (2014).
99. Hindorff, L. A. *et al.* Potential etiologic and functional implications of genome-wide association loci for human diseases and traits. *Proc. Natl. Acad. Sci. U. S. A.* **106**, 9362–9367 (2009).
100. MacArthur, J. *et al.* The new NHGRI-EBI Catalog of published genome-wide association studies (GWAS Catalog). *Nucleic Acids Res.* **45**, D896–D901 (2017).
101. Patsopoulos, N. A. *et al.* Genome-wide meta-analysis identifies novel multiple sclerosis susceptibility loci. *Ann. Neurol.* **70**, 897–912 (2011).
102. De Jager, P. L. *et al.* Meta-analysis of genome scans and replication identify CD6, IRF8 and TNFRSF1A as new multiple sclerosis susceptibility loci. *Nat. Genet.* **41**, 776–782

(2009).

103. International Multiple Sclerosis Genetics Consortium (IMSGC) *et al.* Analysis of immune-related loci identifies 48 new susceptibility variants for multiple sclerosis. *Nat. Genet.* **45**, 1353–1360 (2013).

104. Gallo, P. *et al.* On the role of interleukin-2 (IL-2) in multiple sclerosis (MS). IL-2-mediated endothelial cell activation. *Ital. J. Neurol. Sci.* **13**, 65–68 (1992).

105. Vaknin-Dembinsky, A., Balashov, K. & Weiner, H. L. IL-23 is increased in dendritic cells in multiple sclerosis and down-regulation of IL-23 by antisense oligos increases dendritic cell IL-10 production. *J. Immunol.* **176**, 7768–7774 (2006).

106. Fewings, N. L. *et al.* The autoimmune risk gene ZMIZ1 is a vitamin D responsive marker of a molecular phenotype of multiple sclerosis. *J. Autoimmun.* **78**, 57–69 (2017).

107. Forte, M. *et al.* Cyclophilin D inactivation protects axons in experimental autoimmune encephalomyelitis, an animal model of multiple sclerosis. *Proc. Natl. Acad. Sci. U. S. A.* **104**, 7558–7563 (2007).

108. Warne, J. *et al.* Selective Inhibition of the Mitochondrial Permeability Transition Pore Protects against Neurodegeneration in Experimental Multiple Sclerosis. *J. Biol. Chem.* **291**, 4356–4373 (2016).

109. van der Harst, P. *et al.* Seventy-five genetic loci influencing the human red blood cell. *Nature* **492**, 369–375 (2012).

110. Ulirsch, J. C. *et al.* Systematic Functional Dissection of Common Genetic Variation Affecting Red Blood Cell Traits. *Cell* **165**, 1530–1545 (2016).

111. Swain, A., Inoue, T., Tan, K. S., Nakanishi, Y. & Sugiyama, D. Intrinsic and extrinsic regulation of mammalian hematopoiesis in the fetal liver. *Histol. Histopathol.* **29**, 1077–1082 (2014).

112. Mifsud, B. *et al.* Mapping long-range promoter contacts in human cells with high-resolution capture Hi-C. *Nat. Genet.* **47**, 598–606 (2015).

113. Jin, H.-J., Jung, S., DebRoy, A. R. & Davuluri, R. V. Identification and validation of regulatory SNPs that modulate transcription factor chromatin binding and gene expression in prostate cancer. *Oncotarget* **7**, 54616–54626 (2016).

114. Warren, C. R. *et al.* Induced Pluripotent Stem Cell Differentiation Enables Functional Validation of GWAS Variants in Metabolic Disease. *Cell Stem Cell* **20**, 547–557.e7 (2017).

115. Schizophrenia Psychiatric Genome-Wide Association Study (GWAS) Consortium. Genome-wide association study identifies five new schizophrenia loci. *Nat. Genet.* **43**, 969–976 (2011).

116. Bergen, S. E. *et al.* Genome-wide association study in a Swedish population yields support for greater CNV and MHC involvement in schizophrenia compared with bipolar disorder. *Mol. Psychiatry* **17**, 880–886 (2012).
117. Quinlan, A. R. & Hall, I. M. BEDTools: a flexible suite of utilities for comparing genomic features. *Bioinformatics* **26**, 841–842 (2010).
118. Yanai, I. *et al.* Genome-wide midrange transcription profiles reveal expression level relationships in human tissue specification. *Bioinformatics* **21**, 650–659 (2005).
119. Love, M. I., Huber, W. & Anders, S. Moderated estimation of fold change and dispersion for RNA-seq data with DESeq2. *Genome Biol.* **15**, 31 (2014).
120. Ward, L. D. & Kellis, M. HaploReg: a resource for exploring chromatin states, conservation, and regulatory motif alterations within sets of genetically linked variants. *Nucleic Acids Res.* **40**, D930–D934 (2011).
121. Hayes, J. E. *et al.* Tissue-Specific Enrichment of Lymphoma Risk Loci in Regulatory Elements. *PLoS One* **10**, e0139360 (2015).

The ENCODE Project Consortium

Data analysis coordination (data analysis):

Jill E. Moore[1], Michael J. Purcaro[1], Henry E. Pratt[1], Barbara Wold[14], Ross C. Hardison[16], Thomas R. Gingeras[4], John A. Stamatoyannopoulos[5,6,37], Zhiping Weng[1,43,44]

Data production coordination (data production):

Charles B. Epstein[2], Noam Shoresh[2], Jessika Adrian[3], Trupti Kawli[3], Carrie A. Davis[4], Alexander Dobin[4], Rajinder Kaul[5,6], Jessica Halow[5], Eric L. Van Nostrand[7], Peter Freese[8], David U. Gorkin[9,10], Yin Shen[10,11], Yupeng He[12], Mark Mackiewicz[13], Florencia Pauli-Behn[13], Richard M. Myers[13], Bing Ren[9,10], Brenton R. Graveley[18], Len A. Pennacchio[17,29,40], Michael P. Snyder[3,41], Bradley E. Bernstein[42], Barbara Wold[14], Ross C. Hardison[16], Thomas R. Gingeras[4], John A. Stamatoyannopoulos[5,6,37]

Data analysis leads (data analysis):

Jill E. Moore[1], Michael J. Purcaro[1], Henry E. Pratt[1], Xiao-Ou Zhang[1], Shaimae I. Elhajjajy[1], Jack Huey[1], Joel Rozowsky[24], Jing Zhang[24], Manolis Kellis[2,35], Robert J. Klein[36], William S. Noble[37], Anshul Kundaje[3], Roderic Guigó[38], Mark B. Gerstein[24], Barbara Wold[14], Ross C. Hardison[16], Zhiping Weng[1,43,44]

Data production leads (data production):

Charles B. Epstein[2], Noam Shoresh[2], Jessika Adrian[3], Trupti Kawli[3], Carrie A. Davis[4], Alexander Dobin[4], Rajinder Kaul[5,6], Jessica Halow[5], Eric L. Van Nostrand[7], Peter Freese[8], David U. Gorkin[9,10], Yin Shen[10,11], Yupeng He[12], Mark Mackiewicz[13], Florencia Pauli-Behn[13], Brian A. Williams[14], Ali Mortazavi[15], Cheryl A. Keller[16], Diane E. Dickel[17], Valentina Snetkova[17], Xintao Wei[18], Xiaofeng Wang[19,20,21], Juan Carlos Rivera-Mulia[22,23], Surya B. Chhetri[13,25], Jialing Zhang[26], Alec Victorsen[27], Kevin P. White[28], Axel Visel[17,29,30], Gene W. Yeo[7], Christopher B. Burge[31], Eric Lécuyer[19,20,21], David M. Gilbert[22], Job Dekker[32], John Rinn[33], Eric M. Mendenhall[13,25], Joseph R. Ecker[12,34], Peggy J. Farnham[39], Richard M. Myers[13], Bing Ren[9,10], Brenton R. Graveley[18], Mark B. Gerstein[24], Len A. Pennacchio[17,29,40], Michael P. Snyder[3,41], Bradley E. Bernstein[42], Barbara Wold[14], Ross C. Hardison[16], Thomas R. Gingeras[4], John A. Stamatoyannopoulos[5,6,37]

Writing group:

Richard M. Myers[13], Bing Ren[9,10], Len A. Pennacchio[17,29,40], Michael P. Snyder[3,41], Barbara Wold[14], Ross C. Hardison[16], Thomas R. Gingeras[4], John A. Stamatoyannopoulos[5,6,37], Zhiping Weng[1,43,44]

Principal investigators (steering committee):

J. Michael Cherry[3], Richard M. Myers[13], Bing Ren[9,10], Brenton R. Graveley[18], Michael P. Snyder[3,41], Bradley E. Bernstein[42], Thomas R. Gingeras[4], John A. Stamatoyannopoulos[5,6,37], Zhiping Weng[1,43,44]

The Broad Institute of Harvard and MIT (data production and analysis)

Charles B. Epstein[2], Noam Shoresh[2], Robbyn Issner[2], Shawn Gillespie[45], Dylan Rausch[45], Joseph Raymond[2], Shanna Hsu[2], Danielle Tenen[2], Oren Ram[2], Alon Goren[2], Russell Ryan[45], Mariateresa Fulciniti[46], David Hendrickson[2], Jonathan Scheiman[47], Birgit Knoechel[46,48], David Kelley[49], Samantha Beik[2], Yotam Drier[45], Carles Boix[35], Wouter Meuleman[5], Pouya Kheradpour[35], Nina Farrell[2], Meital Hatan[2], David Wine[2], Mia C. Uziel[2], Kristin G. Ardlie[2], Michael Mannstadt[45], Nikhil Munshi[46], Miguel Rivera[45], Alex Meissner[50], Manolis Kellis[2,35], John Rinn[33], Bradley E. Bernstein[42]

Cold Spring Harbor, Centre for Genomic Regulation (CRG), The Barcelona Institute of Science and Technology and Universitat Pompeu Fabra (data production and analysis)

Carrie A. Davis[4], Alexander Dobin[4], Alessandra Breschi[38], Sarah Djebali[38,51], Chris Zaleski[4], Dmitri D. Pervouchine[38,52], Anna Vlasova[38], Jorg Drenkow[53], Julien Lagarde[38], Rory Johnson[38,54], Barbara Uszczynska-Ratajczak[38,55], Alexandra Scavelli[4], Cassidy Danyko[4], Lei Hoon See[4], Roderic Guigó[38], Thomas R. Gingeras[4]

UConn Health, UCSD, Massachusetts Institute of Technology, and Institut de Recherches Cliniques de Montréal (IRCM) (data production and analysis)

Cassandra Bazile[31], Steven M. Blue[7], Eric L. Van Nostrand[7], Louis Philip Benoit Bouvrette[19,20,21], Daniel Dominguez[31], Peter Freese[8], Jia-Yu Chen[56], Neal A.L. Cody[19,20,21], Gabriel A. Pratt[7], Michael O. Duff[18], Sara Olson[18], Xiaofeng Wang[19,20,21], Keri Elkins[7], Balaji Sundararaman[7], Xintao Wei[18], Chelsea Anne Gelboin-Burkhart[7], Rui Xiao[56], Abigail Hochman[31], Lijun Zhan[18], Nicole J. Lambert[31], Hairi Li[56], Thai B. Nguyen[7], Tsultrim Palden[31], Ines Rabano[7], Shashank Sathe[7], Rebecca Stanton[7], Amanda Su[31], Ruth Wang[7], Brian A. Yee[7], Xiang-Dong Fu[56], Eric Lécuyer[19,20,21], Christopher B. Burge[31], Gene W. Yeo[7], Brenton R. Graveley[18]

Lécuyer[19,20,21], Christopher B. Burge[31], Gene W. Yeo[7], Brenton R. Graveley[18]

HudsonAlpha Institute for Biotechnology, California Institute of Technology, The Pennsylvania State University, National Human Genome Research Institute, University of Alabama in Huntsville, Duke University, University of California Irvine (data production and analysis)

Mark Mackiewicz[13], Florencia Pauli-Behn[13], E. Christopher Partridge[13], Daniel Savic[57], Brian S. Roberts[13], Kimberly M. Newberry[13], Laurel A. Brandsmeier[13], Sarah K. Meadows[13], Rosy Nguyen[13], Amy R. Nesmith[13], Dianna E. Moore[13], Christopher L. Messer[13], Megan McEown[13], Rachel C. Evans[13], J Scott Newberry[13], Collin White[13], Shawn Levy[13], Barbara Wold[14], Brian A. Williams[14], Diane E. Trout[14], Gilberto DeSalvo[14], Kenneth McCue[14], Peng He[14], Katherine Fisher-Aylor[14], Sean A. Upchurch[14], Henry Amrhein[14], Georgi K. Marinov[14], Jost Vielmetter[14], Anthony Kirilusha[14], Igor Antoshechkin[14], Ross C. Hardison[16], Cheryl A. Keller[16], Belinda M. Giardine[16], Maria Long[16], David M. Bodine[58], Elisabeth F. Heuston[58], Stacie M. Anderson[58], Eric M. Mendenhall[13,25], Surya B. Chhetri[13,25], Candice J. Coppola[13,25], Timothy E. Reddy[59,60], Anthony M. D'Ippolito[60], Christopher M. Vockley[2,60], Ali Mortazavi[15], Rabi Murad[15], Weihua Zeng[15], Camden Jansen[15], Ricardo N. Ramirez[15], Nicole El-Ali[15], Richard M. Myers[13]

University of California, San Diego, Salk Institute for Biological Studies, Lawrence Berkeley National Laboratory, UC San Diego, Howard Hughes Medical Institute (data production and analysis)

David U. Gorkin[9,10], Yupeng He[12], Iros Barozzi[17], Andre Wildberg[61], Jennifer A. Akiyama[17], Rosa G. Castanon[12], Sora Chee[10], Huaming Chen[12], Bo Ding[61], Yoko Fukuda-Yuzawa[17], Tyler H. Garvin[17], Manoj Hariharan[12], Anne Harrington[17], Hui Huang[10], Momoe Kato[17], Samantha Kuan[10], Ah Young Lee[10], Elizabeth Lee[17], Bin Li[10], Nan Li[61], Brandon J. Mannion[17], Joseph R. Nery[12], Vu Ngo[61], Tung Nguyen[61], Quan Pham[17], Catherine S. Novak[17], Ingrid Plajzer-Frick[17], Sebastian Preissl[10], Yin Shen[10,11], Mengchi Wang[61], Tao Wang[61], John W. Whitaker[61], Yanxiao Zhang[10], Kai Zhang[61], Yuan Zhao[10], Yun Zhu[61], Rizi Ai[61], Feng Yue[62,63], Veena Afzal[17], Diane E. Dickel[17], Valentina Snetkova[17], Wei Wang[61], Axel Visel[17,29,30], Len A. Pennacchio[17,29,40], Joseph R. Ecker[12,34], Bing Ren[9,10]

Stanford University, The University of Chicago, University of Southern California, University of Toronto, Yale University (data production and analysis)

Jessika Adrian[3], Trupti Kawli[3], Nicholas J. Addleman[3], Alan P. Boyle[64,65], Lulu Cao[3], Hassan

Chaib[3], Songjie Chen[3], Catharine Eastman[66], Andrew Emili[67], Peng Gong[26], Fabian Grubert[3], Yu Guo[39], Hongbo Guo[67], Nastaran Heidari[3,68], Cory Holgren[27], Nader Jameel[27], Lixia Jiang[3], Madhura Kadaba[27], Maya Kasowski[3], Mary Kasparian[27], Yining Li[3], Jin Lian[26], Yiing Lin[69], Shin Lin[3], Lijia Ma[27], Matthew G. Milton[27], Tejaswini Mishra[3], Jennifer Moran[27], Anil M. Narasimha[3], Xinghua Pan[26,70,71], Doug H. Phanstiel[72,73], Ernest Radovani[67], Lucia Ramirez[3], Rozita Razavi[74], Suhn K. Rhie[39], Denis N. Salins[3], Frank W. Schmitges[74], Quan Shen[26,75], Minyi Shi[3], Teri Slifer[3], Damek V. Spacek[3], Rohith Srivas[3], Dave Steffan[27], Matt Szynkarek[27], Dave Toffey[27], Alec Victorsen[27], Nathaniel K. Watson[3], Heather N. Witt[39], Xinqiong Yang[3], Jie Zhai[3], Jialing Zhang[26], Guoqing Zhong[67], Sherman M. Weissman[26], Jack F. Greenblatt[67,74], Timothy R. Hughes[67,76], Peggy J. Farnham[39], Kevin P. White[28], Michael P. Snyder[3,41]

Altius Institute for Biomedical Sciences, University of Washington, Fred Hutchinson Cancer Research Center, University of Massachusetts Medical School, Howard Hughes Medical Institute (data production and analysis)

John A. Stamatoyannopoulos[5,6,37], Rajinder Kaul[5,6], Jessica Halow[5], Richard Sandstrom[5], Michael Buckley[5], Jeff Vierstra[5], Wouter Meuleman[5], Eric Haugen[5], Shane Neph[5], Andrew Nishida[5], Alex Reynolds[5], Eric Rynes[5], Audra Johnson[5], Jemma Nelson[5], Alister P. W. Funnell[5], Vivek Nandakumar[5], Kyle Siebenthall[5], Hao Wang[5], Yongqi Yan[5], Reyes Acosta[5], Kristen Lee[5], Ericka Otterman[5], Benjamin Van Biber[5], Mineo Iwata[5], Tanya Kutyavin[5], Sandra Stehling-Sun[5], Robert E. Welikson[5], Andres Castillo[5], Grigorios Georgolopoulos[5], Sean Ibarrientos[5], Fidencio Jun Neri[5], Anthony Shafer[5], Shinny Vong[5], Daniel Bates[5], Morgan Diegel[5], Douglass Dunn[5], John Lazar[5], Daniel R. Chee[5], George Stamatoyannopoulos[6,77], Patrick Navas[5], M. A. Bender[78], Mark T. Groudine[78], Rachel Byron[78], Ye Zhan[32], Hakan Ozadam[32], Bryan R. Lajoie[32], Job Dekker[32]

Data Coordination Center at Stanford (data coordination center)

J. Michael Cherry[3], Benjamin C. Hitz[3], Cricket A. Sloan[3], Ulugbek K. Baymuradov[3], Esther T. Chan[3], Timothy R. Dreszer[3], Idan Gabdank[3], Jason A. Hilton[3], Aditi K. Narayanan[3], Kathrina C. Onate[3], J. Seth Strattan[3], Forrest Y. Tanaka[3]

University of Massachusetts Medical School, Yale University, Stanford University, Centre for Genomic Regulation (CRG), The Barcelona Institute of Science and Technology and Universitat Pompeu Fabra, University of Washington, Dana-Farber Cancer Institute, Harvard School of Public Health, Massachusetts Institute of Technology (data analysis

center)

Jill E. Moore[1], Michael J. Purcaro[1], Henry E. Pratt[1], Gregory R. Andrews[1], Tyler Borrman[1], Jason A. Brooks[1], Hao Chen[1,44], Shaimae I. Elhajjajy[1], Kaili Fan[1], Kevin Fortier[1], Mingshi Gao[1], Jack Huey[1], Eugenio Mattei[1], Nishigandha N. Phalke[1], Thomas M. Reimonn[1], Shuo Shan[1], Junko Tsuji[1], Arjan G. van der Velde[1,44], Taylor Young[1], Xiao-Ou Zhang[1], Tianxiong Yu[43], Peng Zhang[43], Lingling Cai[43], Xiangrui Li[43], Ying Li[43], Zhijie Wei[43], Shuyu Hou[43], Aiping Lu[43], Yan Liu[43], Yu Fu[1,79], Shaliu Fu[43], Qin Wang[43], Joel Rozowsky[24], Jing Zhang[24], Koon-Kiu Yan[24], Mengting Gu[24], Donghoon Lee[24], Shaoke Lou[24], Anurag Sethi[24], Sushant Kumar[24], Timur Galeev[24], Arif Harmanci[24], Gamze Gursoy[24], Jason Liu[24], Jinrui Xu[24], Fabio C.P. Navarro[24], Shantao Li[24], Nathan Boley[3], Daniel Sunwook Kim[3], Jin Wook Lee[3], Chuan Sheng Foo[80], Oana Ursu[3], Sarah Djebali[38,51], Dmitri D. Pervouchine[38,52], Anna Vlasova[38], Beatrice Borsari[38], Maxwell W. Libbrecht[81], Galip Gürkan Yardımcı[37], Peng Jiang[82,83], Clifford Meyer[82,83], Chongzhi Zang[82,83,84], Qian Qin[47,85], Mingxiang Teng[86], Jose Davila-Velderrain[35], Yue Li[35], Zhizhuo Zhang[35], Dianbo Liu[35], Carles Boix[35], Yongjin Park[35], Yaping Liu[35], Lei Hou[35], Manolis Kellis[2,35], X. Shirley Liu[82,83], William S. Noble[37], Roderic Guigó[38], Anshul Kundaje[3], Mark B. Gerstein[24], Zhiping Weng[1,43,44]

Massachusetts Institute of Technology (computational algorithm development)

Amira A. Barkal[87], Budhaditya Banerjee[88], Matthew D. Edwards[35], David K. Gifford[35], Yuchun Guo[35], Tatsunori B. Hashimoto[35], Tommi Jaakkola[35], Charles W. O'Donnell[35], Nisha Rajagopal[88], Richard I. Sherwood[88], Sharanya Srinivasan[88], Tahin Syed[35], Haoyang Zeng[35]

University of Wisconsin–Madison, University of Nebraska-Lincoln, The Ohio State University, University of Wisconsin School of Medicine and Public Health (computational algorithm development)

Ye Zheng[89], Peng Liu[90], Sunyoung Shin[91], Rene Welch[90], Jurijs Nazarovs[89], Qi Zhang[92], Dongjun Chung[93], Emery H. Bresnick[94], Colin N. Dewey[90], Sunduz Keles[89,90]

Icahn School of Medicine at Mount Sinai, Brigham and Women's Hospital and Harvard Medical School, Memorial Sloan Kettering Cancer Center (computational algorithm development)

James E. Hayes[36], Gosia Trynka[95], Alvaro Gonzalez[96], Harm-Jan Westra[88], Manu Setty[96], Maria Gutierrez-Arcelus[88], Yuheng Lu[96], Alexander R. Perez[96], Yuri Pritykin[96], Mark Carty[96], Christina S. Leslie[96], Soumya Raychaudhuri[88], Robert J. Klein[36]

Stanford (computational algorithm development)
Anand Bhaskar[3], Yang I. Li[3], Graham McVicker[12], Eilon Sharon[3], Anil Raj[3], Jonathan K. Pritchard[3]

University of California, Los Angeles (computational algorithm development)
Yun-Hua E. Hsiao[97], Giovanni Quinones-Valdez[97], Yi-Wen Yang[97], Xinshu Xiao[97]

Johns Hopkins University (data analysis)
Michael A. Beer[98,99]

Pennsylvania State University/Northwestern University (data production and analysis)
Yanli Wang[63], Hongbo Yang[63], Tingting Liu[63], Lijun Zhang[63], Jie Xu[63], Bo Zhang[63], Feng Yue[62,63]

European Bioinformatics Institute (EMBL-EBI), Centre for Genomic Regulation (CRG), The Barcelona Institute of Science and Technology and Universitat Pompeu Fabra, ELIXIR Hub, King's College London, Guy's Hospital, Massachusetts Institute of Technology, Spanish National Cancer Research Centre (CNIO), University of Bern, University of California, Santa Cruz, University of Lausanne, Wellcome Sanger Institute, Yale University (gene annotation)
Bronwen Aken[100], Joel Armstrong[101], Matthew Astley[95], If H.A. Barnes[100], Daniel Barrell[100], Gemma Barson[95], Ruth Bennett[100], Andrew Berry[100], Alexandra Bignell[100], Jacqueline Chrast[102], Declan Clarke[24], Claire Davidson[100], Alden Deran[101], Gloria Despacio-Reyes[95], Mark Diekhans[101], Sarah Donaldson[100], Iakes Ezkurdia[103], Anne-Maud Ferreira[102], Stephen Fitzgerald[95], Carlos Garcia Giron[100], Jose M. Gonzalez[100], Michael Gray[95], Ed Griffiths[95], Matthew Hardy[100], Toby Hunt[100], Rory Johnson[38,54], Irwin Jungreis[2,35], Michael Kay[100], Julien Lagarde[38], Jane Loveland[100], Deepa Manthravadi[95], Osagie Izuogu[100], Fergal J. Martin[100], Steve Miller[95], Jonathan M. Mudge[100], Eva Maria Novoa[35], Baikang Pei[24], Dmitri D. Pervouchine[38,52], Jose M. Rodrigez[103], Christoph Schlaffner[95], Cristina Sisu[24,105], Marie-Marthe Suner[100], Michael L. Tress[104], Barbara Uszczynska-Ratajczak[38,55], Jesus Vazquez[103], Hendrik Weisser[95], Maxim Wolf[35], James Wright[95], Tim J. Hubbard[106], Jennifer L. Harrow[107], Alfonso Valencia[104], Federico Abascal[95], Manolis Kellis[2,35], Paul Flicek[100], Benedict Paten[101], Jyoti S. Choudhary[108], Mark B. Gerstein[24], Alexandre Reymond[102], Roderic Guigó[38], Adam Frankish[100]

Florida State University and University of Georgia (data production and analysis)
Juan Carlos Rivera-Mulia[22,23], Takayo Sasaki[22], Vishnu Dileep[22], Jared Zimmerman[22], Michael J. Kulik[109], Stephen Dalton[110], David M. Gilbert[22]

European Bioinformatics Institute (EMBL-EBI) (genome annotation)
Emily H. Perry[100], Daniel R. Zerbino[100], Paul Flicek[100]

The Broad Institute of Harvard and MIT, Gift of Life Donor Program, American Society for Radiation Oncology, National Cancer Institute (NCI), Leidos Biomedical, Inc., National Disease Research Interchange (NDRI), National Human Genome Research Institute (NHGRI) (tissue sample preparation)
Kristin G. Ardlie[2], Richard D. Hasz[111], Judith C. Keen[112], Helen M. Moore[113], Anna Smith[114], Jeffrey A. Thomas[115], Simona Volpi[116]

National Human Genome Research Institute (Project Management)
Xiao-Qiao Zhou[116], Hannah Naughton[116], Julie Coursen[116], Samuel H. Moore[116], Preetha Nandi[116], Omar Al Jammal[116], Yekaterina Vaydylevich[116], Peter Good[117], Jeffery A. Schloss[116], Briana Nuñez[116], Michael Pagan[116], Eileen Cahill[116], Daniel A. Gilchrist[116], Michael J. Pazin[116], Elise A. Feingold[116]
(*The role of the NHGRI Project Management Group in the preparation of this paper was limited to coordination and scientific management of the ENCODE consortium.*)

Affiliations
1) University of Massachusetts Medical School, Program in Bioinformatics and Integrative Biology, Worcester, MA, USA.
2) The Broad Institute of Harvard and MIT, Cambridge, MA, USA.
3) Department of Genetics, School of Medicine, Stanford University, Palo Alto, CA, USA.
4) Cold Spring Harbor Laboratory, Functional Genomics, Cold Spring Harbor, NY, USA.
5) Altius Institute for Biomedical Sciences, Seattle, WA, USA.
6) Department of Medicine, University of Washington School of Medicine, Seattle, WA, USA.
7) Department of Cellular and Molecular Medicine, Institute for Genomic Medicine, Stem Cell Program, Sanford Consortium for Regenerative Medicine, University of California, San Diego, La Jolla, CA, USA.
8) Program in Computational and Systems Biology, Massachusetts Institute of Technology, Cambridge, MA, USA.
9) Center for Epigenomics, Department of Cellular and Molecular Medicine, University of California, San Diego, La Jolla, CA, USA
10) Ludwig Institute for Cancer Research, University of California, San Diego, La Jolla, CA, USA.
11) Institute for Human Genetics, Department of Neurology, University of California, San Francisco, San Francisco, CA, USA.
12) Genomics Analysis Laboratory, The Salk Institute for Biological Studies, La Jolla, CA, USA.
13) HudsonAlpha Institute for Biotechnology, Huntsville, AL, USA.
14) Division of Biology and Biological Engineering, California Institute of Technology, Pasadena, CA, USA.
15) Department of Developmental and Cell Biology, University of California Irvine, Irvine, CA, USA.
16) Department of Biochemistry and Molecular Biology, The Pennsylvania State University, University Park, PA, USA.
17) Environmental Genomics and Systems Biology Division, Lawrence Berkeley National Laboratory, Berkeley, CA, USA.
18) Department of Genetics and Genome Sciences, Institute for Systems Genomics, UConn Health, Farmington, CT, USA.
19) Département de Biochimie et Médecine Moléculaire, Université de Montréal, Montréal, Quebec, Canada.
20) Division of Experimental Medicine, McGill University, Montreal, Quebec, Canada.

21) Institut de Recherches Cliniques de Montréal (IRCM), Montréal, Quebec, Canada.
22) Department of Biological Science, Florida State University, Tallahassee, FL, USA.
23) Department of Biochemistry, Molecular Biology and Biophysics, University of Minnesota Medical School, Minneapolis, MN, USA.
24) Yale University, New Haven, CT, USA.
25) Biological Sciences, University of Alabama in Huntsville, Huntsville, AL, USA.
26) Department of Genetics, School of Medicine, Yale University, New Haven, CT, USA.
27) Department of Human Genetics, Institute for Genomics and Systems Biology, The University of Chicago, Chicago, IL, USA.
28) Tempus Labs, Chicago, IL,USA.
29) US Department of Energy Joint Genome Institute, Lawrence Berkeley National Laboratory, Berkeley, CA, USA.
30) School of Natural Sciences, University of California, Merced, Merced, CA, USA.
31) Department of Biology, Massachusetts Institute of Technology, Cambridge, MA, USA.
32) HHMI and Program in Systems Biology, University of Massachusetts Medical School, Worcester, MA, USA.
33) University of Colorado Boulder, Boulder, CO, USA.
34) Howard Hughes Medical Institute, The Salk Institute for Biological Studies, La Jolla, CA, USA.
35) Computer Science and Artificial Intelligence Laboratory, Massachusetts Institute of Technology, Cambridge, MA, USA.
36) Department of Genetics and Genomic Sciences, Icahn School of Medicine at Mount Sinai, New York, NY, USA.
37) Department of Genome Sciences, University of Washington School of Medicine, Seattle, WA, USA.
38) Bioinformatics and Genomics Program, Centre for Genomic Regulation (CRG) , The Barcelona Institute of Science and Technology and Universitat Pompeu Fabra, Barcelona, Spain.
39) Department of Biochemistry and Molecular Medicine, Norris Comprehensive Cancer Center, Keck School of Medicine, University of Southern California, Los Angeles, CA, USA.
40) Comparative Biochemistry Program, University of California, Berkeley, CA, USA.
41) Cardiovascular Institute, Stanford School of Medicine, Stanford, CA, USA.
42) Broad Institute and Department of Pathology, Massachusetts General Hospital and Harvard Medical School, Boston, MA, USA.

43) Department of Thoracic Surgery, Clinical Translational Research Center, Shanghai Pulmonary Hospital, The School of Life Sciences and Technology, Tongji University, Shanghai, China.
44) Bioinformatics Program, Boston University, Boston, MA, USA.
45) MGH, Boston, MA, USA.
46) Dana-Farber Cancer Institute, Boston, MA, USA.
47) Harvard Medical School, Boston, MA, USA.
48) Boston Children's Hospital, Boston, MA, USA.
49) Harvard University, Cambridge, MA, USA.
50) Max Planck Institute for Molecular Genetics, Department of Genome Regulation, Berlin, Germany.
51) IRSD, Université de Toulouse, INSERM, INRA, ENVT, UPS, U1220, CHU Purpan, CS60039, Toulouse, France.
52) Skolkovo Institute for Science and Technology, Moscow, Russia.
53) Cold Spring Harbor Laboratory, Woodbury, NY, USA.
54) Department of Clinical Research, University of Bern, Bern, Switzerland.
55) International Institute of Molecular and Cell Biology, Warsaw, Poland.
56) Department of Cellular and Molecular Medicine, Institute of Genomic Medicine, University of California at San Diego, San Diego, CA, USA.
57) Department of Pharmaceutical Sciences, St. Jude Children's Research Hospital, Memphis, TN, USA.
58) National Human Genome Research Institute, National Institutes of Health, Bethesda, MD, USA.
59) Department of Biostatistics and Bioinformatics, Duke University, Durham, NC, USA.
60) Center for Genomic and Computational Biology, Duke University, Durham, NC, USA.
61) Department of Chemistry and Biochemistry, Department of Cellular and Molecular Medicine, UC San Diego, La Jolla, CA, USA.
62) Department of Biochemistry and Molecular Genetics, Northwestern University Feinberg School of Medicine, Chicago, IL, USA.
63) Penn State Health Milton S. Hershey Medical Center, Hershey, PA, USA.
64) Department of Computational Medicine and Bioinformatics, University of Michigan, Ann Arbor, MI, USA.
65) Department of Human Genetics, University of Michigan, Ann Arbor, MI, USA.

66) Department of Molecular and Cellular Physiology, School of Medicine, Stanford University, Palo Alto, CA, USA.
67) Terrence Donnelly Centre for Cellular and Biomolecular Research, University of Toronto, Toronto, Ontario, Canada.
68) Department of Radiation Oncology, School of Medicine, Stanford University, Palo Alto, CA, USA.
69) Division of General Surgery, Section of Transplant Surgery, School of Medicine, Washington University, St. Louis, MO, USA.
70) Department of Biochemistry and Molecular Biology, School of Basic Medical Sciences, Southern Medical University, Guangzhou, China.
71) Guangdong Provincial Key Laboratory of Single Cell Technology and Application, Guangzhou, China.
72) Department of Cell Biology & Physiology, University of North Carolina at Chapel Hill, Chapel Hill, NC, USA.
73) Thurston Arthritis Research Center, University of North Carolina at Chapel Hill, Chapel Hill, NC, USA.
74) Department of Molecular Genetics, University of Toronto, Toronto, Ontario, Canada.
75) School of Medicine, Jiangsu University, Zhenjiang, China.
76) Department of Molecular Genetics, Donnelly Centre, University of Toronto, Toronto, Ontario, Canada.
77) Division of Medical Genetics, University of Washington School of Medicine, Seattle, WA, USA.
78) Fred Hutchinson Cancer Research Center, Seattle, WA, USA.
79) University of Massachusetts Amherst, Amherst, MA, USA.
80) Institute for Infocomm Research, Singapore, Singapore.
81) Simon Fraser University, Burnaby, British Columbia, Canada.
82) Center for Functional Cancer Epigenetics, Dana-Farber Cancer Institute, Boston, MA, USA.
83) Department of Data Sciences, Dana-Farber Cancer Institute and Harvard T.H. Chan School of Public Health, Boston, MA, USA.
84) Center for Public Health Genomics, University of Virginia, Charlottesville, VA, USA.
85) Molecular Pathology Unit & Cancer Center, Massachusetts General Hospital and Harvard Medical School, Boston, MA, USA.
86) Department of Biostatistics and Bioinformatics, Moffitt Cancer Center, Tampa, FL, USA.
87) Institute for Stem Cell Biology and Regenerative Medicine, Stanford University, Stanford, CA, USA.

88) Department of Medicine, Brigham and Women's Hospital and Harvard Medical School, Boston, MA, USA.
89) Department of Statistics, Medical Sciences Center, University of Wisconsin - Madison, Madison, WI, USA.
90) Department of Biostatistics and Medical Informatics, University of Wisconsin - Madison, Madison, WI, USA.
91) Department of Mathematical Sciences, University of Texas at Dallas, Richardson, TX, USA.
92) Department of Statistics, University of Nebraska-Lincoln, Lincoln, NE, USA.
93) Department of Biomedical Informatics, The Ohio State University, Columbus, OH, USA.
94) Department of Cell and Regenerative Biology, UW-Madison Blood Research Program, Carbone Cancer Center, University of Wisconsin School of Medicine and Public Health, University of Wisconsin, Madison, WI, USA.
95) Wellcome Sanger Institute, Cambridge, UK.
96) Program in Computational Biology, Memorial Sloan Kettering Cancer Center, New York, NY, USA.
97) Department of Integrative Biology and Physiology, University of California Los Angeles, Los Angeles, CA, USA.
98) McKusick-Nathans Institute of Genetic Medicine, Johns Hopkins University, Baltimore, MD, USA.
99) Department of Biomedical Engineering, Johns Hopkins University, Baltimore, MD, USA.
100) European Molecular Biology Laboratory, European Bioinformatics Institute, Wellcome Genome Campus, Cambridge, United Kingdom.
101) University of California, Santa Cruz, Santa Cruz, CA, USA.
102) Center for Integrative Genomics, University of Lausanne, Lausanne, Switzerland.
103) Centro Nacional de Investigaciones Cardiovasculares (CNIC) and CIBER de Enfermedades Cardiovasculares (CIBERCV) , Madrid, Spain.
104) Spanish National Cancer Research Centre (CNIO), Madrid, Spain.
105) Brunel University London, London, UK.
106) King's College London, Guy's Hospital, London, UK.
107) ELIXIR Hub, Wellcome Genome Campus, Cambridge, UK.
108) Institute of Cancer Research, Chester Betty Labs, London, UK.
109) Center for Vaccines and Immunology, University of Georgia, Athens, GA, USA.

110) Center for Molecular Medicine and Department of Biochemistry and Molecular Biology, University of Georgia, Athens, GA, USA.
111) Gift of Life Donor Program, Philadelphia, PA, USA.
112) American Society for Radiation Oncology, Arlington, VA, USA.
113) National Cancer Institute, National Institutes of Health, Bethesda, MD, USA.
114) Leidos Biomedical, Inc., Frederick, MD, USA.
115) National Disease Research Interchange (NDRI), Philadelphia, PA, USA.
116) National Human Genome Research Institute, National Institutes of Health, Bethesda, MD, USA.
117) 4407 Puller Drive, Kensington, MD, USA.

Current Affiliations:

Roy Johnson: Department of Medical Oncology, Inselspital, University Hospital and University of Bern, Switzerland. Department of Biomedical Research, University of Bern, Switzerland.

Jing Zhang: School of Information and Computer Sciences, University of California, Irvine, CA, USA.

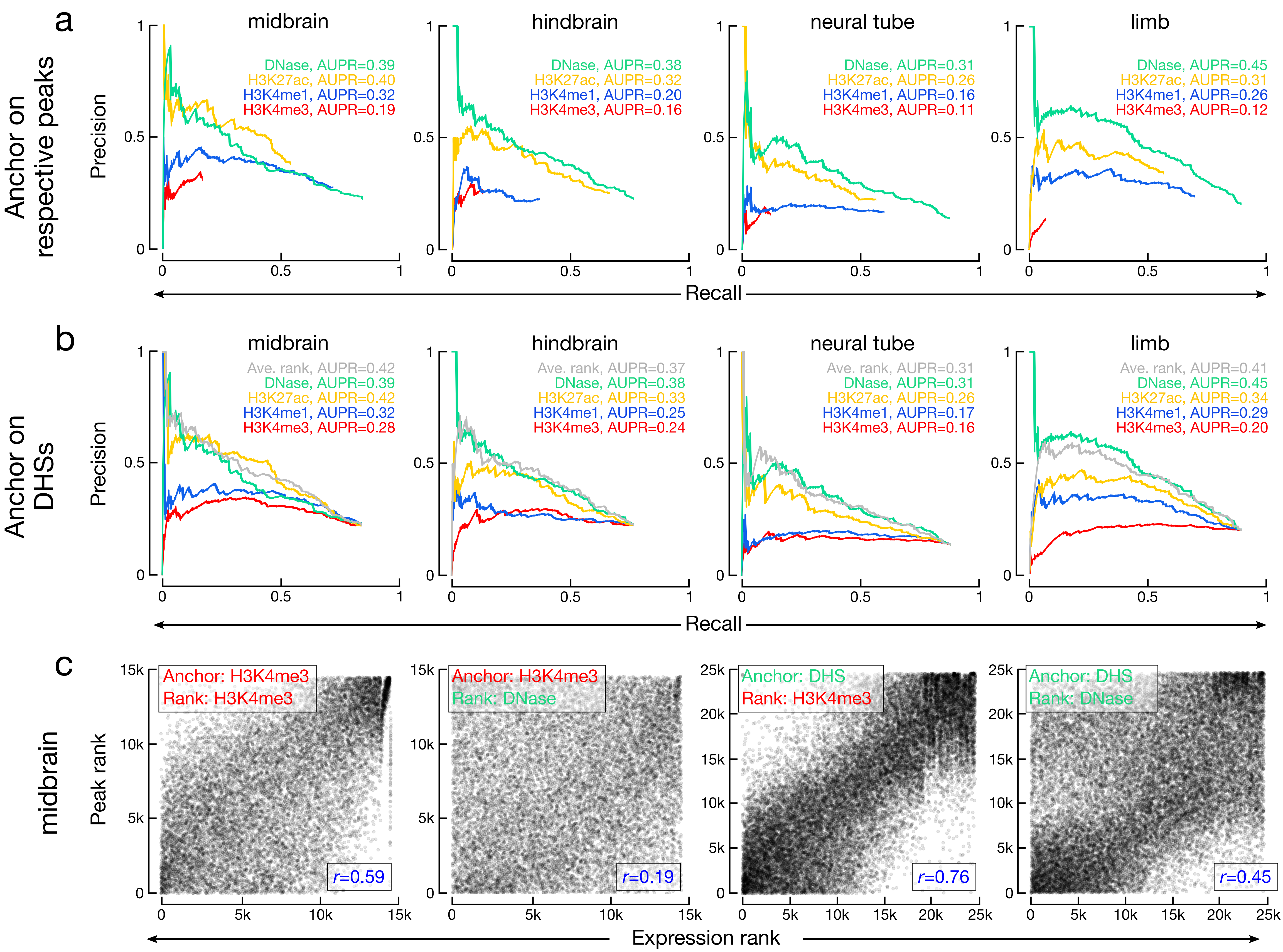

Supplementary Figure 1 | Testing methods for predicting VISTA enhancers and gene expression. a, PR curves for predicting e11.5 midbrain, hindbrain, neural tube, and limb enhancers (1,994 regions evaluated in each plot). Colors indicate the epigenetic features whose peaks were used to anchor the enhancer predictions and whose signals were used to rank the predictions. **b,** Same as in **a,** except that all enhancer predictions were anchored on DHSs in the respective tissue. Predictions were still ranked by the signal levels of the respective epigenetic features indicated by colors as in **a**. Gray lines indicate the performance of the average rank of DNase and H3K27ac signals. **c,** Scatter plots depict correlation between predicted and measured transcript expression (103,639 total transcripts) in e11.5 midbrain with the predictions being H3K4me3 peaks ranked by their H3K4me3 signals (Pearson's correlation r = 0.59), H3K4me3 peaks ranked by their DNase signal (r = 0.19), DHSs ranked by their H3K4me3 signals (r = 0.76), and DHSs ranked by their DNase signals (r = 0.45).

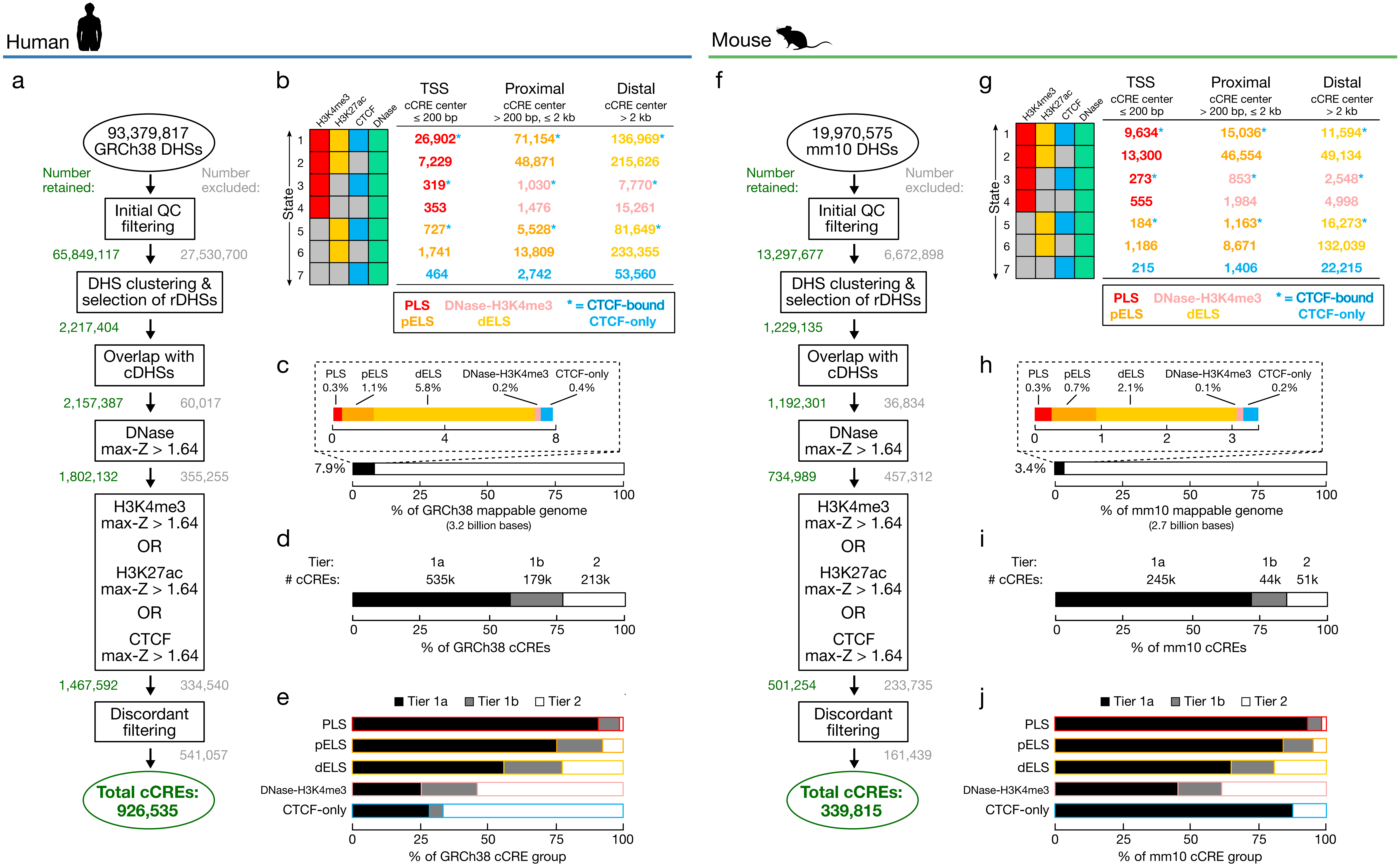

Supplementary Figure 2 | Details of building the Registry of cCREs. a, Selection of human cCREs. We began by clustering DNase peaks that passed quality-control thresholds (false discovery rate < 0.1%, DNase signal > 10th percentile) and selected a representative DHS (rDHS) for each cluster. We discarded the rDHSs that did not overlap consensus DHSs (see Supplementary Methods). Then, we selected rDHSs with high DNase max-Z scores and additionally high max-Z score for at least one other assay (H3K4me3, H3K27ac, or CTCF) and further filtered out those rDHSs for which high DNase and ChIP Z-scores were not from the same biosample with experimental data (see concordancy test in Supplementary Methods). In total, this resulted in 926,535 cCREs in human. **b,** Classification of cCREs into five cell type-agnostic groups (PLS, pELS, dELS, DNase-H3K4me3, or CTCF-only) based on their states of high or low H3K4me3, H3K27ac or CTCF max-Z scores and genomic context (TSS-overlapping, TSS-proximal, or TSS-distal). **c,** Percentages of the 3.2 billion mappable nucleotides of the GRCh38 genome occupied by the five groups of cCREs. **d,** Breakdown of cCREs by tiers (defined in Box 2 and Supplementary Methods). **e,** Breakdown of the five groups of cCREs by tiers. **f-j,** Selection and classification of mouse cCREs as for human in **a-e**.

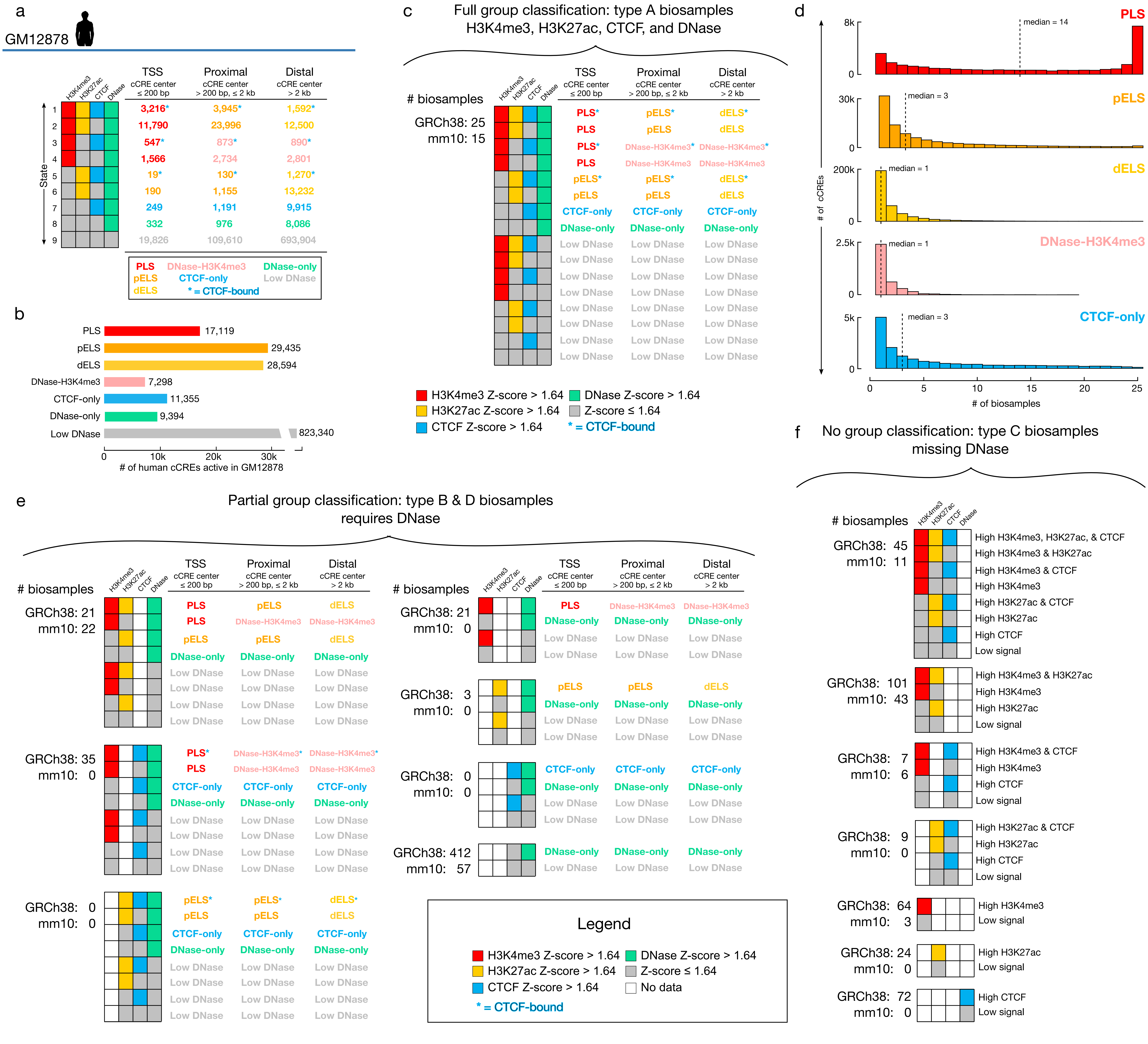

Supplementary Figure 3 | Classification of cCREs in a particular biosample. a, Classification of seven groups of cCREs (PLS, pELS, dELS, DNase-H3K4me3, CTCF-only, DNase-only, or Low DNase) in GM12878 by their states of high or low H3K4me3, H3K27ac, or CTCF Z-scores in GM12878. **b,** Number of each group of GM12878 cCREs. **c,** Full classification scheme for biosamples covered by all four core assays (H3K4me3, H3K27ac, CTCF, and DNase). **d,** Distribution of each group of cCREs across the 25 human biosamples fully covered by the four assays. **e,** Partial classification scheme for biosamples with DNase data that lack one or more types of ChIP-seq data. **f,** In biosamples lacking DNase data, cCREs are not assigned to groups but rather classified as whether or not they have high ChIP-seq signals.

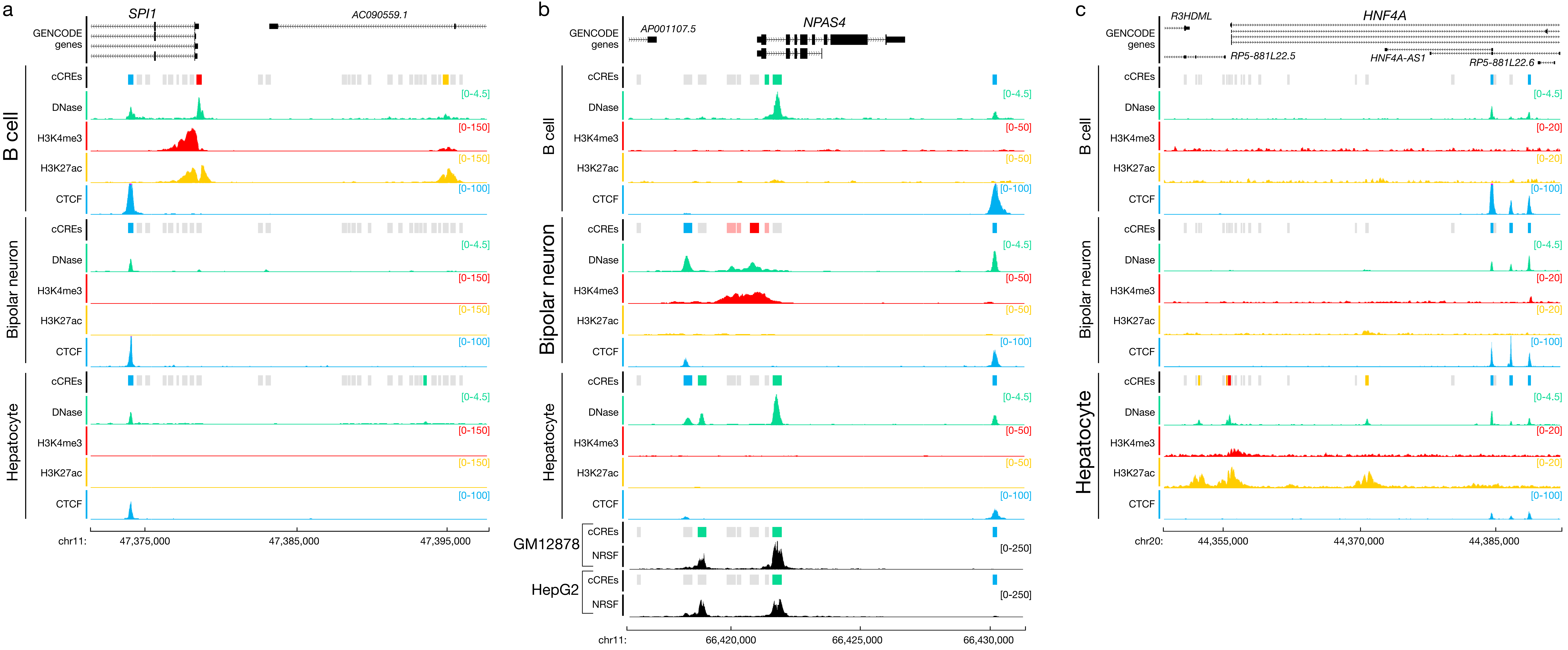

Supplementary Figure 4 | UCSC Genome Browser views of cCREs and the underlying DNase and ChIP data. Three loci are shown—**a,** *SPI1* **b,** *NPAS4*, and **c,** *HNF4A*—which are active in B cells, bipolar spindle neurons, and hepatocytes, respectively, signified by a larger font for the corresponding biosample names. The cCREs classified in each biosample (PLS in red, pELS in orange, dELS in yellow, DNase-H3K4me3 in pink, CTCF-only in blue, DNase-only in green, and low DNase in gray) are shown above the signal profiles for the four core assays (DNase-seq in green, H3K4me3 ChIP-seq in red, H3K27ac ChIP-seq in yellow, and CTCF ChIP-seq in blue) in each of the three biosamples. In bipolar neurons, additional ChIP-seq data for NRSF are shown in GM12878 and HepG2 cells (black), suggesting NRSF binding at the DNase-only cCREs in B cells and hepatocytes.

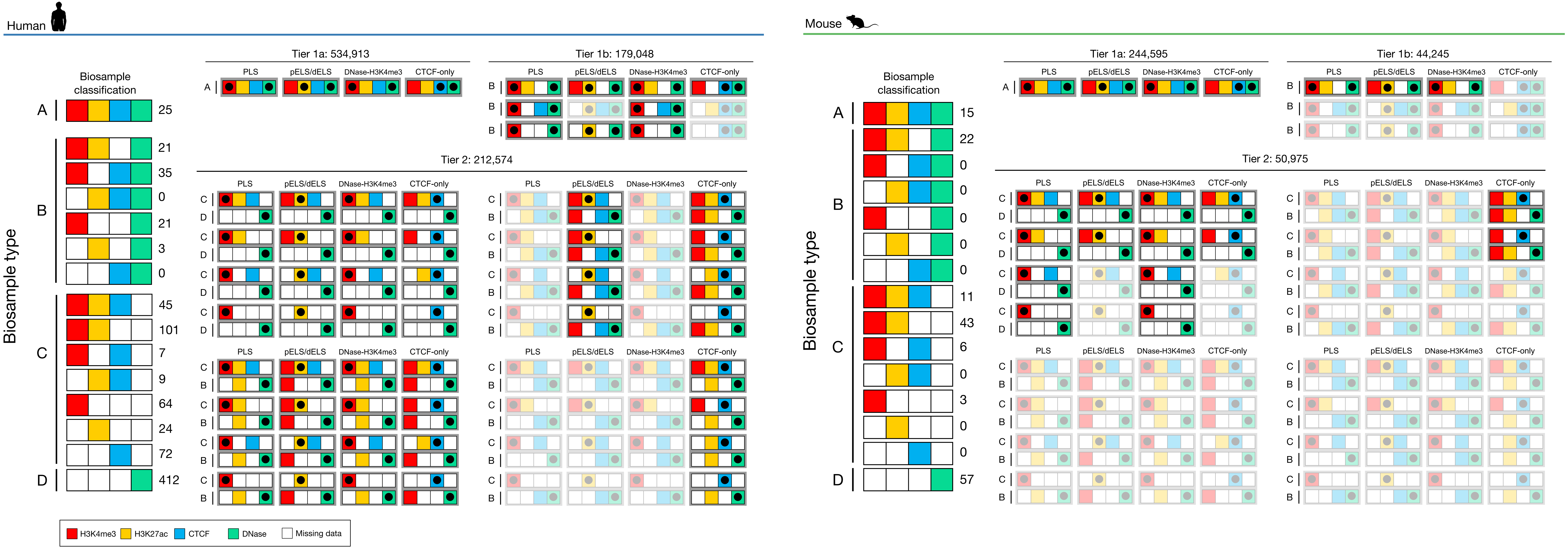

Supplementary Figure 5 | Classification of cCREs into Tiers based on biosample support. Two large panels are shown, with the human left and mouse right. For each species, biosamples are classified based on whether they have data for the four core assays (colored squares indicate available data and white squares indicate missing data): all four assays (type A), DNase and one or two ChIP assays (type B), only ChIP data (type C) or only DNase data (type D). Numbers to the right of the four assay squares designate the number of biosamples with each combination of assay coverage by existing ENCODE and Roadmap data. All cCREs are classified into tiers with Tier 1a and 1b cCREs (defined using type A and B biosamples, respectively) supported by high DNase and high ChIP signals in the same biosample, while Tier 2 cCREs are supported by high signals in different biosamples as a result of incomplete assay coverage for type B, C, and D biosamples. A black dot in an assay square indicates a high signal for that assay and the lack of a black dot indicates a low signal. All possible combinations of assay coverage are shown, with those combinations not represented by current ENCODE data grayed out.

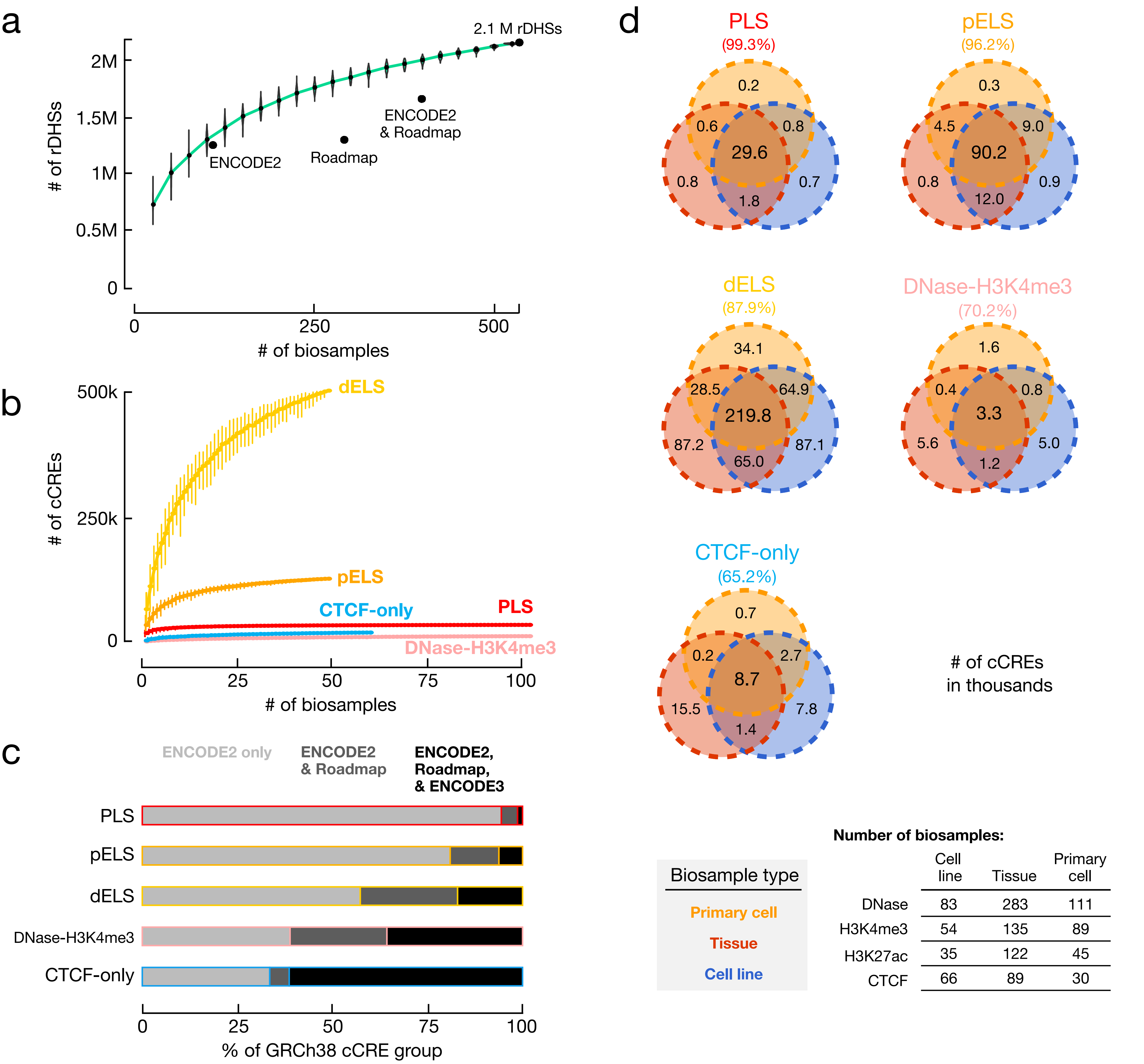

	Cell line	Tissue	Primary cell
DNase	83	283	111
H3K4me3	54	135	89
H3K27ac	35	122	45
CTCF	66	89	30

Supplementary Figure 6 | Impact of ENCODE Phase III data on the Registry. a, To estimate the coverage of the current Registry of human cCREs, we generated rDHSs by varying the number of biosamples, randomly selecting one hundred datasets each time. Violin plots represent all one hundred randomizations. The black dots indicate the number of rDHSs resulting from using only ENCODE phase II data, only Roadmap Epigenomics data, or only ENCODE phase II data and Roadmap Epigenomics data. Note that the Roadmap data point falls below the curve because the Roadmap Epigenomics project assayed tissues from multiple donors, resulting in a less diverse panel of biosamples than the ENCODE Project. **b,** Saturation curves for Tier 1 cCREs stratified by class. We randomly selected biosamples one hundred times and counted the number of unique cCREs. Because there are more biosamples with DNase and H3K4me3 data, the saturation curves continue farther along the x-axis for cCREs-PLS and DNase-H3K4me3 than cCREs-ELS or CTCF-only. Violin plots represent all one hundred randomizations. **c,** Number of cCREs stratified by group that are identified using only ENCODE Phase II data (light gray), using ENCODE Phase II and Roadmap data (dark gray), and using ENCODE Phase II, Roadmap, and ENCODE Phase III datasets (black). **d,** Number of cCREs in thousands identified using only data from primary cells (orange), tissues (red), or cell lines (dark blue) stratified by cCRE group. Percentage indicates the total number of cCREs of the group in the Venn diagram. The table indicates the number of experiments by assay for each biosample type.

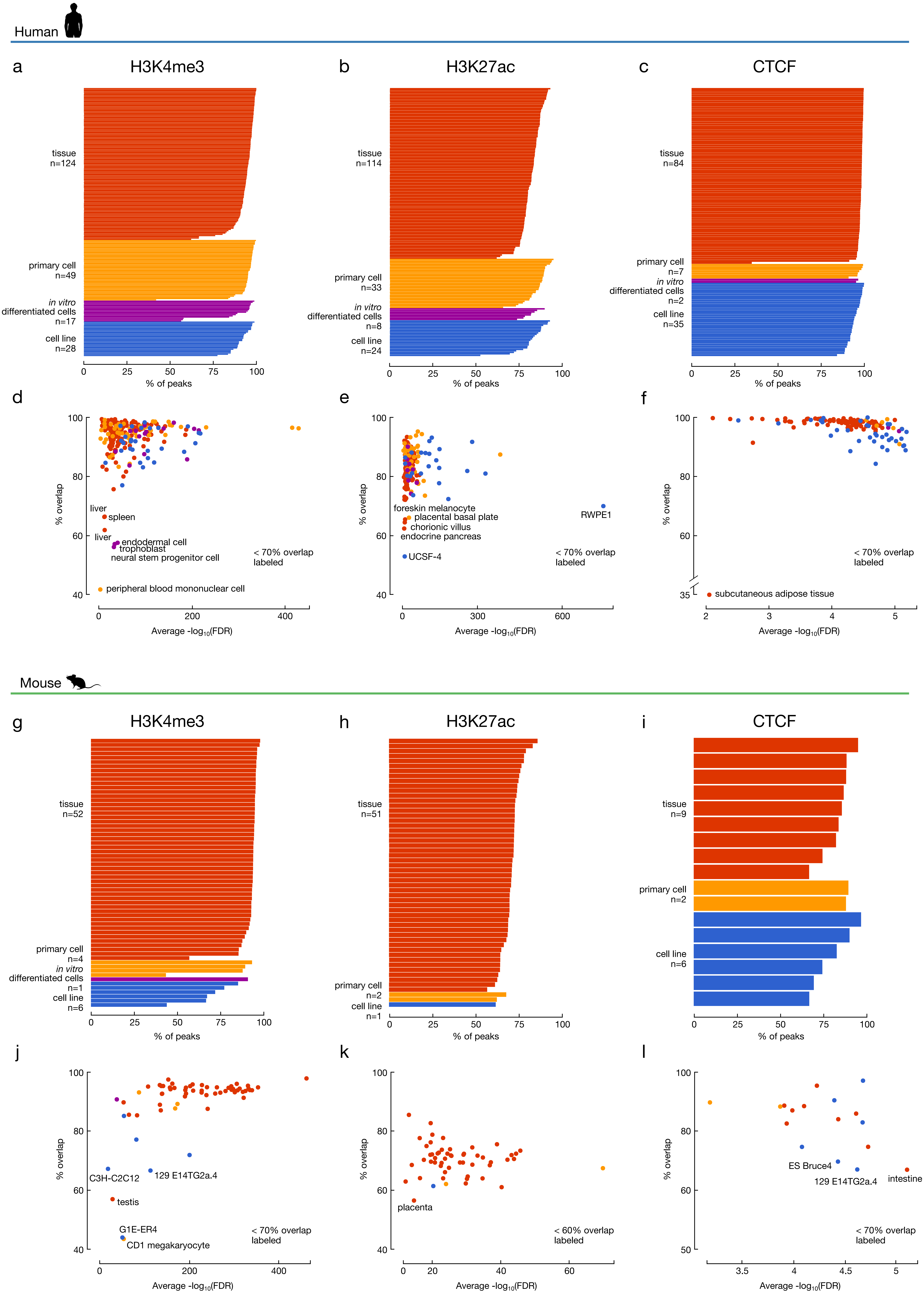

Supplementary Figure 7 | Coverage of the current Registry of cCREs. a-c, Percentages of human H3K4me3 (N=218), H3K27ac (N=180) and CTCF (N=129) ChIP-seq peaks from biosamples without DNase data that are covered by human cCREs. **d-f,** Scatter plots of average -log(FDR) of peaks vs percent overlap for H3K4me3 (**d**), H3K27ac (**e**) and CTCF (**f**) ChIP-seq peaks. Cell types with peaks that had a lower average -log10(FDR) tended to have a lower percentage of peaks covered by cCREs. **g-i,** Percentages of mouse H3K4me3 (N=64), H3K27ac (N=55), and CTCF (N=18) ChIP-seq peaks from biosamples without DNase data that are covered by mouse cCREs. **j-l,** Scatter plots as described in (**d-f**) for mouse datasets.

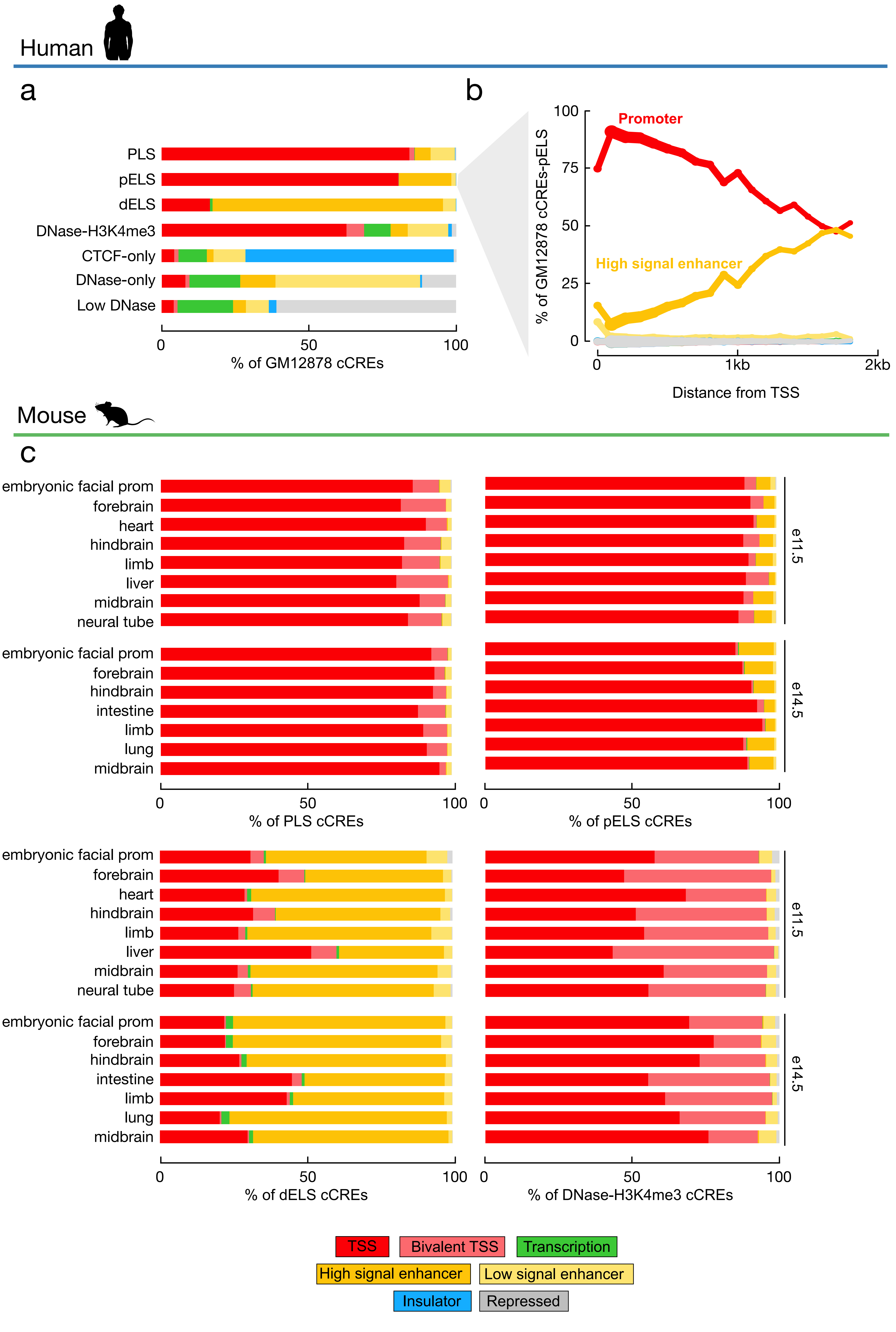

Supplementary Figure 8 | Overlap of cCREs with ChromHMM states. a, Percentages of various groups of GM12878 cCREs that overlap ChromHMM states. **b,** Percentage of GM12878 cCREs-pELS that overlap ChromHMM states ranked by distance from the nearest TSS. Due to ChromHMM's lower spatial resolution, cCREs-PLS that are closest to TSSs overlap promoter ChromHMM states while those farther away overlap enhancer states. **c,** Percentages of mouse cCREs that overlap ChromHMM states in the corresponding tissue. All combinations of tissues and timepoints with both DNase and histone modification data were included and the overlap was computed for data in the same tissue at the same time point.

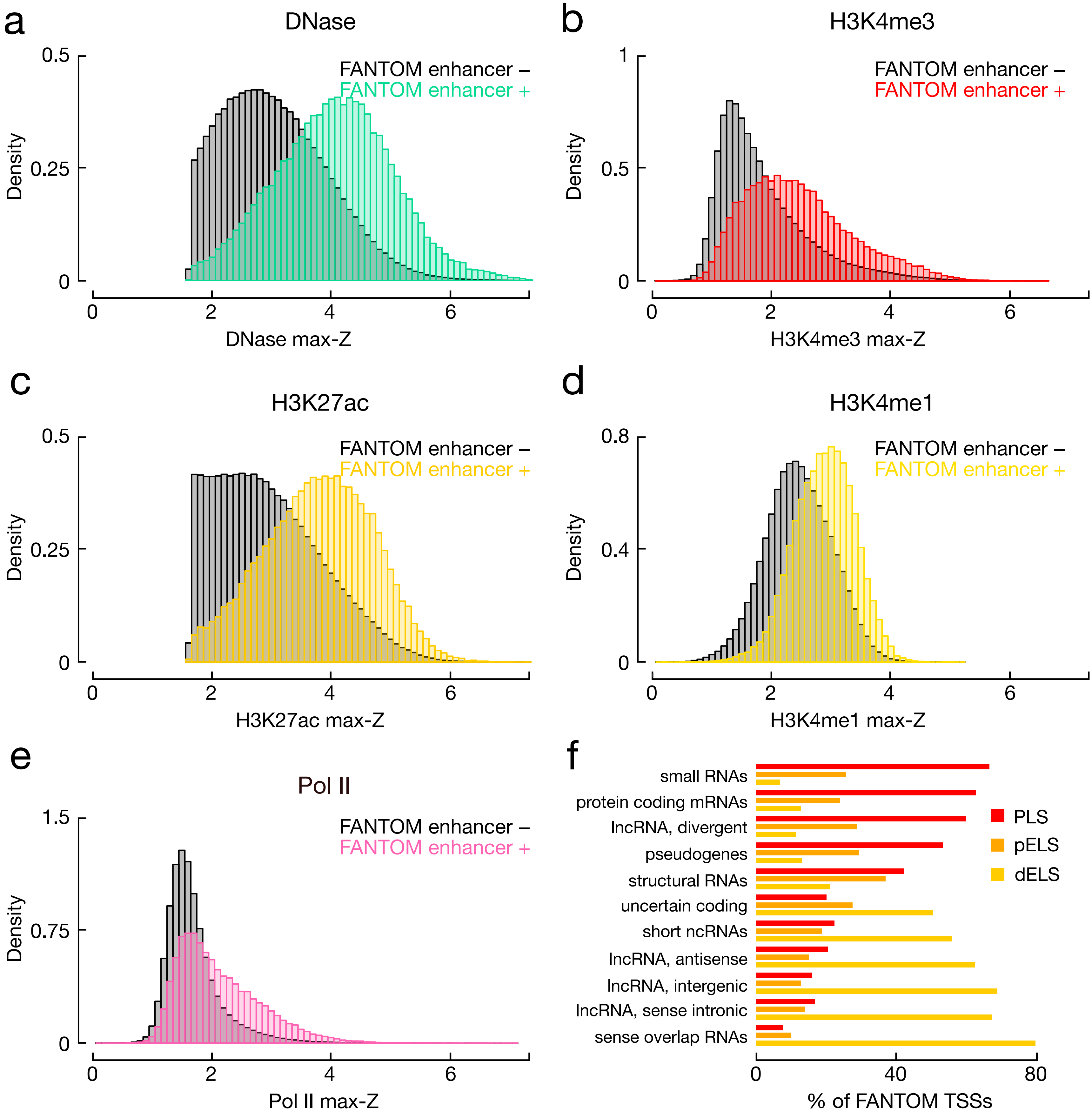

Supplementary Figure 9 | Overlap of cCREs with FANTOM enhancers and the transcription start sites of FANTOM CAGE-associated transcripts. Histograms of the Z-scores of cCREs intersecting FANTOM enhancers (colored) and not intersecting FANTOM enhancers (gray). Z-scores are plotted for **a,** DNase; **b,** H3K4me3; **c,** H3K27ac; **d,** H3K4me1; and **e,** Pol II. **f,** Percentages of the transcription start sites of FANTOM CAGE-associated transcripts in the eleven FANTOM-defined categories that overlap cCREs-PLS (red), cCREs-pELS (orange), or cCREs-dELS (yellow). The TSSs of the majority of coding-associated transcripts (protein coding mRNA and divergent lncRNAs) overlapped a cCRE-PLS, while the TSSs of the majority of eRNA-like non-coding RNAs (short ncRNAs, antisense lncRNAs, intergenic lncRNAs, sense intronic lncRNAs, and sense overlap RNAs) overlapped a cCRE-dELS.

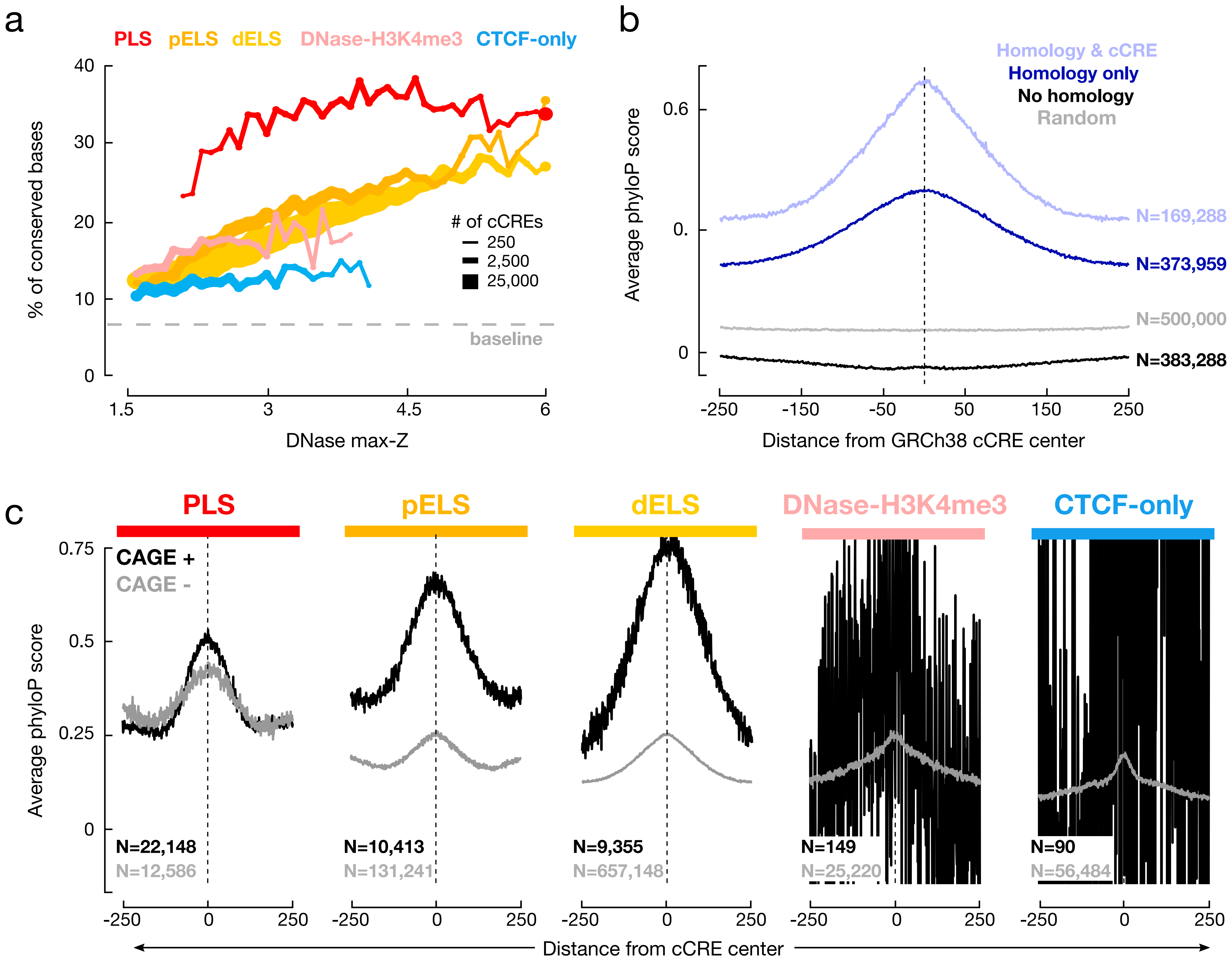

Supplementary Figure 10 | Conservation of human cCREs. a, The percentage of positions of PLS (red), pELS (orange), dELS (yellow), DNase-H3K4me3 (pink) and CTCF-only (blue) cCREs that overlap the GERP++ set of evolutionarily conserved regions binned by their DNase max-Z score. Bins with fewer than 20 cCREs are omitted. **b,** Average phyloP scores of human cCREs stratified by homology categories defined in Extended Data Fig. 2b. **c,** Average phyloP scores across human cCREs stratified by cCRE group and presence of a FANTOM CAGE peak. cCREs that overlap CAGE peaks are designated by black lines while cCREs that do not overlap CAGE peaks are designated by gray lines.

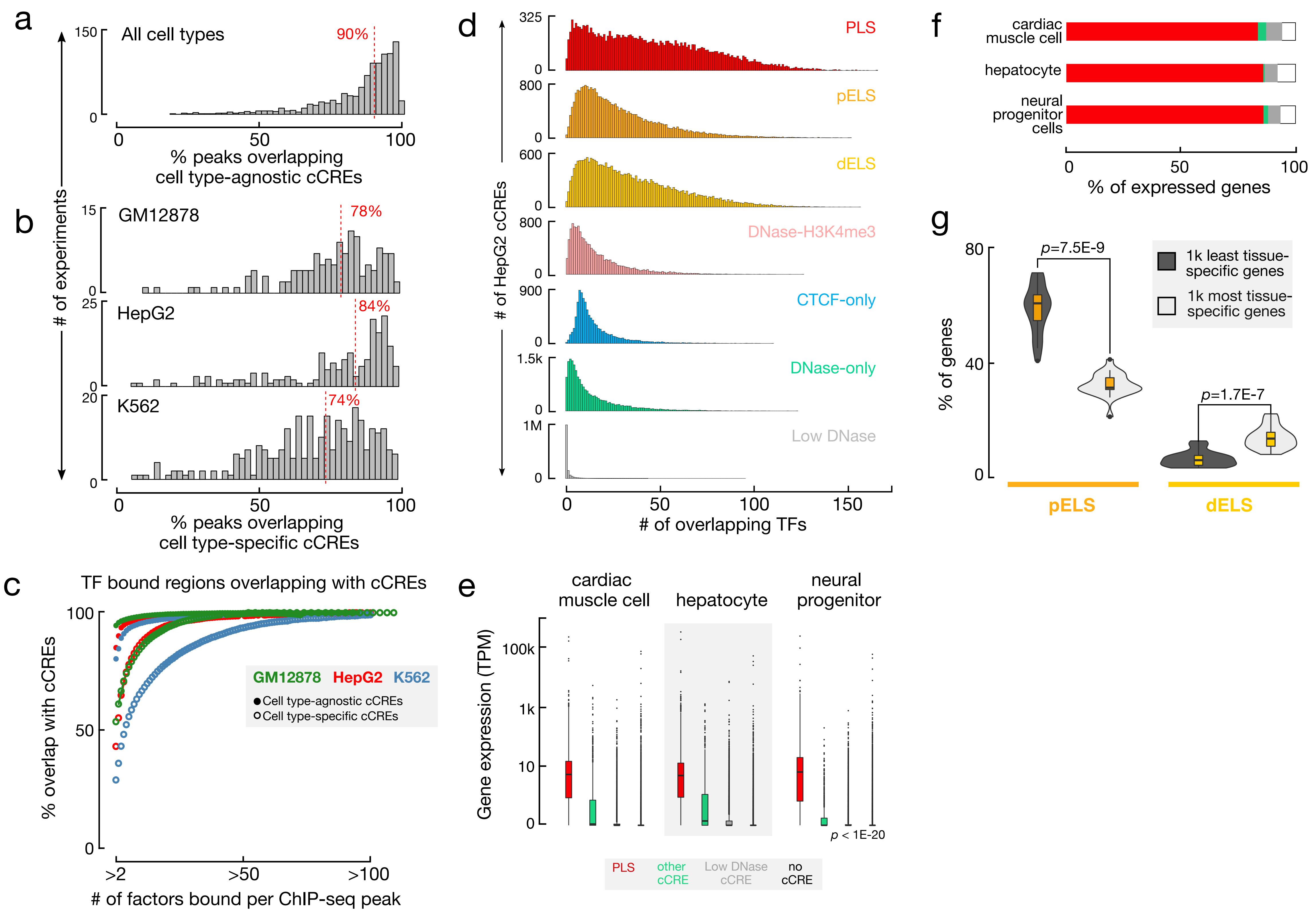

Supplementary Figure 11 | Comparison of cCREs with the ChIP-seq peaks of chromatin-associated proteins and RNA-seq data. a, The vast majority of high-quality ChIP-seq peaks of chromatin-associated proteins overlap cell type-agnostic cCREs. The median overlap is 90% across all ChIP-seq experiments. **b,** The overlap remains high for ChIP-seq peaks with cCREs predicted to be active in the same cell type: median overlap is 78%, 84% and 74% in GM12878, HepG2, and K562 cells, respectively. **c,** Overlap of cell type-agnostic (fill circles) and cell type-specific cCREs (open circles) with ChIP-seq peaks of chromatin-associated proteins in GM12878 (green), HepG2 (red), and K562 (blue) stratified by the number of bound proteins per peak. **d,** Histograms depicting the number of ChIP-seq peak summits contained within each cCRE stratified by cCRE classification in HepG2. **e,** Expression levels of four sets of genes classified by whether their TSSs overlap a PLS (N=16,256, 15,767, and 16,491) other high-DNase cCRE (N=1,183, 2,003, and 938) low-DNase cCRE (N=10,280, 9,949, and 10,290) or no cCRE (N=32,835) in cardiac muscle cells, hepatocytes, and neural progenitors, respectively. Genes with TSSs that overlap cCREs-PLS are more highly expressed (pairwise two-sided Wilcoxon tests, $p < 1E-20$). Boxplots denote median and quartiles. **f,** Percent of expressed genes (TPM > 1) with TSSs that overlap each cCRE group in cardiac muscle cells, hepatocytes, and neural progenitor cells. **g,** Percent of the least tissue-specific (dark gray) and the most tissue-specific (light gray) genes with a nearby (within 10 kb) cCRE-pELS (orange) or cCRE-dELS (yellow) across 23 biosamples with matching data. The least tissue-specific genes are more likely to have a nearby pELS (paired two-sided Wilcoxon test, $p = 7.5E-9$), while the most tissue-specific genes are more likely to have a nearby dELS ($p = 1.7E-7$) defined in the same biosample. Violin plots represent entire distribution and boxplots denote median and quartiles.

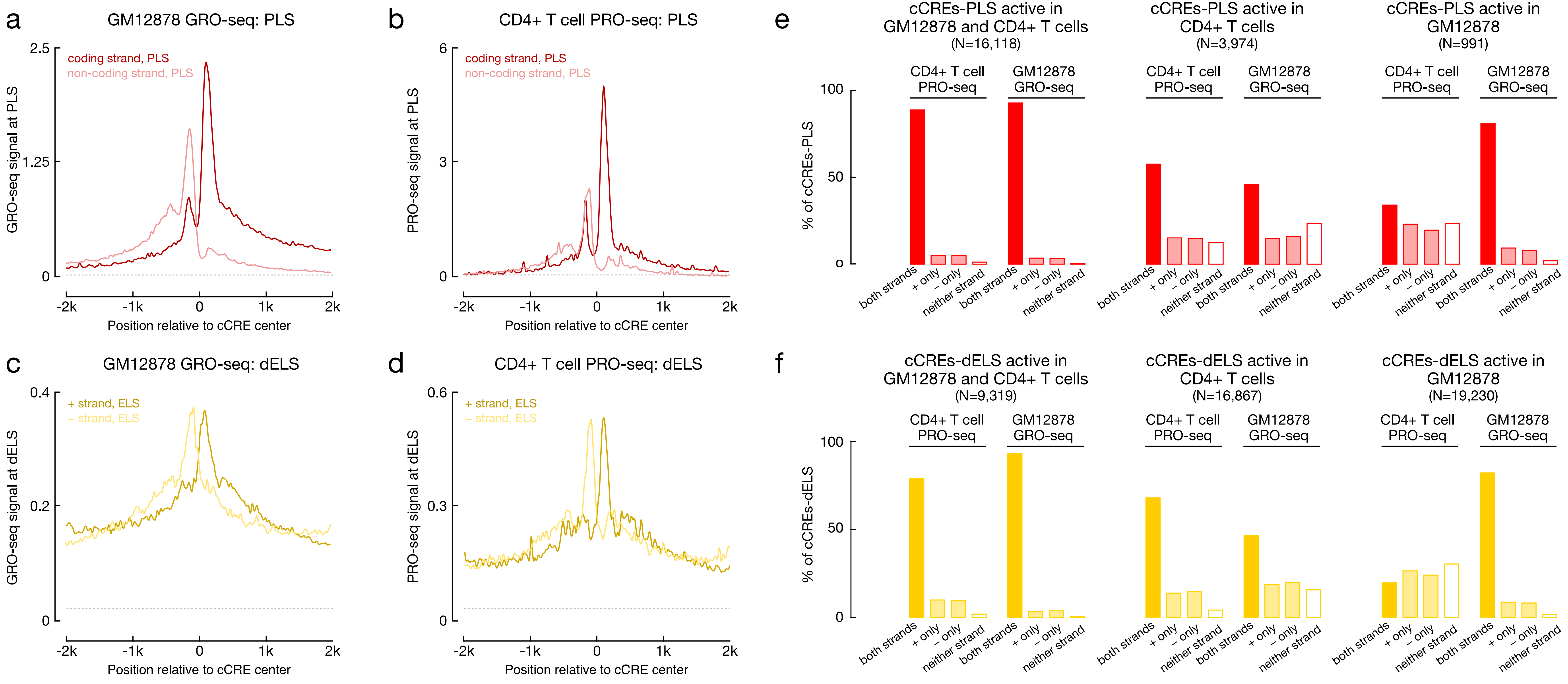

Supplementary Figure 12 | Transcription patterns at cCREs. a-d, GRO-seq signal in GM12878 (**a, c**) and PRO-seq signal in CD4+ T cells (**b, d**) averaged over all cCREs-PLS (**a-b**, in red) and cCREs-dELS (**c-d**, in yellow) in a ± 2 kb window centred on cCRE centers. The GRO-seq and PRO-seq signals around cCREs-PLS were grouped by the orientation of their associated genes. The GRO-seq and PRO-seq signals around cCREs-dELS were grouped by genomic strands. Genomic background signal, computed as described in Supplemental Methods, is shown by the grey dashed line and was approximately 0.02 for both strands in GM12878 and 0.03 for both strands in CD4+ T-cell. **e-f,** The percentage of cCREs-PLS (**e**) and cCREs-dELS (**f**) with GRO-seq or PRO-seq signal > 0 on both strands (solid bars), the plus strand only or the minus strand only (light-color bars), or neither strand (open bars) in both GM12878 and CD4+ T cells (left set of bars in each plot), CD4+ T cells only (the middle set of bars in each plot), and in GM12878 cells only (the right set of bars in each plot).

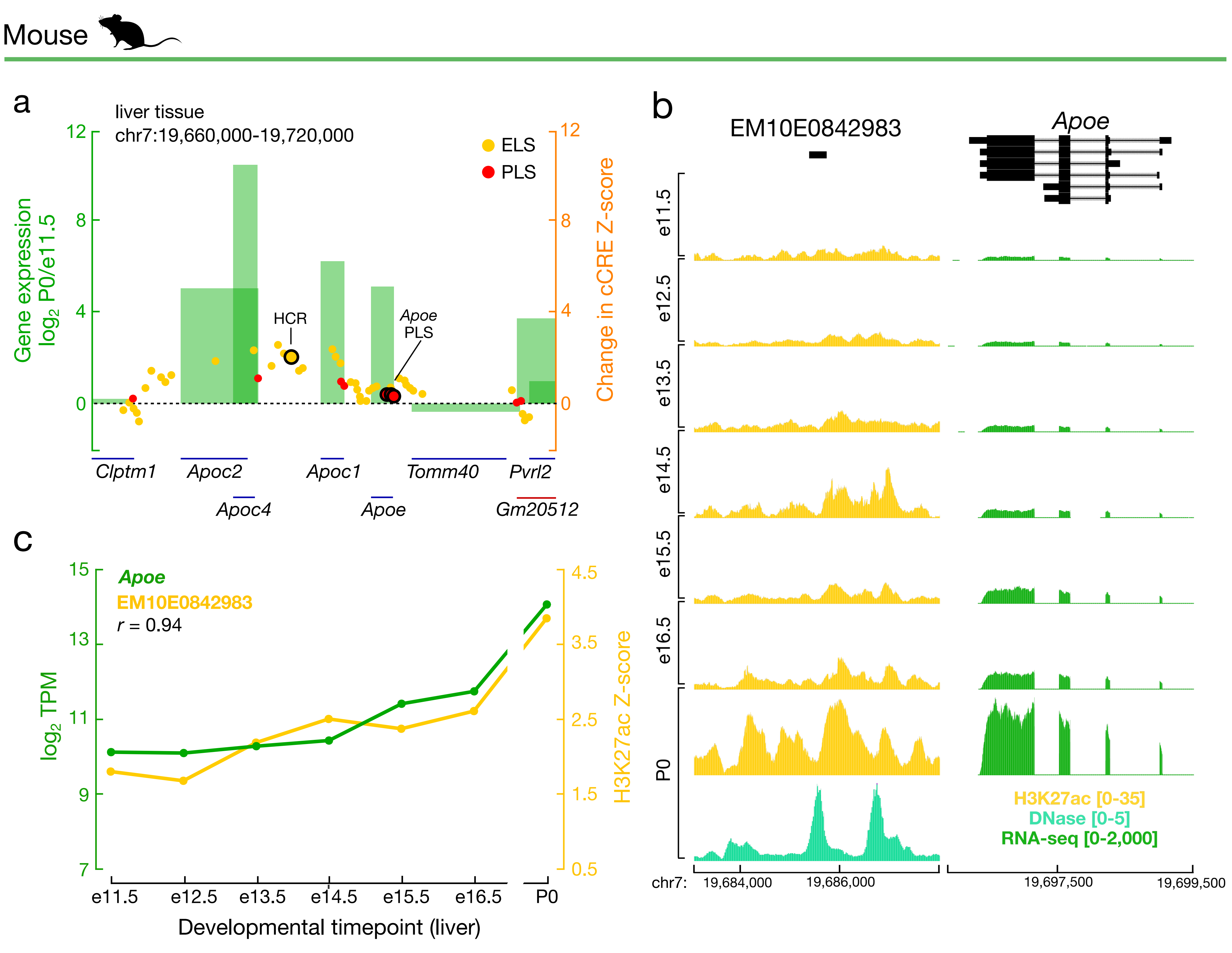

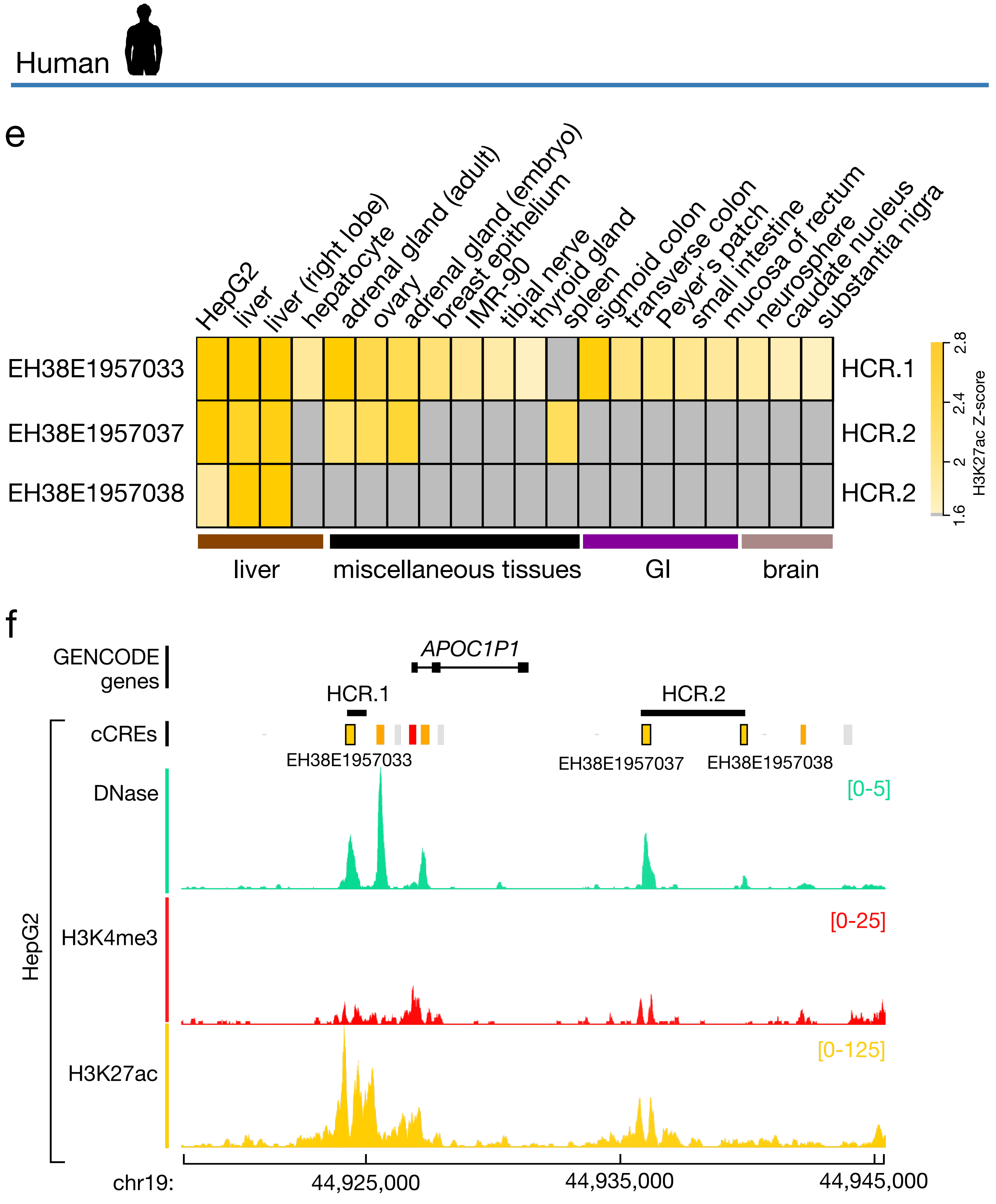

d

EM10E0842983

--- indicates no data

Biosample	H3K27ac Z-score	DNase Z-score
liver P0	3.87	2.29
liver (adult 8 weeks)	3.13	---
liver e16.5	2.63	---
liver e14.5	2.52	1.72
embryonic stem cells	2.47	1.71
liver e15.5	2.39	---
liver e13.5	2.20	---
spleen (adult 8 weeks)	2.08	-0.84
liver e11.5	1.82	1.57
liver e12.5	1.69	---

Supplementary Figure 13 | Analyzing differential gene expression and differential cCRE activity across mouse developmental timepoints. a, Comparison between liver e11.5 and P0 gene expression and cCRE activity at the *Apoe* locus. Green bars indicate differentially expressed genes, and red and yellow dots indicate cCREs-PLS and cCREs-ELS. The widths of the green bars represent gene lengths. The lines beneath the green bars and above the gene names indicate the positions and orientations of the genes—red for plus genomic strand and blue for minus strand. The heights of bars or dots indicate changes—$\log_2$(fold change) or difference in Z-score—between the two timepoints. The cCRE-dELS EM10E0842983 that overlaps a hepatic control region (HCR) is outlined in black. **b,** A genome browser view of the *Apoe* locus with H3K27ac, DNase, and RNA-seq signals in the liver across all surveyed timepoints. **c,** *Apoe* gene expression and EM10E0842983 H3K27ac level increase coordinately during development (Pearson's correlation across seven timpoints: r = 0.94). **d,** H3K27ac and DNase Z-scores in mouse biosamples at EM10E0842983. **e,** H3K27ac activity in human biosamples for EH38E1957033 (HCR.1), EH38E1957037 (HCR.2) and EH38E1957038 (HCR.2). Biosamples are grouped by tissue ontology. **f,** A genome browser view of HCR.1 and HCR.2 in human, their overlapping cCREs, and epigenomic signals in HepG2 cells.

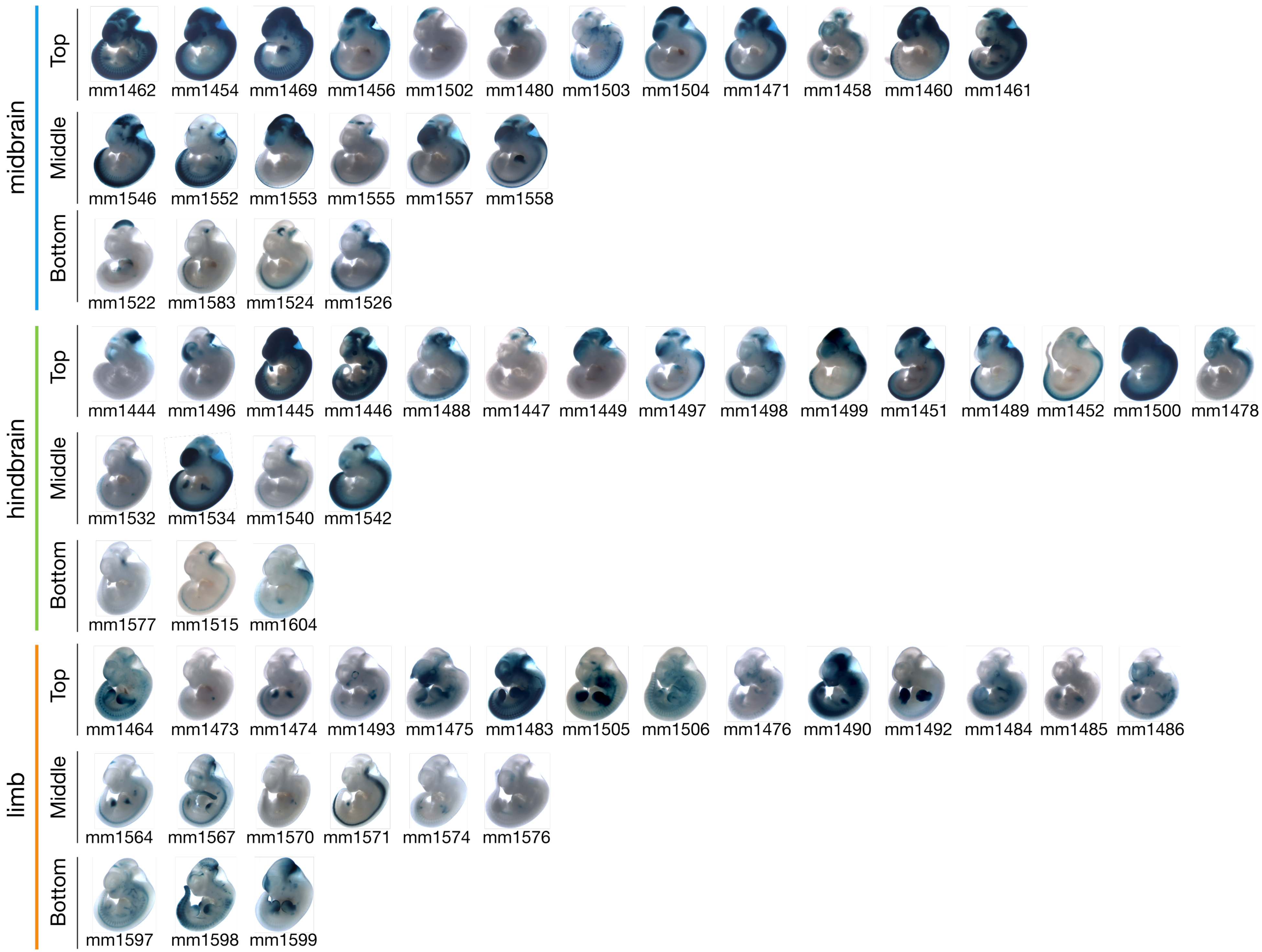

Supplementary Figure 14 | Results of testing cCREs-ELS using the in vivo transgenic mouse assays. Shown are representative staining images of e11.5 transgenic mouse embryos (with darker staining indicating higher enhancer activity) for the predicted enhancers that displayed reproducible activity (positive in at least three embryos) in the expected tissue (midbrain, hindbrain, or limb). Enhancer predictions were performed using the average rank of H3K27ac and DNase signals in the respective e11.5 tissue, and three tranches of predictions were chosen for testing: Top ranking, Middle ranking (~1,500) and Bottom ranking (~3,000). The unique identifier below each embryo (mm number) corresponds to the accession of the enhancer in the VISTA enhancer browser (https://enhancer.lbl.gov/).

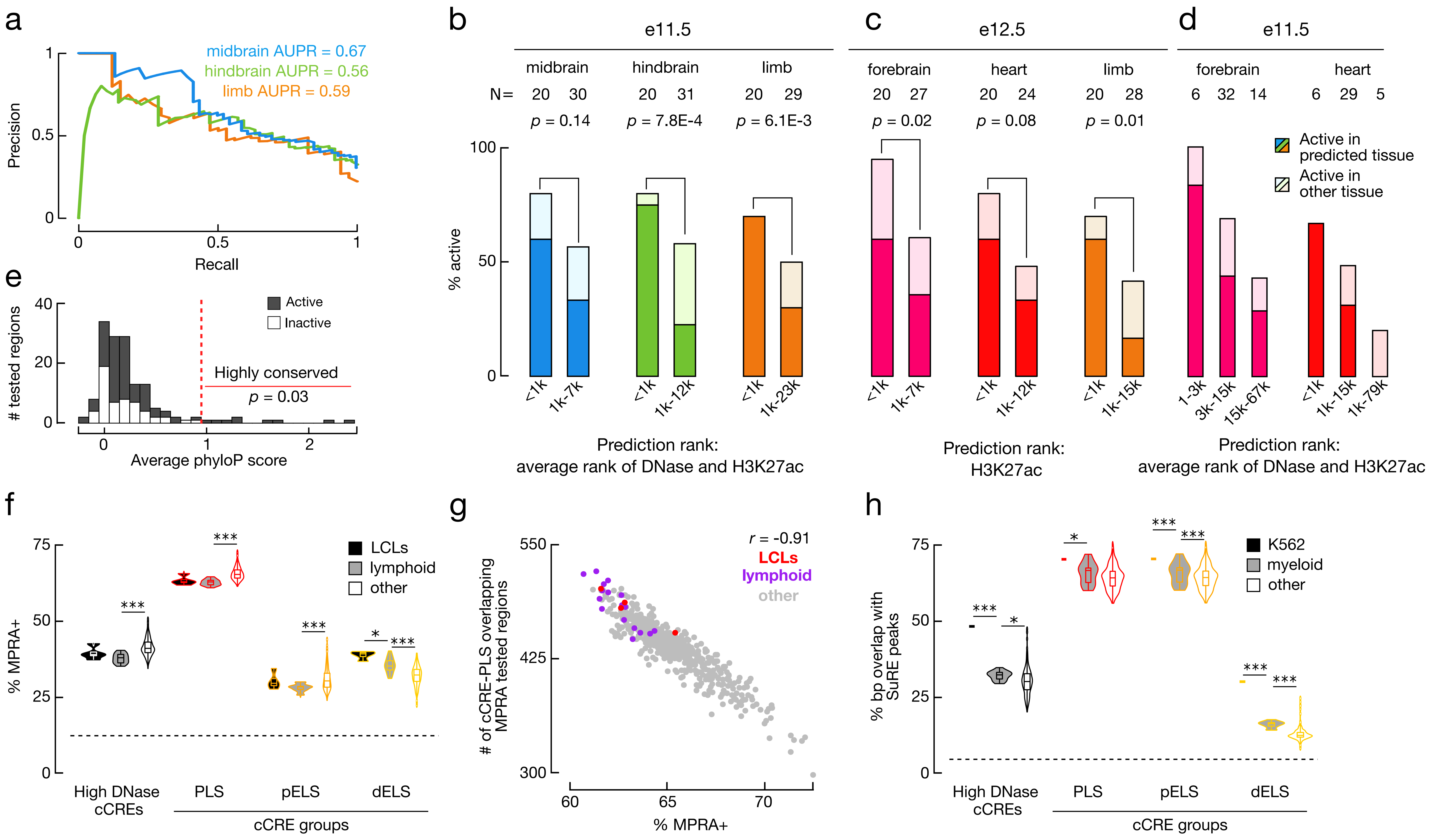

Supplementary Figure 15 | Additional analysis of cCREs tested by transgenic mouse assays and cCREs overlapping regions tested by functional assays MPRA and SuRE. a, PR curves for the 151 cCRE-anchored regions predicted to function as enhancers in e11.5 midbrain, hindbrain, or limb, respectively. **b-d,** Bar plots of the overlap of regions tested by transgenic enhancer assays in this paper (**b**), Gorkin *et al.* (**c**), and Sethi *et al.* (**d**). Bars are separated into groups by the ranks of the overlapping cCREs. The best cCRE rank for the tested e11.5 forebrain regions was 1,259; hence, the regions were divided using cCRE rank 3,000. Dark colors indicate activity in the predicted tissue, while light colors indicate activity in non-predicted tissues. Two-sided Fisher's exact test *p*-values are shown for comparing activity in the predicted tissue. Due to the small number of regions in the Sethi set (**d**), the comparisons between the groups are not statistically significant ($p > 0.05$). **e,** Stacked histogram displaying the average phyloP score across the 151 tested regions colored dark gray if active or white if inactive. If we only select highly conserved regions (average phyloP > 1), we observe significant enrichment for positive regions ($p = 0.03$, Fisher's exact test). **f,** Overlap of cCREs with MPRA+ regions tested in GM12878 cells (N=3,103) stratified by cCRE group and biosample origin: LCLs (black, N=4), lymphoid lineage (gray, N=17), other (white, N=496). Violin plots display entire distribution with boxplots denoting median and quartiles. P-values are shown for two-sided Wilcoxon tests with * indicating $p < 0.05$ and *** indicating $p < 0.001$. **g,** Scatterplot of the percent of MPRA+ regions compared to the total number of overlapping MPRA tested regions (N=25,295). Each point is a biosample (N=517) colored by whether it is an LCL (red), from the lymphoid lineage (purple), or other (gray). LCLs and lymphoid samples have a lower percentage of MPRA+ regions because they overlap more tested regions due to ascertainment bias (Pearson correlation coefficient). **h,** Overlap of cCREs with SuRE peaks (N=55,454) from K562 cells stratified by cCRE group and biosample origin: K562 (black, N=1), myeloid lineage (gray, N=16), other (white, N=500). Violin plots display entire distribution with boxplots denoting median and quartiles. P-values are shown for two-sided Wilcoxon tests with * indicating $p < 0.05$ and *** indicating $p < 0.001$.

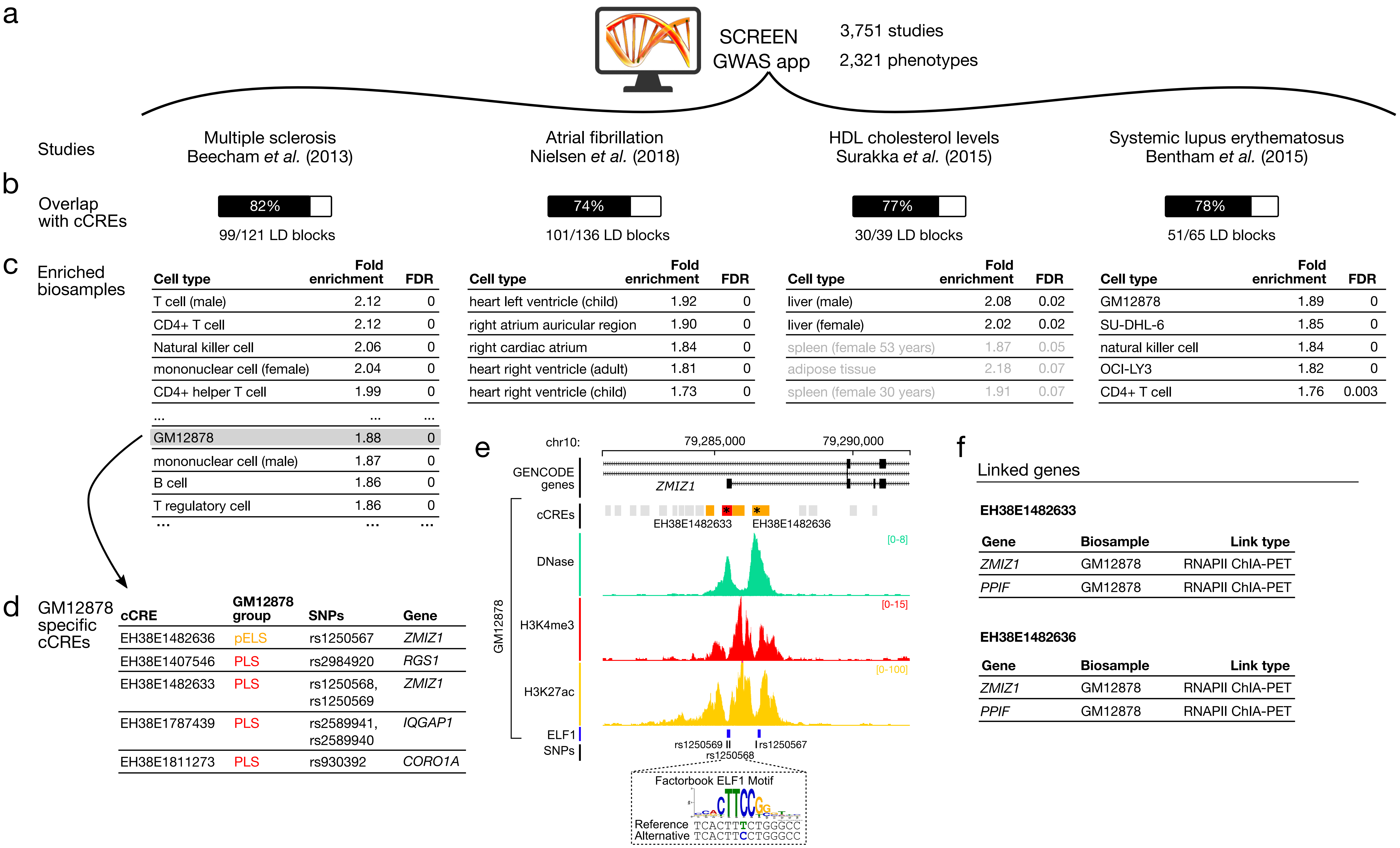

Supplementary Figure 16 | Annotating GWAS variants using cCREs. a, Users can select a GWAS from a preloaded list of GWAS in SCREEN. For each study, we included all tag SNPs reported in the study and all SNPs in LD with the tag SNPs ($r^2 > 0.7$). **b,** SCREEN reports the percentage of LD blocks of a GWAS with at least one SNP overlapping a cCRE. **c,** Biosamples are ranked by enrichment of SNP-overlapping cCREs with high H3K27ac signals compared to 500 controls. Top cell and tissue types are displayed here for each study. P-values were calculated from Z-scores (two-sided) and we calculated an FDR to correct for multiple testing. For a GWAS (e.g., multiple sclerosis), the user can narrow the search by selecting a biosample (e.g., GM12878 with a 1.88 fold enrichment) and analyze the overlapping cCREs. **d,** Three multiple sclerosis SNPs overlap GM12878 cCREs proximal to a *ZMIZ1* TSS, with rs1250567 overlapping a pELS (EH38E1482636) and rs1250568 and rs1250569 overlapping a PLS (EH38E1482633). **e,** The two cCREs in **d** (in asterisks) have high DNase, H3K4me3, and H3K27ac signals in GM12878 and overlap the ChIP-seq peaks of the transcription factor ELF1. In particular, rs1250568 overlaps a high-scoring position of the ELF1 motif (in the box), with the reference allele disrupting the motif. **f,** ChIA-PET data link EH38E1482633 and EH38E1482636 to *ZMIZ1* and *PPIF* in GM12878.

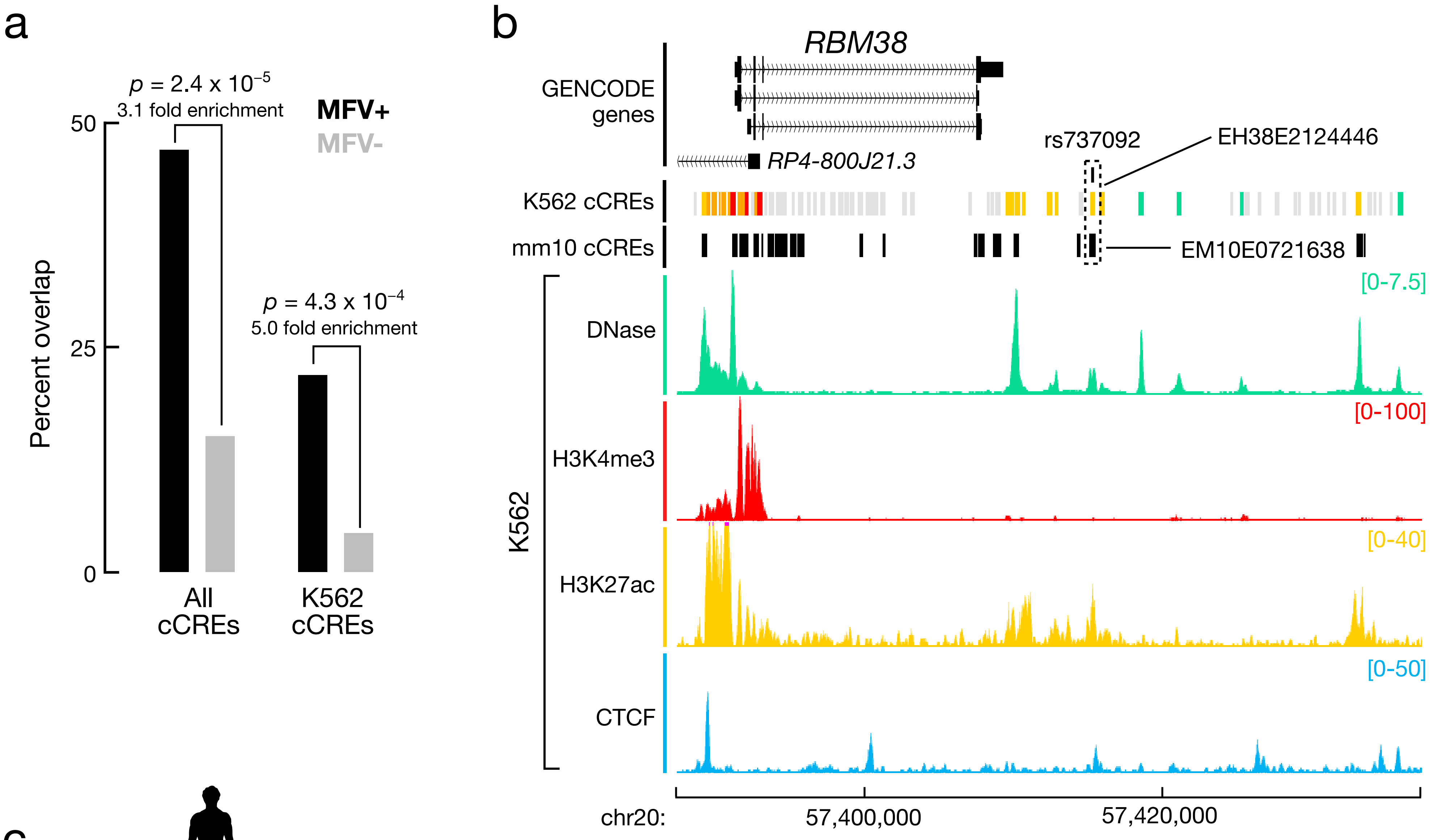

c

Human EH38E2124446

Biosample	DNase Z-score
hematopoietic multipotent progenitor (x 8)	7.27
liver (embryonic)	3.22
common myeloid progenitor (x 9)	3.21
NB4	2.87
BE2C	2.82
CMK	2.78
H4	2.60
K562	2.36
M059J	2.30
RPMI-7951	2.26

Biosample	H3K27ac Z-score
CD8+ α-β memory T cell	2.38
K562	2.33
UCSF-4	2.15
CD4+ α-β memory T cell	2.14
cardiac muscle cell	1.68
rectal smooth muscle tissue	1.59
common myeloid progenitor	1.47
stomach smooth muscle	1.43
ovary	1.34
CD14+ monocyte	1.22

d

Mouse EM10E0721638

Biosample	DNase Z-score
129.DLCR liver e14.5	4.88
129 liver e14.5	4.83
C57BL/6 liver e14.5	4.78
CD117-,CD71+,TER119+ erythroid progenitor	4.69
CD117+,CD71+,TER119+ erythroid progenitor	4.13
DBA/2 erythroblast estrodial treament 2 day	3.63
CD117+,CD71+,TER119- erythroid progenitor	3.25
DBA/2 erythroblast estrodial treament 1 day	3.08
C57BL/6 liver P0	2.95
C57BL/6 thymus P0	2.88

Biosample	H3K27ac Z-score
C57BL/6 liver e15.5	4.96
C57BL/6 liver e13.5	4.79
C57BL/6 liver e16.5	4.65
C57BL/6 liver e12.5	4.65
C57BL/6 liver e11.5	3.77
C57BL/6 liver e14.5	3.60
C57BL/6 liver P0	3.47
C57BL/6 bone marrow adult 8 weeks	3.12
DBA/2 MEL	2.47
C57BL/6 spleen adult 8 weeks	2.47

Supplementary Figure 17 | Using cCREs to annotate functional SNPs related to red blood cell traits. a, Overlap of cCREs with MPRA functional variants (MFV+, N=32) and non-functional variants (MFV–, N=2,724) associated with red blood cell traits. MFV+ regions overlap cCREs (N=926,535) more frequently than MFV- regions (3.1 fold enrichment, two-sided Fisher's exact test, $p = 2.4 \times 10^{-5}$), especially K562 cCREs (N=96,385, 5.0 fold enrichment, $p = 4.3 \times 10^{-4}$). **b,** Genome browser view of RBC SNP rs737092, which is downstream of the *RBM38* gene. This variant overlaps a human cCRE-ELS (EH38E2124446), which has a high DNase signal (green) and a high H3K27ac signal (yellow) in K562. rs737092 also overlaps a homologous mouse cCRE (EM10E0721638, black). **c,** Top ten cell and tissue types ranked by Z-score for DNase (left) and H3K27ac (right) at EH38E2124446. Multiplicative indices indicate multiple biosamples. **d,** Top ten mouse biosamples ranked by Z-score for DNase (left) and H3K27ac (right) at EM10E0721638.

a

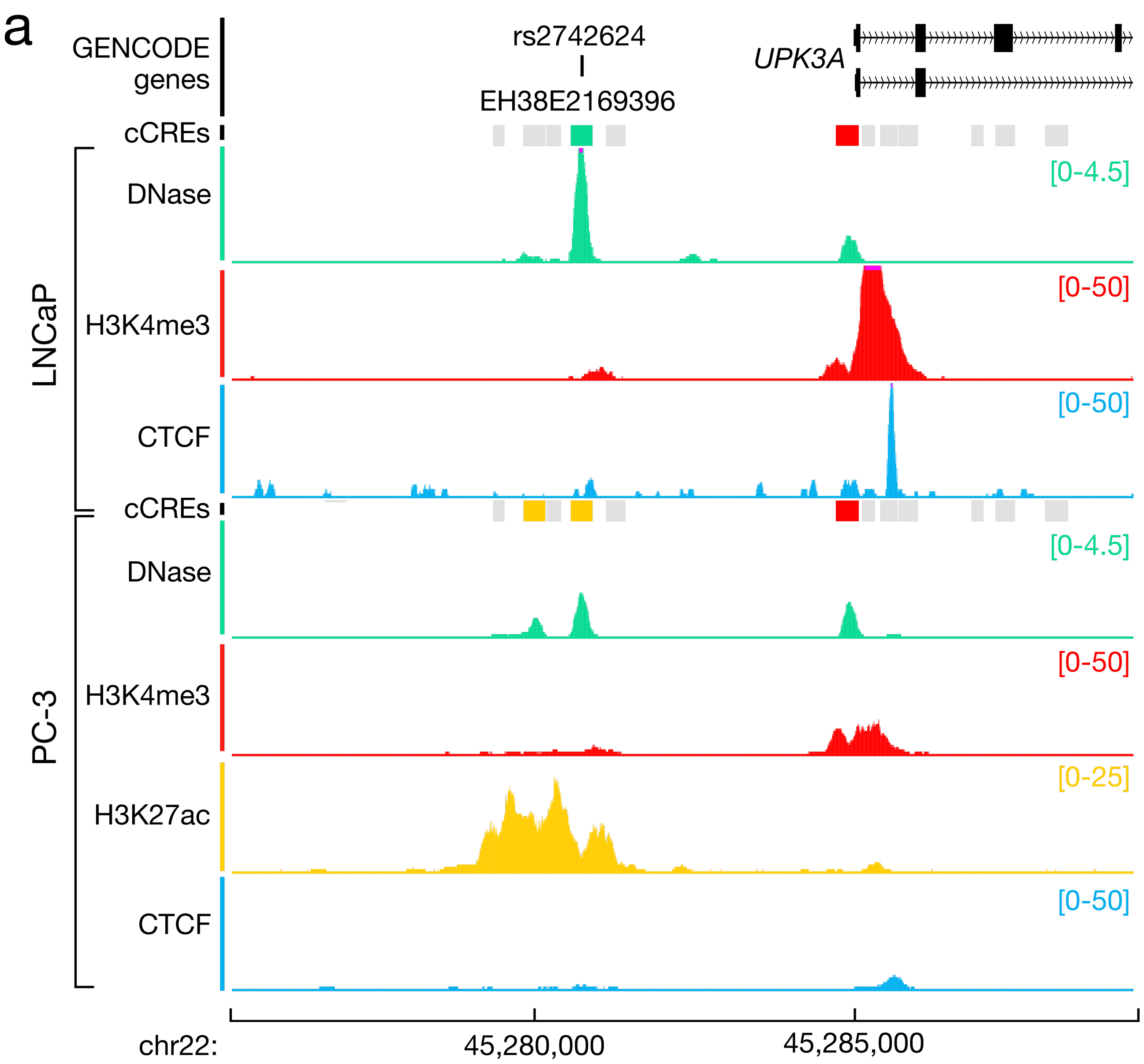

b

Biosample	EH38E2169396 DNase Z-score
LNCaP*	3.45
PC-9	3.33
HT-29	2.69
large intestine	2.63
PC-3*	2.57
large intestine	2.47
T47D	2.36
large intestine	2.33
large intestine	2.32
MCF-7	2.30

c

Biosample	EH38E2169396 H3K27ac Z-score
VCaP*	3.79
prostate gland	3.61
thyroid gland	3.42
PC-9	3.33
HepG2	3.27
MCF-7	3.14
PC-3*	3.06
neutrophil	3.03
parathyroid adenoma	3.00
large intestine	2.93

* prostate cancer cell lines

d

Gene	Biosample	Link type
FAM118A	nerve, blood	eQTL
FAM118A	IMR-90	Hi-C
FBLN1	NHEK	Hi-C
UPK3A	brain, skeletal muscle, pituitary, prostate, small intestine, blood	eQTL

Supplementary Figure 18 | Using cCREs to annotate functional SNPs related to prostate cancer. a, Genome browser view of rs2742624, which was previously shown to affect the expression of *UPK3A*, with epigenomic signals of the overlapping cCRE EH38E2169396 in LNCaP and PC-3 (prostate cancer cell lines). **b-c,** Top ten biosamples ranked by Z-score for (**b**) DNase and (**c**) H3K27ac at EH38E2169396. **d,** Genes linked to EH38E2169396 by eQTLs and Hi-C interactions.

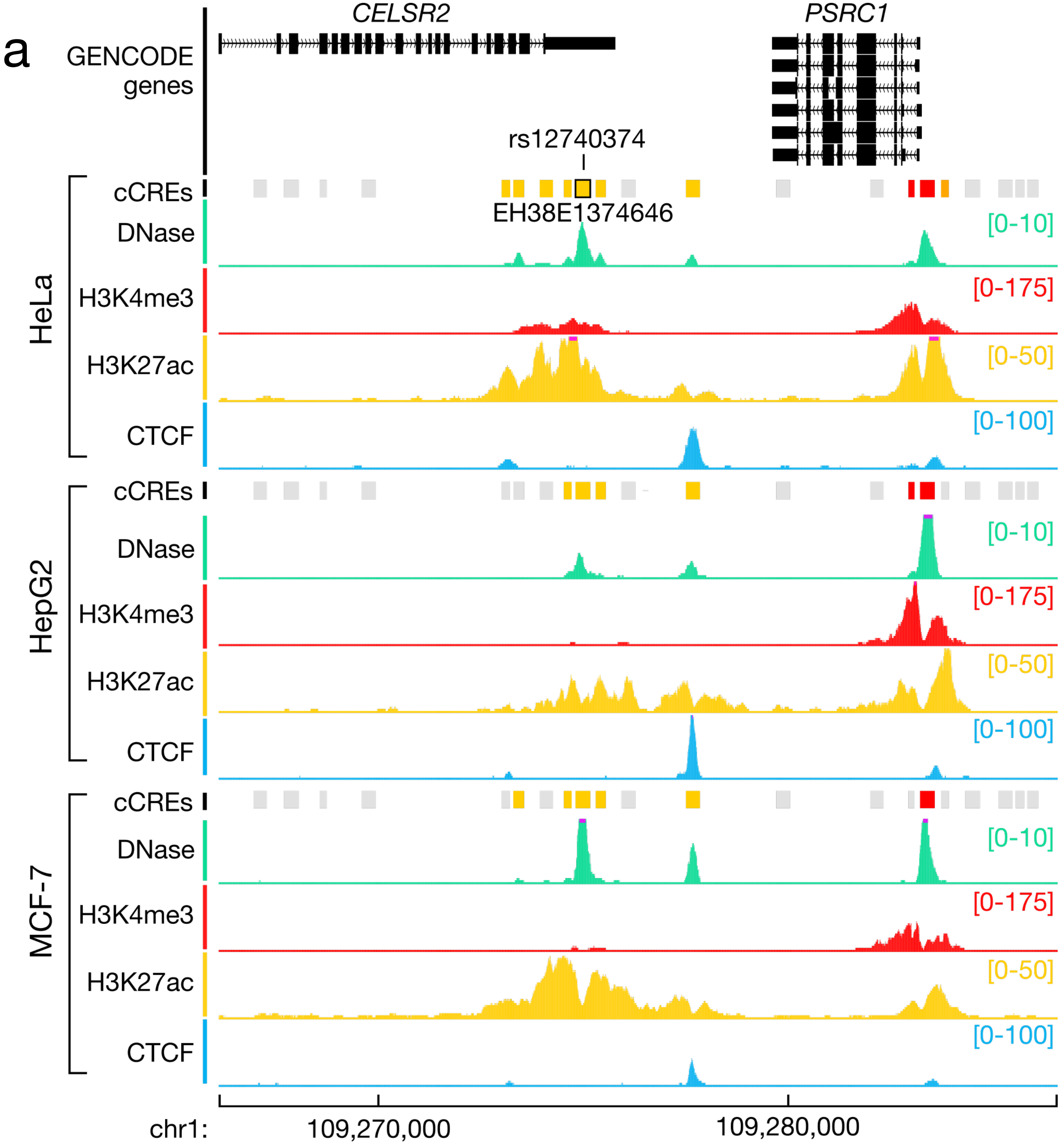

b

Biosample	EH38E1374646 DNase Z-score
mammary epithelial cell	4.10
A549	4.06
HeLa	3.95
T47D	3.94
MCF-7 w/ estrodial	3.92
prostate epithelial cell	3.89
foreskin melanocyte	3.83
MCF-7	3.81
HepG2	3.79
kidney tubule	3.77

c

Biosample	EH38E1374646 H3K27ac Z-score
MCF-7	4.55
PC-3	4.10
trophoblast	3.97
A549 w/ ethanol	3.97
prostate epithelial	3.85
HeLa	3.69
placental basal plate	3.49
substantia nigra	3.36
thyroid gland	3.34
foreskin keratinocyte	3.28

d

Gene	Biosample	Link type
AMIGO1	HUVEC	Hi-C
CELSR2	esophagus, skeletal muscle, skin	eQTL
CELSR2	HeLa	CTCF ChIA-PET
PSRC1	artery, brain, esophagus, heart, skin, skeletal muscle, nerve, testis, blood	eQTL
PSRC1	HeLa	CTCF ChIA-PET
SORT1	esophagus	eQTL
SORT1	HeLa	CTCF ChIA-PET
SORT1	K562	POLR2A ChIA-PET

Supplementary Figure 19 | Using cCREs to annotate functional SNPs related to liver traits. a, Genome browser view of rs12740374 with epigenomic signals from three biosamples demonstrating the ubiquitous activity of EH38E1374646. **b-c,** Top ten biosamples ranked by Z-score for (**b**) DNase and (**c**) H3K27ac at EH38E1374646. **d,** Genes linked to EH38E1374646 by eQTLs, Hi-C, and ChIA-PET interactions.

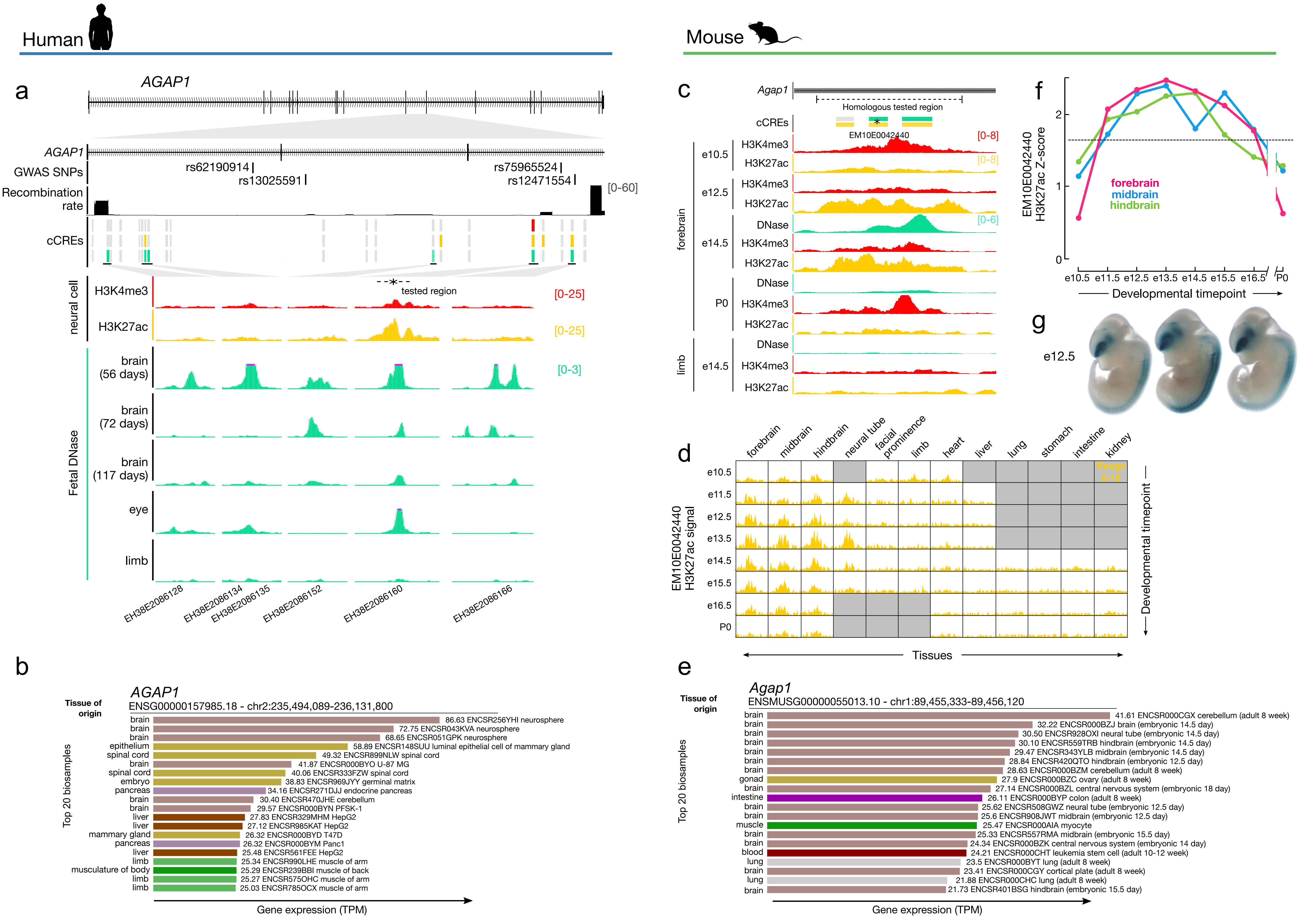

Supplementary Figure 20 | Interpreting GWAS variants associated with neuropsychiatric disorders using cCREs. a, An intron of the *AGAP1* gene contains several SNPs associated with neuropsychiatric phenotypes. This region has high linkage disequilibrium and contains many cCREs with high DNase signals in the fetal brain. One region of particular interest also has high H3K4me3 and H3K27ac signals in neural cells, suggesting that it is an enhancer in neural cells. We further analyzed a segment of this region that contains cCRE EH38E2086160 (dashed line with an asterisk). **b,** Expression of *AGAP1* in human biosamples. *AGAP1* is primarily expressed in the adult brain. Expression values were calculated from whole-cell poly-A RNA-seq experiments and are displayed in transcripts per million (TPM). **c,** The mouse region in the *Agap1* locus that is homologous to the EH38E2086160-containing human region, also indicated by a dashed line, contains three cCREs with high H3K27ac and DNase signals in the fetal mouse forebrain but not the limb. The mouse cCRE EM10E0042440 is homologous to the human cCRE EH38E2086160. **d,** EM10E0042440's H3K27ac signals measured as fold change between ChIP and input are displayed across 12 mouse fetal tissues at 8 developmental timepoints. Tissues without H3K27ac ChIP-seq data are in gray. The maximal height of the signals is 10. **e,** Expression of *Agap1* in mouse. *Agap1* is primarily expressed in fetal brain tissues. Expression values were calculated from whole-cell poly-A RNA-seq experiments and are displayed in TPM. **f,** EM10E0042440's H3K27ac signal in the forebrain (pink), midbrain (blue), and hindbrain (green) across mouse fetal development. **g,** Results from transgenic mouse assays testing the human region indicated with a dashed line in **a**, which is homologous to the region also indicated with a dashed line in **c.** Images indicate that this region is active in mouse e12.5 brain tissues, with three stained mouse embryos shown. Of the ten tested embryos, all ten tested positive for enhancer activity.

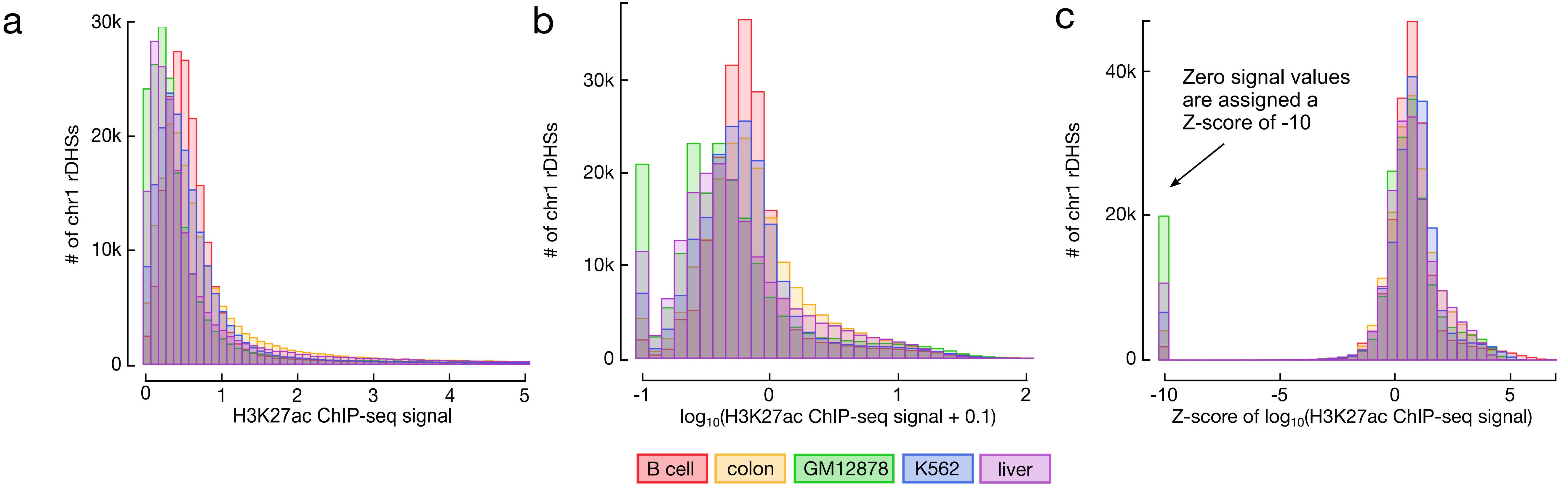

Supplementary Figure 21 | Method for normalizing epigenomics signals. a, Distribution of the H3K27ac signals of the rDHSs on chromosome 1 in five biosamples (B cell, colon, GM12878, K562, and liver; shown in different colors). rDHSs with H3K27ac signals higher than 5 (average of 9.3k rDHSs per biosample) are omitted from this histogram. **b,** Distributions of the $\log_{10}$ of the H3K27ac signals in **a**. The $\log_{10}$(signal) values of the rDHSs in each biosample roughly follow a normal distribution. **c,** Distribution of the Z-scores corresponding to the $\log_{10}$(signal) values in **b**, with a Z-score of –10 assigned to zero signal values.
