# Supplementary material for: Expanded encyclopaedias of DNA elements in the human and mouse genomes
Source: Nature. 2020 Jul 29;583(7818):699–710. doi: 10.1038/s41586-020-2493-4 (PMC7410828; doi:10.1038/s41586-020-2493-4)
Supplement: Supplementary file 2 — Reporting Summary [file 41586_2020_2493_MOESM2_ESM.pdf]

nature research

Corresponding author(s): Zhiping Weng

Last updated by author(s): Dec 10, 2019

Reporting Summary

Nature Research wishes to improve the reproducibility of the work that we publish. This form provides structure for consistency and transparency in reporting. For further information on Nature Research policies, see Authors & Referees and the Editorial Policy Checklist.

Statistics

For all statistical analyses, confirm that the following items are present in the figure legend, table legend, main text, or Methods section.

n/a	Confirmed	
☐	☒	The exact sample size (*n*) for each experimental group/condition, given as a discrete number and unit of measurement
☐	☒	A statement on whether measurements were taken from distinct samples or whether the same sample was measured repeatedly
☐	☒	The statistical test(s) used AND whether they are one- or two-sided<br>*Only common tests should be described solely by name; describe more complex techniques in the Methods section.*
☐	☒	A description of all covariates tested
☐	☒	A description of any assumptions or corrections, such as tests of normality and adjustment for multiple comparisons
☐	☒	A full description of the statistical parameters including central tendency (e.g. means) or other basic estimates (e.g. regression coefficient) AND variation (e.g. standard deviation) or associated estimates of uncertainty (e.g. confidence intervals)
☐	☒	For null hypothesis testing, the test statistic (e.g. *F*, *t*, *r*) with confidence intervals, effect sizes, degrees of freedom and *P* value noted<br>*Give P values as exact values whenever suitable.*
☐	☒	For Bayesian analysis, information on the choice of priors and Markov chain Monte Carlo settings
☐	☒	For hierarchical and complex designs, identification of the appropriate level for tests and full reporting of outcomes
☐	☒	Estimates of effect sizes (e.g. Cohen's *d*, Pearson's *r*), indicating how they were calculated

Our web collection on statistics for biologists contains articles on many of the points above.

Software and code

Policy information about availability of computer code

Data collection	All protocols are described in the Methods and Supplementary Methods sections of the manuscript and available in GitHub.
Data analysis	The nearly six thousand experiments were processed using the applicable ENCODE Processing pipeline, which are extensively documented on the ENCODE portal with pipeline schematics and software versions. All pipelines are also available via GitHub. To create the Registry of cCREs and run subsequent analyses we utilized the following commercial software: Bedtools v2.27.1, PRROC v1.3.1, UCSC Utilities (liftOver, bigWigAverageOverBed), DESeq2 v1.14.1. All custom code is available on GitHub.

For manuscripts utilizing custom algorithms or software that are central to the research but not yet described in published literature, software must be made available to editors/reviewers. We strongly encourage code deposition in a community repository (e.g. GitHub). See the Nature Research guidelines for submitting code & software for further information.

Data

Policy information about availability of data

All manuscripts must include a data availability statement. This statement should provide the following information, where applicable:

- Accession codes, unique identifiers, or web links for publicly available datasets
- A list of figures that have associated raw data
- A description of any restrictions on data availability

All ENCODE data are available at the ENCODE Portal (http://encodeproject.org).

Field-specific reporting

Please select the one below that is the best fit for your research. If you are not sure, read the appropriate sections before making your selection.

☒ Life sciences ☐ Behavioural & social sciences ☐ Ecological, evolutionary & environmental sciences

For a reference copy of the document with all sections, see nature.com/documents/nr-reporting-summary-flat.pdf

Life sciences study design

All studies must disclose on these points even when the disclosure is negative.

Sample size	We performed almost six thousand experiments on nearly 500 biosamples including tissues, primary cells, in vitro differentiated cells, and cell lines. No statistical methods were used to determine sample sizes.
Data exclusions	Each ENCODE experiment is subject to assay specific quality control measurements which are available on the ENCODE portal. To create the Registry of cCREs we selected all released DNase experiments with SPOT score > 0.3. To annotate cCREs, we selected one representative experiment per biosample to account for assay redundancy based on QC metrics.
Replication	The majority of all ENCODE assays require two successful replicates. In cases of biosample scarcity one replicate was performed and these rare cases are clearly labeled at the ENCODE portal. For the mouse transgenic enhancer-reporter assays, a predicted element was scored positive as an enhancer if at least three embryos had identical β-galactosidase staining in the same tissue. Specific testing results for the 151 tested regions can be found in Supplemental Table 13 and at https://enhancer.lbl.gov/.
Randomization	No randomization was performed. This was not a clinical trial and therefore randomization is not relevant.
Blinding	No blinding was performed. This was not a clinical trial and therefore blinding is not relevant.

Reporting for specific materials, systems and methods

We require information from authors about some types of materials, experimental systems and methods used in many studies. Here, indicate whether each material, system or method listed is relevant to your study. If you are not sure if a list item applies to your research, read the appropriate section before selecting a response.

Materials & experimental systems

n/a	Involved in the study	
☐	☒	Antibodies
☐	☒	Eukaryotic cell lines
☒	☐	Palaeontology
☐	☒	Animals and other organisms
☒	☐	Human research participants
☒	☐	Clinical data

Methods

n/a	Involved in the study	
☐	☒	ChIP-seq
☒	☐	Flow cytometry
☒	☐	MRI-based neuroimaging

Antibodies

Antibodies used	The > 3,000 antibodies that were used are listed on the ENCODE portal at https://www.encodeproject.org/search/?type=AntibodyLot&status=released. Each antibody page contains information about the supplier name, catalog number, clone name, lot number and dilution. Each experiment is linked with its corresponding antibody.
Validation	The > 3,000 antibodies that were used are listed on the ENCODE portal at https://www.encodeproject.org/search/?type=AntibodyLot&status=released. Each antibody page contains information about the antibody validation. Antibody characterization guidelines can be found here: https://www.encodeproject.org/documents/4bb40778-387a-47c4-ab24-cebe64ead5ae/@@download/attachment/ENCODE_Approved_Oct_2016_Histone_and_Chromatin_associated_Proteins_Antibody_Characterization_Guidelines.pdf

Eukaryotic cell lines

Policy information about cell lines

Cell line source(s)	We performed assays on 168 cell lines in this study. On the ENCODE data portal each experiment is linked to a specific biosample page with details about the sample source.
Authentication	We performed assays on 168 cell lines in this study. On the ENCODE data portal each experiment is linked to a specific biosample page with details about the sample being authenticated.

Mycoplasma contamination	Cell lines were not tested for mycoplasma contamination.
Commonly misidentified lines (See ICLAC register)	No commonly misidentified cell lines were used.

Animals and other organisms

Policy information about studies involving animals; ARRIVE guidelines recommended for reporting animal research

Laboratory animals	We performed assays on 119 mouse biosamples in this study. On the ENCODE data portal each experiment is linked to a specific biosample page with details about the sample source including species, strain, sex, and age.
Wild animals	None
Field-collected samples	None
Ethics oversight	Not required.

Note that full information on the approval of the study protocol must also be provided in the manuscript.

ChIP-seq

Data deposition

☒ Confirm that both raw and final processed data have been deposited in a public database such as GEO.

☒ Confirm that you have deposited or provided access to graph files (e.g. BED files) for the called peaks.

Data access links *May remain private before publication.*	The ENCODE Portal.
Files in database submission	The ENCODE Portal
Genome browser session (e.g. UCSC)	Track hubs for our data are provided in the supplementary methods.

Methodology

Replicates	See https://www.encodeproject.org/chip-seq/transcription_factor/ and https://www.encodeproject.org/chip-seq/histone/
Sequencing depth	See https://www.encodeproject.org/chip-seq/transcription_factor/ and https://www.encodeproject.org/chip-seq/histone/
Antibodies	See https://www.encodeproject.org/chip-seq/transcription_factor/ and https://www.encodeproject.org/chip-seq/histone/
Peak calling parameters	See https://www.encodeproject.org/chip-seq/transcription_factor/ and https://www.encodeproject.org/chip-seq/histone/
Data quality	See https://www.encodeproject.org/chip-seq/transcription_factor/ and https://www.encodeproject.org/chip-seq/histone/
Software	See https://www.encodeproject.org/chip-seq/transcription_factor/ and https://www.encodeproject.org/chip-seq/histone/
